# Enhancer occlusion transcripts regulate the activity of human enhancer domains via transcriptional interference: a computational perspective

**DOI:** 10.1093/nar/gkaa026

**Published:** 2020-03-05

**Authors:** Amit Pande, Wojciech Makalowski, Jürgen Brosius, Carsten A Raabe

**Affiliations:** 1 Institute of Experimental Pathology, Centre for Molecular Biology of Inflammation (ZMBE), University of Münster, Von-Esmarch-Strasse 56, D-48149 Münster, Germany; 2 Brandenburg Medical School (MHB), Fehrbelliner Strasse 38, D-16816 Neuruppin, Germany; 3 Institute of Bioinformatics, University of Münster, Niels-Stensen-Strasse 14, D-48149 Münster, Germany; 4 Institutes for Systems Genetics, West China Hospital, Sichuan University, Chengdu 610041, China; 5 Institute of Medical Biochemistry, Centre for Molecular Biology of Inflammation (ZMBE), University of Münster, Von-Esmarch-Strasse 56, D-48149 Münster, Germany

## Abstract

Analysis of ENCODE long RNA-Seq and ChIP-seq (Chromatin Immunoprecipitation Sequencing) datasets for HepG2 and HeLa cell lines uncovered 1647 and 1958 transcripts that interfere with transcription factor binding to human enhancer domains. TFBSs (Transcription Factor Binding Sites) intersected by these ‘Enhancer Occlusion Transcripts’ (EOTrs) displayed significantly lower relative transcription factor (TF) binding affinities compared to TFBSs for the same TF devoid of EOTrs. Expression of most EOTrs was regulated in a cell line specific manner; analysis for the same TFBSs across cell lines, i.e. in the absence or presence of EOTrs, yielded consistently higher relative TF/DNA-binding affinities for TFBSs devoid of EOTrs. Lower activities of EOTr-associated enhancer domains coincided with reduced occupancy levels for histone tail modifications H3K27ac and H3K9ac. Similarly, the analysis of EOTrs with allele-specific expression identified lower activities for alleles associated with EOTrs. ChIA-PET (Chromatin Interaction Analysis by Paired-End Tag Sequencing) and 5C (Carbon Copy Chromosome Conformation Capture) uncovered that enhancer domains associated with EOTrs preferentially interacted with poised gene promoters. Analysis of EOTr regions with GRO-seq (Global run-on) data established the correlation of RNA polymerase pausing and occlusion of TF-binding. Our results implied that EOTr expression regulates human enhancer domains via transcriptional interference.

## INTRODUCTION

Transcriptional interference (TI) encompasses cis-regulatory processes involving two adjacent promoters, where the actual regulation is exerted via the act of transcription itself (1). Active RNA polymerases interfere with e.g. pre-initiation complex formation or prevent transcription factor (TF) binding (2–4). TI is correlated with the relative promoter strength: stronger promoters, which initiate transcription at higher (relative) frequency, exert greater impact on regulated downstream promoters (3,4). However, this type of transcriptional control might not be exclusive to TF/DNA interactions within eukaryotic promoter regions; rather all cellular activities that depend on TF/DNA binding could analogously be regulated via TI or related mechanism (5).

High throughput RNA deep sequencing identified different types of non-protein coding transcription within eukaryotic enhancers (6–11). Arguably, the class of enhancer RNAs (eRNAs) represents the most prominent example (9,12–13). The transcripts intersect with enhancer domains and are commonly differentiated according to RNA size and 3′ terminal polyadenylation (9). In general, eRNAs are associated with active enhancer domains; the expression levels of these transcripts and interacting genes are positively correlated (13–15). ‘RNAs with enhancer-like functions’ represent an additional class of transcripts, which—similarly to eRNAs—are preferentially associated with active enhancer domains (6) .

Recently, we identified TFbiTrs (Transcription Factor binding interfering Transcripts) within proximal promoter regions (PPRs: 1 kb upstream regions from the transcription start site [TSS]) of human protein-coding RefSeq (hg19) genes. These transcripts modulate TF/DNA binding via transcriptional interference within intersected PPRs (4). Enhancers are regulatory hubs of clustered TFBSs (9,16–17). Here, we analyzed the potential of TI to regulate the activity of human enhancers. GENCODE cDNA datasets were screened for transcripts that are associated with human enhancer domains and intersected TFBSs of lower relative TF-binding affinity in HepG2 and HeLa cell lines (18,19). These RNAs represent candidates of potentially occluding RNA transcription. Resulting cDNA datasets were further investigated to establish chromatin environments for the occluding transcripts, associated enhancers and regulated genes.

Local enrichments of H3K27ac or H3K9ac histone tail modifications indicate augmented activities of eukaryotic enhancer domains (20–24). Enhancer domains of our datasets were characterized by lower activities as revealed by reduced occupancy levels for either histone tail modification compared to genome-wide controls. In summary, reduced relative DNA binding affinities for transcript-intersected TFBSs and decreased enhancer activities suggested the detection of TI acting on human enhancer domains. Analysis of GRO-seq (Global run-on) datasets established a correlation of RNA polymerase pausing and decreased TF-binding affinities at intersected TFBSs (25). This suggested that the local competition between RNAPII and TF defines the underlying mechanism for TI acting on human enhancer domains. Accordingly, we designated these RNAs as ‘Enhancer Occlusion Transcripts’ or, in short, ‘EOTrs’.

Interactome analysis for regions intersected by EOTrs via ChIA-PET (Chromatin Interaction Analysis with Paired-End-Tag sequencing) or 5C (Carbon Copy Chromosome Conformation Capture) revealed that enhancer domains associated with EOTrs preferentially interacted with poised gene promoters (26,27). These findings emphasized the potential of EOTrs to regulate gene expression within the human genome. All relevant features of EOTrs were compared and contrasted with the more established class of eRNAs within the same cell lines (12,15,28). To the best of our knowledge, we demonstrated, for the first time the regulation of enhancer domains via TI-related mechanism on a genome-wide scale and provided insights into regulatory networks established by EOTrs (5).

## MATERIALS AND METHODS

### Analysis of ENCODE NGS (Next Generation Sequencing) datasets

ENCODE BAM files were analyzed with the edgeR (v 3.24.0) and DiffBind (v 2.10.0, http://bioconductor.org/packages/release/bioc/vignettes/DiffBind/inst/doc/DiffBind.pdf) R-packages (v 3.4.1, https://cran.r-project.org) (29–31). Pre-processing steps for FASTQ files were conducted according to the recommendations of the ENCODE consortium, outlined at https://goo.gl/CgkcGy and https://goo.gl/b2cyAA. Reference to all deep sequencing datasets is provided in Supplementary File 2 Tables S1.1–S1.6 and is repeated at relevant places for convenience.

### CEAS for enrichment of transcripts devoid of known function

The identification of transcripts with unknown function (GENCODE v2 GTF-files) was performed with CEAS (*cis*-element annotation system, v 1.0.2) and RefSeq/HAVANA exon annotations, to exclude cDNA contigs, which overlap with known human mRNAs (Supplementary file Methods) (32).

### Identification of EOTrs—intersection of candidate transcripts with domains of H3K36me3/H3K4me3 and H3K4me1 enrichment

RNA datasets for the identification of EOTrs were the result of two initial filtering steps to provide enrichment for npcRNAs (non-protein coding RNAs) intersecting with human enhancer domains:

1. Intronic and intergenic regions corresponding to polyadenylated and non-polyadenylated long RNAs (GSE26284) were overlaid with domains of H3K36me3/H3K4me3 (GSE29611) enrichment, using BEDTools’ intersectBed command (33,34). Results were statistically evaluated with the Genome Structure Correction (GSC) to test the significance of the observed intersection (Supplementary File 2 Tables S1a–d) (35). Jaccard indices were calculated for the 200 bp flanking regions surrounding (i.e. up- and downstream) the annotated 3′ termini of GENCODE cDNA contigs (Supplementary file Methods, Supplementary File 1 Figure S1). The detection of H3K4me3 peaks aided in the identification of promoter regions (1 kb upstream to cDNA-contigs) for the selected transcripts.
2. Similarly, local intersections of these preselected transcript datasets with domains of H3K4me1 enrichments were corroborated with the aid of the BEDTools’ intersectBed command; resulting datasets were analyzed with the GSC to test the significance of H3K4me1 (GSE29611) compared to H3K4me3 (GSE29611) enrichments for the analyzed domains (Supplementary File 2 Table S2a–d). Transcripts overlapping with bona fide enhancer domains were considered for further analysis (Supplementary file Methods, Supplementary File 1 Figure S1) (36,37).

### Quantification of genome-wide RNA expression

GENCODE cDNA datasets representing polyadenylated and non-polyadenylated total RNA were used to quantify EOTr and RefSeq (hg19) mRNA (messenger RNA) expression levels. For all deep sequencing analysis we utilized RNA-seq datasets, which represented RNA prepared from whole cell lysates. RNA polyadenylation and splicing were identified according to GENCODE GTF files (Supplementary File 2 Table S1.4). Biological replicates were normalized using the calcNormFactors function in R 3.4.1 with the method CPM (CPM [per bin] = number of reads per bin / number of mapped reads [in millions]), to account for apparent differences in feature length (38). Messenger RNA and EOTr expression levels for both cell lines (HepG2 and HeLa) were analyzed with edgeR (v 3.24.0) and featureCounts. BCV (Biological Coefficient of Variation) values between biological replicates within samples and across cell lines were calculated by edgeR’s estimateGLMCommonDisp function (Supplementary file Methods, Supplementary File 2 Table S3) (29,30).

### Coding potential of long transcripts

Long putative ORFs (open reading frames) are absent from non-protein coding sequences (10,11). Therefore, npcRNAs display significantly lower CDS (coding sequence) coverage compared to mRNAs. The protein coding capacity of EOTr candidates was tested with the coding potential assessment (CPAT) tool (v 1.2.2) (Coding Potential (CP) ≥ 0.364 indicates coding sequence, CP < 0.364 indicates non-protein coding sequence, Supplementary File 1 Figure S2) (39). For this analysis, RefSeq CDS (coding sequence) exons (hg19) served as control (Supplementary file Methods, Supplementary File 2 Table S4).

### CAGE cluster analysis

To analyse RNA capping and identify PPRs of EOTr candidates, the 1 kb regions upstream from GENCODE cDNA contigs were intersected with ENCODE CAGE (cap analysis of gene expression, GSE34448) cluster datasets. CAGE clusters were preselected according to HMMs (Hidden Markov Models, IDR-Irreproducible Discovery Rate, scores-0.77/1.00, http://genome.ucsc.edu/cgi-bin/hgTrackUi?db=hg19&g=wgEncodeRikenCage) (40,41). This procedure excludes CAGE clusters not associated with bona fide promoter structures (i.e. false positives) (42,43). Jaccard indices for CAGE clusters intersecting 1 kb up- and downstream regions from annotated transcript 5′ termini of EOTr candidates were compared in order to statistically evaluate the significance for the observed association between both features (Supplementary File 1 Figure S1, Supplementary File 4 section 4.1 and Tables S1a–d, Supplementary file Methods section 2.2 B, RIKEN CAGE: UCSC Genome Browser Track Description) (35).

### ChIP-seq data analysis

URLs to all ChIP-seq datasets are provided in Supplementary File 2, Table S1.3.

### ChIP-seq for histone tail modifications and transcription factors - peak calling

MACS v2 (Model-based Analysis for ChIP-Seq) was utilized for the analysis of genome-wide broad peaks representing histone tail modifications (GSE31477) in EOTr and control regions (44). Replicates were pooled separately for each cell line and modification. Peaks were called with input (mock) DNA samples for identification of unspecific signals. Candidate peaks were selected according to the threshold values: *q*-value ≤ 0.01 and mfold = 10, 100 (default 5, 10) (Supplementary file Methods). The mfold parameter selects only those regions that are mfold or higher enriched for ChIP-seq reads compared to a random genome-wide distribution (fold enrichment for the peak summit against random Poisson distribution computed with the local lambda). Consensus peaks between biological replicates were calculated with DiffBind (v 2.10.0) (Supplementary file Methods) (31).

### Analysis of EOTr PPRs (proximal promoter regions)

Proximal promoters for EOTr candidates were identified within 1 kb upstream regions relative to the representative CAGE cluster peak via the analysis of H3K4me3 enrichments (22,36,45) (Supplementary File 1 Figure S1). Intersections were computed with the BEDTools’ (v 2.14.3) intersectBed command (GSE29611) (33). Resulting H3K4me3 enrichments were compared to those obtained for the corresponding 1 kb downstream regions for the same set of TSSs via the GSC to test the significance of the identified H3K4me3/PPR associations (Supplementary File 1 Figure S1, Supplementary File 2 Table S5a–d).

### Selection of TFs and TFBSs within EOTr loci

Experimentally derived TF-binding sites intersected by candidate EOTrs were identified with the ENCODE transcription factor binding site ChIP-seq data track (https://www.encodeproject.org/chip-seq/transcription_factor/) and CEAS v 1.0.2 (Supplementary file Methods) (46,47).

### Analysis of differential expression for RNA-Seq and ChIP-seq datasets across cell lines

We utilized edgeR (v 3.24.0) and DiffBind (v 2.10.0) for the analysis of differential TF-binding, histone modifications and RNA expression (29–31). edgeR implements generalized linear models, which were utilized for the quantification of effects associated with candidate transcription. The calcNormFactors (edgeR), glmTreat (edgeR with L2FC > 1.5) and DBA_EDGER (DiffBind) functions were utilized for data normalization and further analysis. Results were represented as boxplots and scatterplots (for RNA-Seq and ChIP-seq) via DiffBind (Supplementary file Methods) (48).

### Analysis of active and poised chromatin states, heterochromatin, transcription factor binding and epigenetic domains of eRNA transcription

#### Enhancers

Active enhancers are defined as genomic domains that display combined enrichment for H3K4me1, H3K27ac and/or H3K9ac along with p300 (Supplementary file Methods Table S1.A, GSE29611) (49). They are discernible from poised enhancers by the overrepresentation of intersecting H3K27ac and H3K9ac domains (20,23–24). Overlap of peaks for the above-defined metric was conducted according to the DiffBind (v 2.10.0) protocol (Supplementary file Methods).

#### Promoters

Active promoters display combined enrichments for H3K4me3, H3K4me2, H3K27ac and RNAPII (RNA polymerase II) (Supplementary file Methods, GSE29611) (22,37). Reversely, overrepresentation of H3K4me3 along with H3K27me3 is strongly indicative of poised promoters (Supplementary file Methods). The actual intersection of overlapping peaks within analyzed regions was calculated according to the DiffBind (v 2.10.0) protocol (Supplementary file Methods).

#### Heterochromatin

Enrichments of histone tail modification H3K27me3 (GSE29611) were investigated (i.e. as representative heterochromatin mark) for intronic and intergenic EOTr-associated enhancer regions within HepG2 and HeLa cell lines (37,50) (Supplementary file Methods).

#### Transcription factors

Individual ChIP-seq datasets for TF-binding were retrieved from GEO (GSE31477 and GSE32465, Supplementary File 2 Table S1.2). Broad peaks were detected with MACS v2. Calculations of consensus peaks between replicates were performed with DiffBind protocol and default parameters (Section 7.2 DiffBind vignette). ChIP-seq signals for TF/DNA binding were calculated within 500 bp flanks with p300 peaks as center via the BEDTools’ intersect command (–f 0.8 –r parameters as input [80% and reciprocal overlap], Supplementary file Methods).

#### eRNAs

eRNAs are defined by enrichments for H3K4me1, H3K27ac, H3K9ac, p300 in the absence of H3K36me3 and H3K4me3 signatures (Supplementary File 3 section 3.1 and Table S1) (15,51–52). These combined epigenetic features were utilized to scan human HepG2 and HeLa cell lines for the *de novo* identification of eRNAs. The directionality of eRNA transcription was deduced from the orientation of intersecting CAGE tags and RNA-Seq cDNA data sets that were utilized for eRNA identification (Supplementary File 3 section 3.1 and Table S1).

### Analysis of transcription factor binding and relative binding affinities

URLs to all datasets are provided in Supplementary File 2 Table S1.2.

### Measurement of relative transcription factor/DNA binding affinities

Sequence to affinity prediction tools (STAP) were utilized to quantify the effects of EOTr expression on TF-binding (18). STAP provides as output: (i) The binding parameter (inFactorIntMat), which represents a relative measure of how strongly a TF binds to its corresponding binding sites: values greater than 1 signify ‘favorable’, i.e. stronger, interactions, and less than 1 ‘unfavorable’, i.e. weaker, binding. (ii) The maxBindingWts parameter, which represents the PWM (position weight matrix) scores of analyzed TFBSs and (iii) the Pearson's correlation coefficient for predicted and observed binding scores (expRatios) (18). Analysis of relative affinities of TF/DNA-binding within EOTr and non-EOTr regions was conducted as previously described with the following minor modifications (4).

1. Extraction of binding motifs and calculations of PWMs from ChIP-seq peaks via PscanChIP (v 1.3) and WebLogo were used to generate logos for the resulting TF-binding motifs (Supplementary file Methods) (53,54).
2. Quantification of relative TF–DNA binding affinities in EOTr and non-EOTr regions via STAP.
3. Extraction of binding motifs computed for identical loci across HepG2 and HeLa cell lines (‘analysis across cell lines’) by PscanChIP(54). Pre-computed cell line specific background files representing HepG2 and HeLa cell lines were utilized for the calculation of binding motifs in EOTr and non-EOTr loci for the probed TFs. Resulting motifs were converted into PWMs and considered for further analysis via STAP tools (Supplementary file Methods).
4. STAP output data were displayed graphically with TRAP (Transcription Factor Affinity Prediction) v 3.05 to summarize affinity predictions in EOTr and non-EOTr regions, respectively (19).

### Thresholds of EOTr expression for occlusion of TF-binding

STAP command line option ‘-dt’ aided in the identification of EOTrs associated with sites of favorable or unfavorable TF-binding affinities (4,18). Sorting of expression levels for candidate transcripts associated with favorable and unfavorable binding enabled the identification of expression thresholds, which were the minima required to effectively occlude TF-binding.

### GRO-seq (global run-on sequencing) data

GRO-seq monitors the distribution of active RNA polymerase via the detection of cDNA peaks along the entire genome (25,55–56). We chose this method to analyse RNA polymerase pausing over regulated TFBSs (EOTr+) compared to the entire EOTr-transcribed regions (Supplementary File 2 Table S1.6). Results were confirmed for the same sites and domains employing the analogous analysis with ChIP-seq data for RNAPII with phosphorylated C-terminal domain (CTD, GSE32465 and GSE2735) (Supplementary File 2 Table S1.6). GRO-seq data (sra format, GSM2428726 for HepG2 and GSE62046 for HeLa cell lines) were processed into the FASTQ format with the ‘fastqdump’ command (SRA toolkit) (57). The resulting cDNAs were trimmed with Homer v 4.10 to remove 3′ terminal A-stretches, which had been attached during library construction (homerTools trim). Only cDNAs ≥25bp entered the analysis. Datasets were quality filtered with the FASTX (v 0.0.13) software tool (-q 10 -p 97) (http://hannonlab.cshl.edu/fastx_toolkit/), and resulting GRO-seq cDNAs were aligned to the human genome assembly (hg19) using Bowtie version 0.12.9 (-v 2 -k 3 -m 1 –best) (58). BAM files were utilized to calculate GRO-seq peaks using the annotatePeaks function from HOMER (v 4.10) (59) (Supplementary file Methods).

### Allele-specific expression (AE) of EOTrs and analysis of the accompanying activity of corresponding enhancer domains

GATK (Genome Analysis Toolkit) (v 4.0.1.0) was used for the analysis of allele-specific EOTr expression in HepG2 and HeLa cell lines (60). ENCODE BAM files for RNA-Seq (≥200nt, GSE26284) were analyzed with the ASEReadCounter function, and MAMBA (compatible to GATK v 4.0.1.0) (Supplementary file Methods). By default, each read is counted only once with duplicated cDNA reads being collapsed. RNA editing sites were identified and filtered using SPRINT (SNP-free RNA editing IdeNtification Toolkit v 0.1.8, Supplementary file Methods) (61).

Correlation of the proportion of significant AE sites was carried out using a binomial *P* < 0.05 test for EOTr+ and EOTr– alleles. Active allelic EOTrs were defined by H3K4me1+H3K27ac over-representation. Analysis of differential binding for H3K27ac within EOTr+/EOTr– allelic variants was performed via DiffBind (v 2.10.0) (Supplementary file Methods).

### ChIA-PET and 5C data analysis

URLs to all datasets are provided in Supplementary File 2 Table S1.5. Genome-wide ChIA-PET and 5C data for HeLa and HepG2 cell lines are accessible via GSM970288 and GSM970211. Interactome targets (pairs of interacting enhancers and promoters) were identified using TargetFinder (62).

#### ChIA-PET analysis for HeLa cells

For HeLa, ChIA-PET analysis comprises: (i) linker filtering, (ii) short read mapping, (iii) PET (paired-end tag) classification, (iv) binding site identification and (v) interaction cluster analysis with the ChIA-PET tool v2 (63). The interaction library was derived from RNAPII. ChIA-PET interaction clusters were intersected with EOTr BED regions in HeLa. For subsequent classification, all interactions within EOTr-overlapped regions were scanned for specific enrichment of promoter or insulator marks (ChIP-seq peaks for H3K4me3 and CTCF, respectively). The EOTr interactome was visualized with Circos v 0.69 (64). ChIA-PET data was normalized for differential peak enrichment and genomic proximity using Mango v 1.2.0 and default parameters (Supplementary file Methods).

#### 5C analysis for HepG2 cells

Individual FASTQ replicates representing the 5C interactome for HepG2 cells were aligned to the human genome (hg19) via Bowtie (v 2–2.2.5). SAM output files were converted into sorted BAM with samtools version 1.3.1 (samtools view -bS input.sam | samtools sort -file repX) (65). BAM files were normalized, binned and analyzed for interaction with EOTr loci using the HiFive tool v 1.5.6. (Supplementary file Methods) (66).

### Statistical data analysis

Results were analyzed via the Genome Structure Correction tool (Supplementary file Methods) (35). Functional relationships between any two sets of genomic features are statistically defined on the basis of their proximity/overlap/nearness to each other. Deviations from expected distributions potentially are indicative of biologically relevant associations or non-associations. Datasets in HeLa and HepG2 cell lines were split into intronic and intergenic domains as distribution of analyzed features (size of intronic and intergenic enhancers along with the overlap of other features, e.g. H3K27ac and P300) and their frequency within the genome vary and are dependent on the length of inspected genomic regions. Jaccard indices (the analysis of which is part of the GSC tool package) were estimated as the number of intersections between any two genomic features, divided by their union. The larger the coefficient, the more similar two datasets are in terms of local overlap. Genomic features within analyzed regions were compared to Jaccard indices for the same feature within control regions (domains and associated features are illustrated in Supplementary File 1 Figure S1). Probability values calculated via permutation tests indicated for each region whether the actual overlap was smaller (TRUE) or larger (FALSE) than what would be expected by chance. Results for the relative distance Kolmogorov–Smirnov test, absolute distance test, Jaccard indices and Kullback–Leibler as well χ^2^-tests are detailed in Supplementary File 2 and the main text (35,67).

### Data visualization

All boxplots were drawn with DiffBind (v 2.10.0). Notches indicate 95% confidence intervals (CI) for the median, calculated as ±1.58 × IQR/√*n*. IQR is the interquartile range or distance between the first and third quartiles, where *n* is the number of cells (68). The first and third quartiles relate to the lower and upper hinges of the boxplots (the 25th and 75th percentiles). The upper and lower whiskers extend from the hinge to ±1.5 * IQR of the hinge. Different contrast groups were devised for analysis of histone modification combinations (metric in Supplementary File Methods) within EOTr+ and control groups (EOTr- and eRNAs). Three parameters were considered for data representation via Boxplots for contrast group calculations: (1) Difference of pair-wise group (2) Differences of group mean and (3) Difference of group difference.

## RESULTS

### EOTr candidate datasets and RNA-Seq

**Figure 1.**
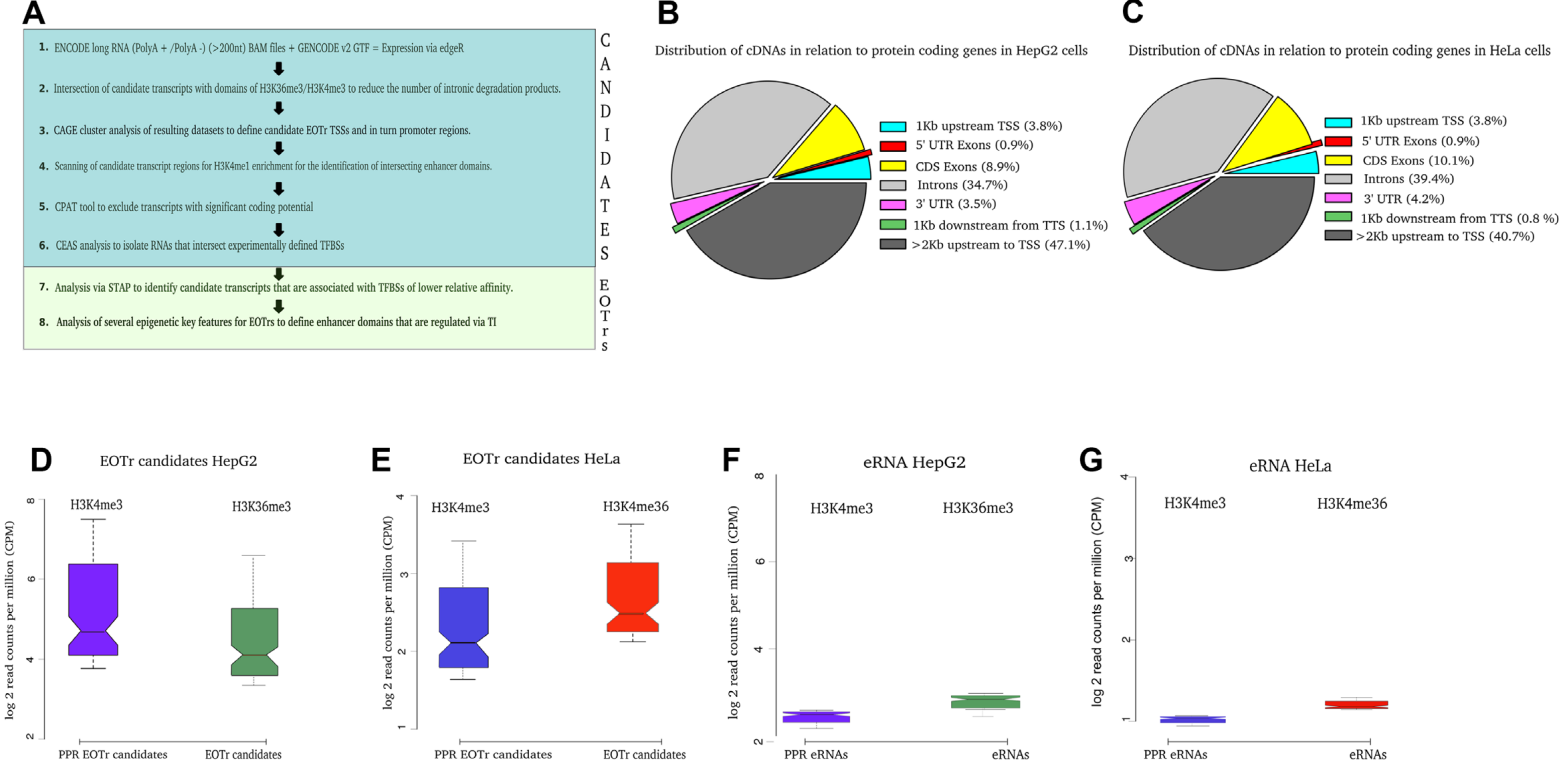
Flowchart representing key steps for the computational identification of EOTrs and the analysis of TI (transcriptional interference) acting on human enhancer domains in HepG2 and HeLa cell lines (**A**). Annotation of GENCODE complementary DNA (cDNA) contigs representing polyA(+) and polyA(-) RNAs with respect to RefSeq protein coding genes (hg19) for human HepG2 (**B**) and HeLa (**C**) cell lines. Annotated features were defined according to CEAS (cis-regulatory Element Annotation System) conventions: TSS = Transcription Start Site, TTS = Transcription Termination Site, UTRs = Untranslated Regions. Only cDNA contigs within intronic (light gray) or intergenic (green and dark gray) regions were selected for analysis. Notched boxplots representing enrichments of H3K4me3 and H3K36me3 histone tail modifications for EOTr candidates and corresponding 1 kb upstream regions (PPRs) for HepG2 (**D**) and HeLa (**E**) cell lines. Results were compared with enrichments for the same histone tail modifications detected within the analogous eRNA regions (**F** and **G**); in contrast to EOTrs, eRNAs were entirely devoid of enrichments for either modification and hence represented a distinct transcript class.

Deep sequencing datasets for polyadenylated and non-polyadenylated RNAs in HepG2 and HeLa cell lines were assembled with GENCODE v2 GTF files and quantified with edgeR (30) (Figure 1A–C). For enrichment of transcripts devoid of known functions, only those RNAs, which did not intersect with available gene annotations, entered the analysis (Table 1). We incorporated CPAT to exclude transcripts, which displayed significant protein coding potential (Figure 1A) (0.364 (Coding Potential ≥ 0.364 indicates coding sequence, CP < 0.364 indicates non-protein coding sequences) (10–11,39). These results were contrasted with training datasets consisting out of RefSeq (hg19) CDS (coding sequence) exons (Supplementary File 1 Figure S2). Finally, 12 093 and 13 191 transcripts in HepG2 and HeLa cell lines defined the input datasets for this survey (Table 1). The resulting cDNA contigs represented polyadenylated and non-polyadenylated long npcRNAs (>200nt) within intronic or intergenic domains of the human genome. For HepG2, 41% were polyadenylated (22% intronic; 19% intergenic) and for HeLa 37.5% (20.9% intronic, 16.6% intergenic). Based on the same GTF files, we established that 43% of these long RNAs in HepG2 (intronic 12%; intergenic 8%) and HeLa (intronic 9%; intergenic 14%) cell lines were spliced transcripts. Resulting datasets are referred to as EOTr candidates or, in brief, candidates.

**Table 1.** Tabulated pipeline for EOTr-detection starting from pools of GENCODE transcripts with unknown functions in HepG2 and HeLa cell lines for intronic and intergenic regions (CTs = candidate transcripts). Only capped and polyadenylated transcripts that intersected TFBSs of lower relative affinity were considered for further analysis and henceforth are referred to as EOTrs

Cell line	Total transcripts	CTs of unknown Function	CTs with H3K36me3 + H3K4me3 intersection	CTs with H3K4me1 intersection	CAGE plus and PolyA + or - CTs	EOTrs: CAGE plus and PolyA plus	Intronic EOTrs	Intergenic EOTrs
HepG2	250794	12093	9345	7341	6782	1647	924	723
HeLa	271239	13191	10752	8766	7358	1958	1145	813

### H3K36me3/H3K4me3 enrichments encompassed regions of candidate transcripts

These preselected GENCODE datasets were further analyzed for enrichments of H3K4me3/H3K36me3 histone tail modifications. Variations of this procedure were previously utilized for detecting long npcRNAs (34,69). H3K36me3 overrepresentations mark domains of active transcription; in addition, this histone tail modification preferentially is associated with exons of actively transcribed genes (70). This analytical procedure, therefore helped to exclude intronic degradation products from input datasets. We analyzed Jaccard indices for the intersection of H3K36me3 histone tail modifications with 200bp flanking regions surrounding the candidate TTS (Table 2, Jaccard *P*-value < 0.01). The comparison revealed the specific association of transcripts (as observed within the 200 bp upstream flanks from the TTS) with H3K36me3 domains (Supplementary File 1 Figure S1, Supplementary File 2 Table S1a–d). No such enrichments were identified within the immediate downstream flanks for the same set of candidate TTSs. Regions 1kb upstream from candidate cDNA contigs were examined for local intersection with domains of H3K4me3 enrichment (37,49). This procedure enabled the identification of proximal promoter regions. The resulting candidate PPRs were intersected with CAGE cluster datasets, which were preselected via HMMs, to provide enrichment for trustworthy promoter structures (Supplementary File 4 Tables S1a–d). About 77% of candidate regions in HepG2 (9345/12 093; intronic 34.3%; intergenic 42.7%) and 81% in HeLa (10 752/13 191; intronic 42.8%; intergenic 38.2%) cell lines, resided within these domains of H3K36me3 and H3K4me3 enrichment (Table 1). Notably, H3K36me3 and H3K4me3 enrichments are not detectable in eRNA-intersected domains (Figure 1D–G), which generally are associated with enhancer domains of higher activity. Our analytical procedure, therefore, provided additional means to exclude RNAs that arguably are not related to transcriptional interference. These data implied that EOTr candidates are bona fide long npcRNAs, which possess their own promoter for independent transcription (71,72).

**Table 2.** Jaccard indices for relevant histone tail modifications for candidate transcripts (CTs), EOTrs and RefSeq genes compared to control regions as indicated. Active promoters were identified by combined H3K4me3/H3K27ac enrichments and poised PPRs (proximal promoter regions) by the overrepresentation of H3K4me3 along with H3K27me3. TTSs = Transcriptional Termination Sites and TSSs = Transcriptional Start Sites

Cell line	Histone modification case	Analyzed Domains case	Jaccard indices for case domains		Histone modification control	Analyzed Domains control	Jaccard indices for control domains	
			Intronic	Intergenic			Intronic	Intergenic
HepG2	H3K4me3	1 kb upstream from CAGE peak for CTs	0.78	0.67	H3K4me3	1 kb downstream from CAGE peak for CTs	0.23	0.09
HepG2	H3K36me3	200bp upstream from the TTS for CTs	0.87	0.84	H3K36me3	200bp downstream from the TTS for CTs	0.15	0.013
HeLa	H3K4me3	1 kb upstream from CAGE peak for CTs	0.70	0.70	H3K4me3	1 kb downstream from CAGE peak for CTs	0.06	0.16
HeLa	H3K36me3	200bp upstream from the TTS for CTs	0.83	0.76	H3K36me3	200bp downstream from TTS for CTs	0.13	0.32
HepG2	Active EOTr promoters	1 kb upstream from CAGE peak	0.73	0.64	Poised EOTr promoters	1 kb upstream from CAGE peak	0.28	0.29
HeLa	Active EOTr promoters	1 kb upstream from CAGE peak	0.76	0.67	Poised EOTr promoters	1 kb upstream from CAGE peak	0.09	0.08
HepG2	Poised RefSeq gene promoters	1 kb upstream from RefSeq TSS	0.88	0.77	Active RefSeq gene promoters	1 kb upstream from RefSeq TSS	0.11	0.21
HeLa	Poised RefSeq gene promoters	1 kb upstream from RefSeq TSS	0.79	0.80	Active RefSeq gene promoters	1 kb upstream from RefSeq TSS	0.12	0.30

### EOTr candidate datasets overlapped with human enhancer domains

We computed the local intersection of candidate transcripts and enhancer domains as revealed by combined H3K4me1/p300 enrichments (22,36–37,73). This analytical procedure identified ∼78% of candidate EOTrs in HepG2 (7341/9345; intronic 43%; intergenic 35%) and 81% in HeLa (8766/10 752; intronic 49%; intergenic 32%) cell lines that intersected with bona fide enhancer domains (Table 1). Jaccard indices and *P*-values calculated via permutation tests underscored this association between features (Jaccard *P*-value < 0.01; Supplementary File 2 Table S2a–d). Enhancer domains, which were not intersected by candidate EOTrs served as a control for all subsequent analysis and are referred to as non-EOTr or EOTr– datasets (non-EOTr regions or EOTr– datasets comprise (i) non-transcribed enhancers and (ii) eRNA-associated domains [Supplementary File 3 section 3.1 and Table S1]; both types of enhancers were characterized by the absence of H3K36me3).

### CEAS and TFBSs intersected by EOTr candidates

Analysis of TI requires count data to quantify RNA expression and to monitor its impact on TF-binding (4,46). We used the ENCODE repository and CEAS to restrict datasets to candidate EOTrs that intersected TFBSs with available ChIP-seq data for HepG2 and HeLa cell lines (32,74). Finally, about 45% (intronic 20%; intergenic 25%) of our preselected candidate datasets in HepG2 and 63% HeLa (intronic 38%; intergenic 25%) cell lines entered the next stage of analysis.

### STAP analysis for relative TF/DNA binding affinities for c-Myc, c-Jun and BRCA1 in candidate EOTr regions

TI is associated with lower relative binding affinities for TFBSs intersected by candidate transcripts (4). We compared TF-binding in relation to RNA expression within candidate EOTr regions with affinities for the same TF in enhancer domains devoid of our transcript datasets (EOTr–). Capped and polyadenylated candidate RNAs, which acted via TI were selected and are referred to as ‘Enhancer Occlusion Transcripts’ (Table 1). STAP tools for the analysis of relative TF-binding affinities revealed that the majority of EOTr-intersected TFBSs, in HepG2 and HeLa cell lines displayed ‘unfavorable’ binding (Table 3, Supplementary File 2 Table S6 for intergenic regions) (18). Indeed, depending on investigated cell line and TF, relative binding affinities (inFactorIntMatscores) were up to 100-times weaker in EOTr domains compared to control regions. Associated PWMs were computed separately for case and control datasets to ensure that detectable differences in TF-binding were not the result of potentially skewed sequence compositions or binding sites of particularly low affinity in case of EOTrs (compare columns 1 and 2 in Table 3). We also utilized the KL-test (Kullback–Leibler) to quantify the difference between distributions for TFBSs between case and control data (Table 4) (67,75).

**Table 3.** STAP (Sequence to affinity prediction) results for c-Myc, c-Jun, and BRCA1 in HepG2 and HeLa cell lines for EOTr and non-EOTr intronic regions. maxBindingWts = PWM scores, inFactorIntMat = favorable (>1)/unfavourable (<1) binding and expRatios = Pearson's correlation (Materials and Methods). The result for the quantification of relative TF-binding affinities for TFBSs intersected by EOTrs consistently revealed unfavorable binding. Notably, the PWM scores were almost identical and suggested that TFBSs within EOTr domains were not of particularly low affinity (maxBindingWts)

		maxBindingWts		inFactorIntMat		expRatios	
Cell line	Transcription factor	non-EOTr	EOTr	non-EOTr	EOTr	non-EOTr	EOTr
**HepG2**	1. c-Myc	81.09	83.69	1.04	0.01	0.71	0.02
	2. c-Jun	78.93	77.52	1.29	0.11	0.66	0.01
	3. BRCA1	81.98	83.69	1.48	0.04	0.82	0.034
**HeLa**	1. c-Myc	78.12	81.44	1.24	0.002	0.64	0.04
	2. c-Jun	67.12	65.20	1.28	0.12	0.75	0.075
	3. BRCA1	88.79	87.97	1.57	0.22	0.68	0.3

**Table 4.** Results of the KL (Kullback–Leibler) test for PWMs (Position Weight Matrix) associated with EOTrs, non-EOTr regions and eRNAs in HepG2 and HeLa cell lines. KL divergence was calculated as discriminant for the non-EOTrs and eRNA datasets

Cell line	KL test EOTrs	KL test non-EOTrs	KL test eRNAs
HepG2	0.18	0.17	0.19
HeLa	0.19	0.21	0.22

**Figure 2.**
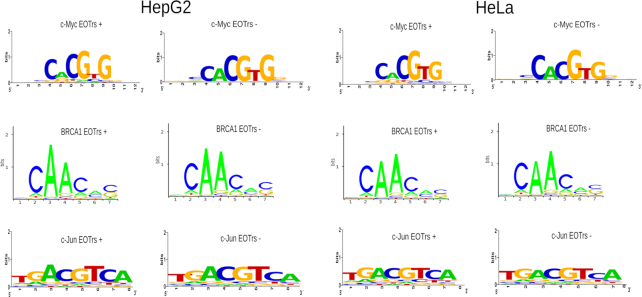
Sequence logos representing TF-binding sites for transcription factors c-Myc, BRAC1 and c-Jun for EOTr+ and EOTr– regions within HepG2 and HeLa cell lines, respectively. PWMs (Position Weight Matrix) for EOTr+ and EOTr– datasets were essentially the same. Confirmatory results were also obtained from the analysis of PWMs with KL (Kullback–Leibler) tests.

**Figure 3.**
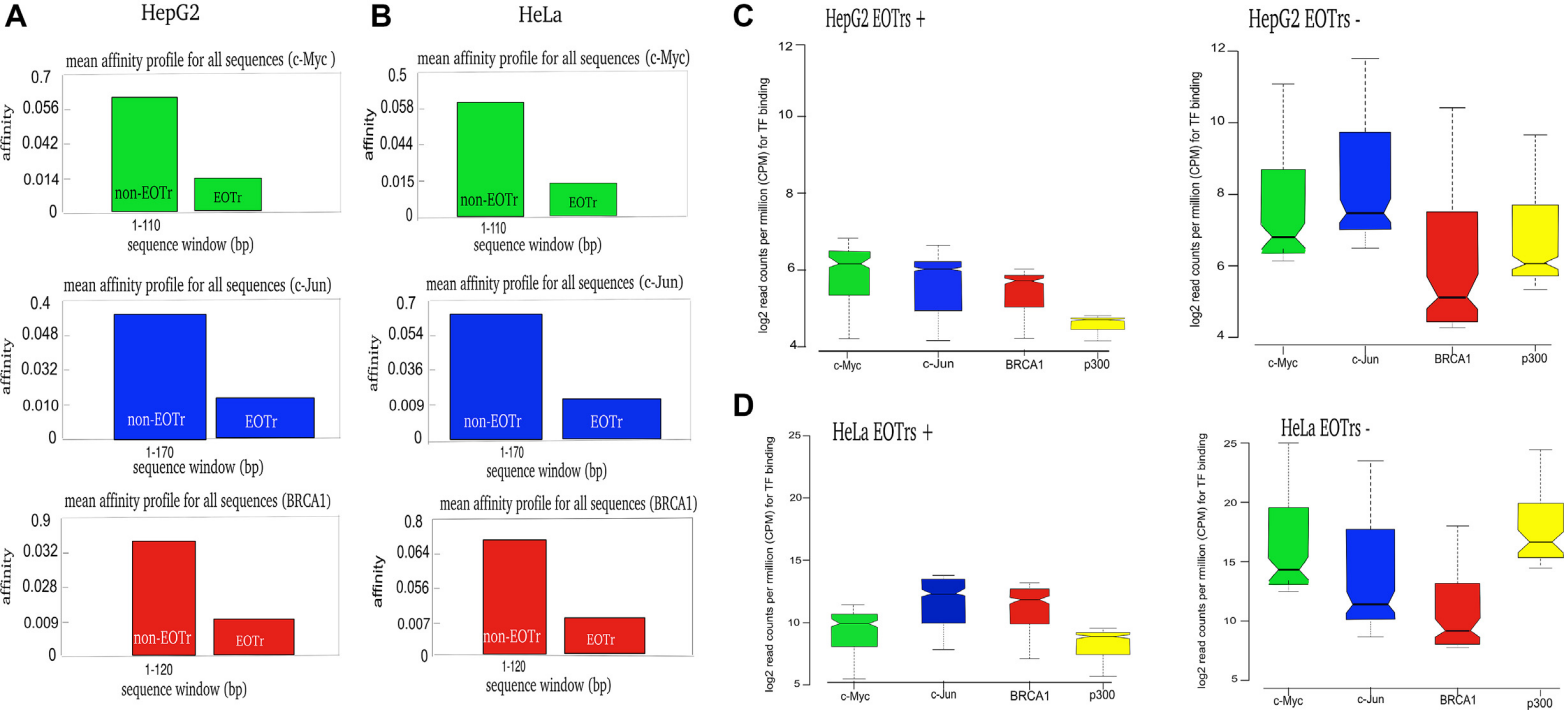
TRAP (Transcription Factor Affinity Prediction) analysis for TFs (Transcription Factor) c-Myc, c-Jun and BRCA1 for EOTr and non-EOTr regions in HepG2 (**A**) and HeLa **(B)** cell lines. Displayed are relative TF/DNA binding affinities for sites intersected by intronic EOTrs (broad peaks). Each graph depicts binding affinities (y-axis) and sequence positions (x-axis) for the investigated transcription factors. Notched boxplots for enrichments of TFs c-Myc, c-Jun, BRCA1 and p300 for intronic EOTr and non-EOTr regions in HepG2 (**C**) and HeLa (**D**) cell lines. ChIP-seq signals for TF/DNA binding were monitored with p300 broad peaks as reference (Materials and Methods). Lower relative binding affinities in EOTr regions (A and B) and reduced TF/DNA occupancies (C and D) for regions intersected by EOTrs suggested TI acting on enhancer domains.

In summary, these results confirmed that the composition of TFBSs within either dataset were similar (Figure 2). Major differences in TF-binding between both datasets were therefore attributable to the presence of potentially interfering RNA expression (given that the size and number of the investigated EOTr and non-EOTr sites were the same), which suggested the detection of TI acting on the intersected TFBSs (Figure 3A and B for intronic EOTrs, Supplementary File 1 Figure S3 for intergenic EOTrs).

Analysis of ChIP-seq signals at TFBSs in EOTr (EOTr+, i.e. TFBSs within enhancer domains overlapping EOTrs) vs. non-EOTr regions (EOTr–) underscored our results (Figure 3C and D for intronic EOTrs, Supplementary File 1 Figure S4 for intergenic EOTrs).

### Analysis of relative TF/DNA binding affinities for TFs c-Myc, c-Jun and BRCA1 within EOTr loci across cell lines

Effects related to EOTr expression were also directly inferred from the calculation of relative TF-binding affinities for sets of identical TFBSs and as a function of occluding transcription (4). Collections of EOTrs, which were expressed in a cell line specific manner (i.e. either in HepG2 or HeLa), served as input data for this analysis (EOTrs with cell line specific expression: HepG2 = 1598 and HeLa = 1909). We ensured that the corresponding TFBSs in both cell lines resided within regions of H3K4me1 and histone acetyltransferase p300 enrichment, suggesting that the analyzed genomic domains acted as bona fide enhancers in both cell lines (36,73).

**Figure 4.**
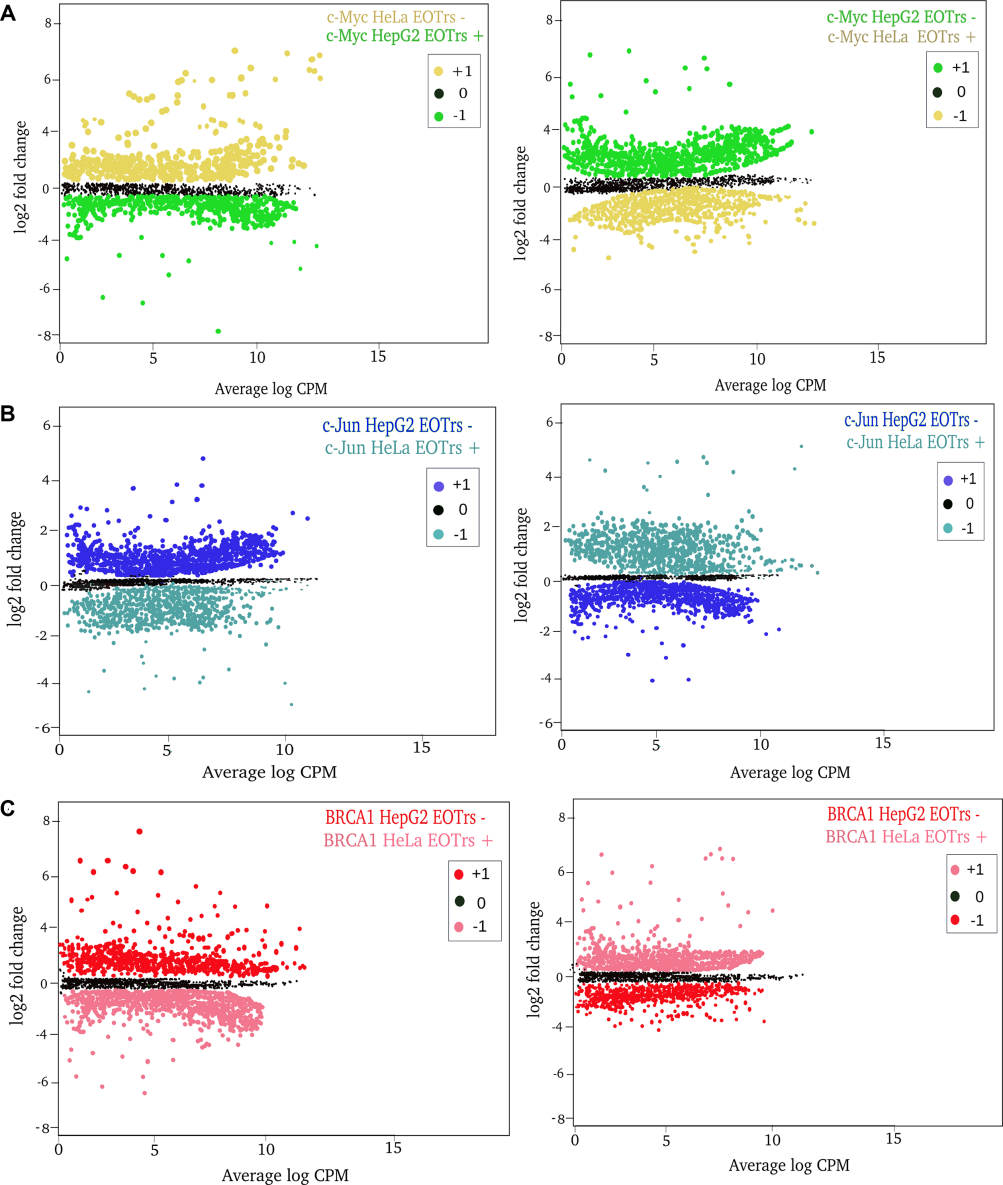
Analysis of differential binding across cell lines: ChIP-seq signals for TF (Transcription Factor)/DNA-binding of c-Myc (**A**), c-Jun (**B**), BRCA1 (**C**) for intronic EOTr regions in HepG2 (left) and HeLa (right) cell lines. TF-binding is compared for the same sets of TFBSs across cell lines in the absence of EOTrs as indicated. ChIP-seq signals for TF/DNA binding were analyzed with p300 (broad peaks) as reference (Materials and Methods). Log base 2 fold change (L2FC) and *P*-value corrected for multiple testing (*q*) (HepG2 → HeLa) *n* = 1431 c-Myc *q* = 1.2 × 10^–9^, *n* = 1579 c-Jun *q* = 2.7 × 10^–5^ and *n* = 1621 BRCA1, *q* = 2.8 × 10^–1^ and HeLa → HepG2, *n* = 1831 c-Myc *q* = 1.2 × 10^–7^, *n* = 1782 c-Jun, *q* = 2.6 × 10^–4^ and *n* = 1911 BRCA1, *q* = 4.7 × 10^–1^. Lower TF/DNA occupancies in EOTr regions suggested the occlusion of TF-binding within enhancer domains intersected by transcript datasets. Also, effects related to EOTr expression were independent of the analyzed cell line. Fold changes: +1 = up-regulated, 0 = not differentially regulated and –1 = down-regulated.

This approach allowed the approximation of TF/DNA interactions in correlation to EOTr transcription, monitored across cell lines. STAP/TRAP analysis tools for quantification of relative TF-binding affinities for the same TFBSs in the absence or presence of EOTr expression (Supplementary file Methods Figure 1C, Table 5 for intronic EOTrs, Supplementary File 2 Table S7 for intergenic EOTrs) revealed ‘unfavorable’ binding for cell lines and TFBSs intersected by EOTrs. Reduced ChIP-seq signal intensities as observed by the analysis of differential TF-binding for TFBSs with overlapping EOTrs confirmed these observations (Supplementary file Methods Figure 1C, Figure 4A–C).

**Table 5.** STAP (Sequence to affinity prediction) results for c-Myc, c-Jun, and BRCA1 across HepG2 and HeLa cell lines for EOTr and non-EOTr intronic regions. maxBindingWts = PWM scores, inFactorIntMat = favorable (>1)/unfavourable (<1) binding and expRatios = Pearson's correlation (Materials and Methods). The analysis quantifies relative TF-binding affinities across cell lines (main text for details). The results revealed that TFBSs intersected by EOTrs were consistently associated with unfavorable binding, suggesting the occlusion of effective TF-binding

		maxBindingWts		inFactorIntMat		expRatios	
Cell line	Transcription factor	EOTr	non-EOTr	EOTr	non-EOTr	EOTr	non-EOTr
**HepG2 TFBS HeLa**	1. c-Myc	87.69	89.75	0.001	1.03	0.04	0.65
	2. c-Jun	74.52	73.50	0.13	1.16	0.001	0.72
	3. BRCA1	93.69	89.72	0.02	1.76	0.034	0.77
**HeLa TFBS HepG2**	1. c-Myc	82.44	87.06	0.03	1.82	0.01	0.59
	2. c-Jun	65.20	67.23	0.12	1.35	0.075	0.83
	3. BRCA1	87.97	84.62	0.22	1.25	0.3	0.69

### Threshold levels for EOTr expression and occlusion of TF-binding

The occlusion of TF/DNA interactions by EOTrs also implied that there might be expression thresholds, which are minimally required for effective TI (4). Depending on cell line and occluded TF, STAP analysis revealed that EOTrs expressed below log_2_ 2.53–3.6 (HepG2) and 3.8–4.2 (HeLa) CPM did not cause the occlusion of TF-binding. Interestingly, for the non-EOTr datasets (eRNAs) expression values ranged between log_2_ 0.42–1.70 (HepG2) and 0.53–1.21 CPM (HeLa) (Supplementary File 3 section 3.2 and Table S2).

### Proximal promoter regions of EOTrs reveal histone tail modifications indicating transcriptional activity

**Figure 5.**
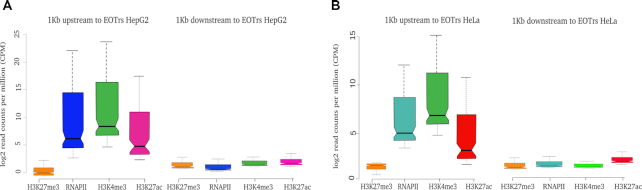
Notched boxplots of histone tail modifications indicative of active transcription analyzed within 1 kb upstream regions for intronic EOTrs in HepG2 (**A**) and HeLa (**B**) cell lines. As a control, enrichments for the same set of histone tail modifications were also monitored within the 1 kb regions downstream of EOTr 3′ termini. EOTrs harbored active promoters within 1 kb upstream regions. This confirmed the correct annotation of EOTr datasets. No such signatures indicative of eukaryotic PPRs were detectable for eRNAs (Supplementary File 3 Figure S1). We concluded that EOTrs and eRNAs represent distinct classes of enhancer-associated transcripts.

Combined enrichments of histone tail modifications H3K4me3 and H3K27ac or H3K4me3 and H3K27me3 characterize active or poised promoters, respectively (37,49). The vast majority of EOTr PPRs, i.e. 89% in HepG2 (1465/1647; intronic 48%; intergenic 41%) and 92% in HeLa (1810/1958; intronic 56%; intergenic 36%) was enriched for H3K27ac, a result, which is strongly indicative of active promoters and, in turn, transcription (Figure 5A and B, left panels for intronic EOTrs, Supplementary File 1 Figure S5A and B for intergenic EOTrs). The association of EOTr PPRs and domains of H3K27ac enrichment was statistically corroborated via the GSC. Jaccard indices (Jaccard *P*-value <0.01) for intronic and intergenic transcripts underscored that the intersection of H3K27ac and EOTr PPRs was specific (Supplementary File 2 Table S8a–d). In summary, these results reconfirmed that our transcript datasets contained active promoters within 1 kb upstream regions. As an additional control, we compared local enrichments for predictive promoter marks including RNA polymerase in 1 kb up- and downstream regions from EOTr TSS and TTS, respectively (Figure 5A and B, right panels for intronic EOTrs, Supplementary File 1 Figure S5C and D for intergenic EOTrs). No enrichments could be established downstream of the TTS.

### Proximal promoter regions of eRNAs display distinct characteristics from EOTrs

Epigenetic footprints of EOTr PPRs were also compared to potential eRNA promoter regions in HepG2 and HeLa cell lines. The corresponding 1 kb upstream regions of eRNAs did not display chromatin environments indicative of active transcription (Supplementary File 3 Figure S1) (15,42). This is consistent with the absence of CAGE clusters preselected by HMMs within the 1 kb upstream regions of eRNAs. The detection of H3K4me3/H3K27ac landscapes within proximal promoter regions of EOTrs also—albeit indirectly—indicated that transcription for these RNAs is initiated outside of intersected enhancer domains. Enhancer RNAs, on the other hand, resided entirely confined within these domains of H3K4me1 enrichment and, hence, are considered to be “enhancer-derived” (7,9,15). In summary, epigenetic footprints for PPRs of eRNAs and EOTrs set these two classes of enhancer-associated transcripts apart, thereby implying alternative routes of RNA generation (Pande *et al.*, in preparation, Supplementary file Methods Figure 1A, Supplementary File 3 Figure S1).

### RNA polymerase pausing: indication of enhancer occlusion

**Figure 6.**
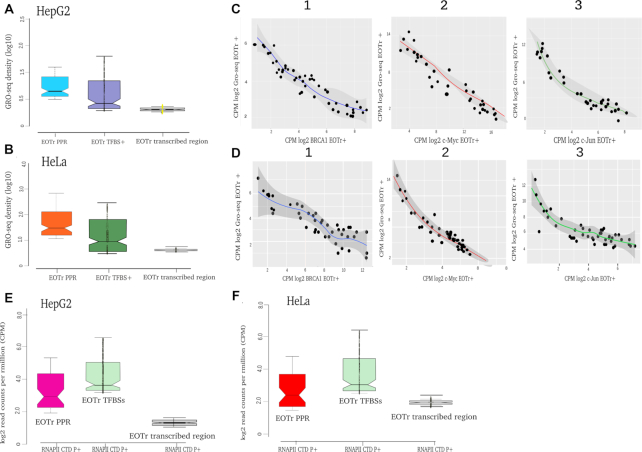
Notched box plots of occupancies for active RNAPII within EOTr-intersected domains for HepG2 (**A**) and HeLa (**B**) cell lines. The analysis uncovered that RNA polymerase II—as demonstrated by GRO-seq signals—is enriched over TFBSs and near the EOTr TSS. The latter is a common feature to RNAPII transcribed genes and served as control. No such enrichments could be identified for EOTr-transcribed regions, indicating the specificity of this enrichment (EOTr-transcribed regions = EOTr-intersected regions flanking TFBSs). Potentially, local competition between RNAPII and TFs for binding to overlapping regions within EOTr-associated enhancers defines the mechanism of TI acting on analyzed domains. Correlation of GRO-seq and ChIP-seq signals for TF-binding (BRCA1, c-Myc, c-Jun) within EOTr domains in HepG2 (**C**) and HeLa (**D**) cell lines. Reverse correlations between RNA polymerase pausing and TF-binding were detectable for all tested TFs and cell lines. Notched box plots representing occupancy levels for transcriptionally active RNA polymerase II (phosphorylated CTD) monitored for EOTr-intersected domains in HepG2 (**E**) and HeLa (**F**) cell lines. Enrichments for RNAPII were highest near the TSS and regions intersecting with occluded TFBSs. Notably, no such enrichments were observed for the corresponding flanking regions.

RNA deep sequencing captures transcript steady state levels within living cells (76). Concentrations, as revealed by the analysis of RNA expression, represent the outcome of several—in part even competing—processes acting on RNA: transcription, processing and degradation. Technologies that specifically monitor the act of transcription provide the most appropriate tools for analysis of TI acting on intersected TFBSs. Many of these methods are based on the original protocol for nuclear run-on assays but represent technical advancements (77); in particular, by incorporating RNA high-throughput sequencing to allow genome-wide analysis. Global nuclear run-on assays coupled with cDNA deep sequencing (GRO-seq) enabled the analysis of RNAPII distributions within EOTr-transcribed domains (25). Complementary DNA peaks representing local enrichments of RNAPII within EOTr-transcribed domains were considered to be indicative of RNA polymerase pausing (55,78). As a control, we utilized the ChIP-seq analysis to determine occupancy levels for phosphorylated RNA polymerase II within the same EOTr-intersected enhancer domains (79,80). Both approaches resulted in the same bimodal distribution of RNAPII with a major peak surrounding the EOTr TSS and a second peak intersecting the occluded TFBSs (Figure 6A, B, E and F). Potentially, TFBSs that overlapped with paused RNA polymerases, are less accessible to TF-binding. Intersections of occluded TFBSs and paused RNAPII were compared to enrichments within immediate flanking regions to demonstrate the specificity of our results. In order to quantify transcriptional pausing for regions of interest, we computed the quotient of binned expression per analyzed feature and the entire EOTr-transcribed domain (pausing index for EOTr-associated TFBSs in HepG2 = 4.8 and HeLa = 4.2 compared to EOTr-transcribed flanking regions in HepG2 = 1.2 and HeLa = 0.9). The data demonstrated that regions containing EOTr-intersected TFBSs were prone to transcriptional pausing. The comparison with eRNAs served as control. In agreement with transcriptional enhancement and higher relative TF-binding affinities in case of eRNAs (Supplementary File 3 sections 3.3–3.4 and Tables S3–S4, Figures S2–S3), we identified no RNAPII pausing within enhancer domains intersected by this class of transcripts (pausing index for eRNA-associated TFBSs in HepG2 = 0.12 and in HeLa = 0.20). Analysis of ChIP-seq peaks for the intersected TFs provided an indirect measure for the affinity of analyzed TFBSs. We uncovered a reverse correlation between RNAPII pausing and TF-binding (Figure 6C and D). These findings are consistent with the portrayed mechanism of TI and suggested that local competitions of RNAPII and TF potentially cause reduced affinities at intersected sites.

### Poised and active enhancers: EOTrs occupy domains of lower activity and TI exerted via EOTrs represents a locally confined mechanism

**Figure 7.**
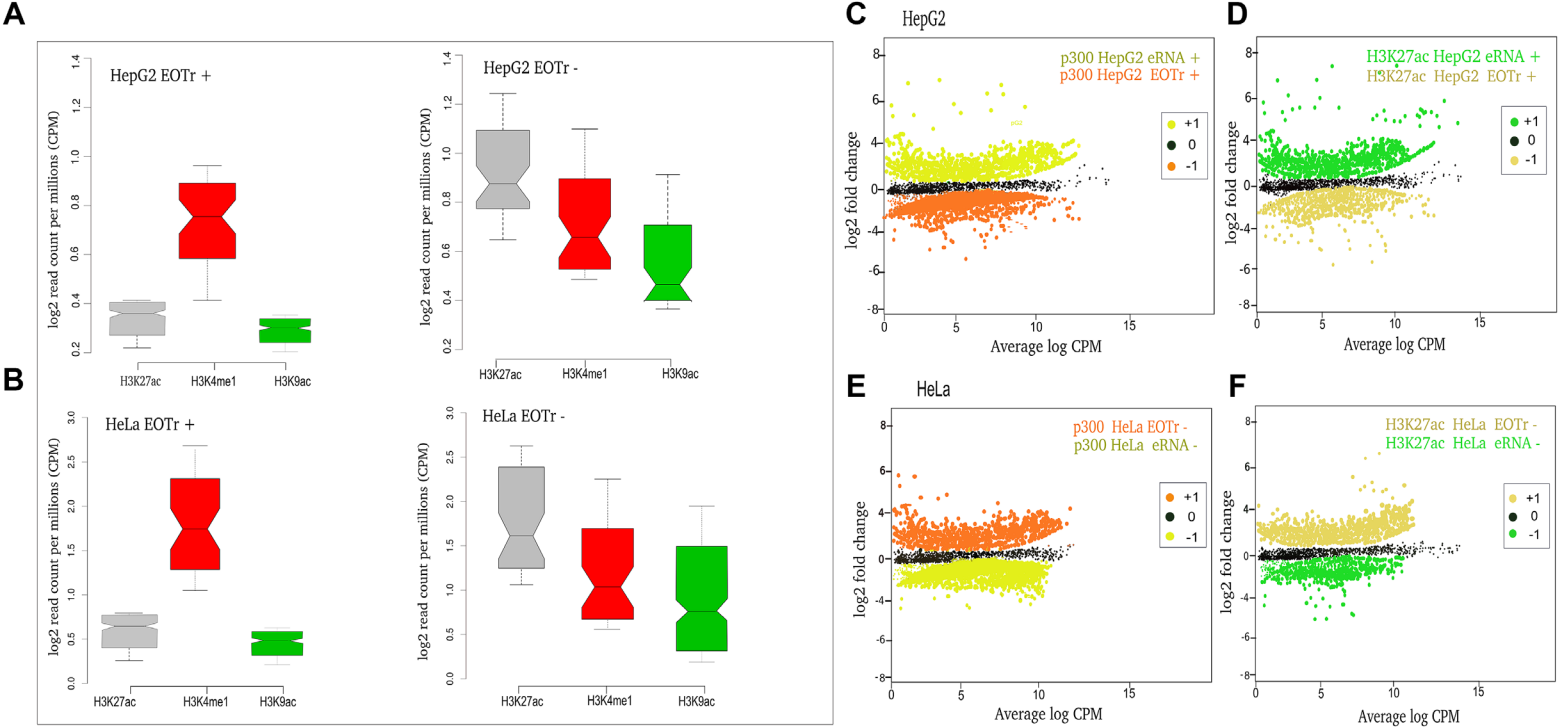
Notched boxplots for H3K9ac and H3K27ac enrichments monitored with p300 peaks as reference for enhancer domains associated with intronic EOTrs (EOTr+) and non-EOTr (EOTr–) regions in HepG2 (**A**) and HeLa (**B**) cell lines. H3K27ac and H3K9ac occupancies were lower for enhancers associated with EOTrs compared to non-EOTr control regions (for definition see main text). Therefore, EOTr-intersected enhancer domains exhibited lower activity. Differential binding across cell lines: enrichments of p300 and H3K27ac were calculated with domains associated with eRNAs or EOTrs in HepG2 cells and the same domains devoid of intersecting transcipts in HeLa cells. Detected fold changes revealed that binding of p300 (**C**) and H3K27ac (**D**) was elevated in eRNA-associated domains. However, in case of EOTr-intersected enhancers within the same cell lines, we observed the reverse correlation: in the absence of EOTrs p300 (**E**) and H3K27ac (**F**) binding was significantly increased. In summary, EOTrs and eRNAs represent functionally distinct classes of enhancer-associated transcription (multiple testing correction p300- HepG2→HeLa *n* = 1027 and HeLa→HepG2 *n* = 1412, Log base 2-fold change (L2FC) and *P*-values corrected for multiple testing (*q*) HepG2 ≥ HeLa, *q*-value = 2.3 × 10^−2^, HeLa ≥ HepG2, *q*-value. = 2.6 × 10^−3^, H3K27ac-HepG2→HeLa *n* = 1347 and HeLa→HepG2 *n* = 1762, Log base 2-fold change (L2FC) and *P*-values corrected for multiple testing (*q*) HepG2 ≥ HeLa, *q*-value = 4.8 × 10^−2^, HeLa ≥ HepG2, *q*-value. = 5.2 × 10^−3^). Fold changes: +1 = up-regulated, 0 = not differentially regulated and –1 = down-regulated.

Enrichments of H3K9ac and/or H3K27ac modifications signify active enhancer domains (24,37,81). Analysis of EOTr-associated enhancers with ENCODE ChIP-seq datasets, revealed significantly lower occupancy levels for both modifications compared with domains devoid of EOTr expression. This was most prominent in case of the enrichments for H3K27ac histone tail modifications and might also be a consequence of lower p300 occupancy levels within EOTr-associated domains (Figure 7A and B for intronic EOTrs, Supplementary File 1 Figure S6A and B for intergenic EOTrs).

In summary, reduced relative TF-binding affinities were accompanied by diminished enhancer activities (Supplementary file Methods Figure S1B). As anticipated, the comparison of EOTr to eRNA-associated enhancer domains revealed that eRNAs populate domains of higher activity (Supplementary file Methods Figure 1A, Supplementary File 3 section 3.5 and Figures S4a and b). Therefore, eRNA but not EOTr expression correlated with activities of intersected enhancer domains. Confirming results were also obtained by the analysis of enhancer activities for EOTr-associated domains across cell lines (see below for intronic EOTrs, Supplementary File 1 section 1.1 and Figure S7 for intergenic EOTrs).

TI acting by the occlusion of TFBSs represents a locally confined mechanism. In the case of EOTr-associated enhancers, on average only ∼60–67% (intronic: HepG2 and HeLa) to 70–73% (intergenic: HepG2 and HeLa) of the entire domain were intersected by our transcript datasets. For the same set of EOTr-associated enhancers, H3K27ac and H3K9ac enrichments were consistently lower for transcript-overlaid domains compared to immediate flanking regions (which are devoid of RNA expression). In line with the mechanism of TI, effects related to EOTr expression were locally confined and barely detectable outside the transcribed regions. The significance of this finding was statistically evaluated with the aid of χ^2^-tests. We analyzed the number of H3K27ac starting peaks for the same enhancer domains within EOTr and non-EOTr regions (i.e. domains downstream from EOTr TTS but contained within the same enhancer). The results underscored the significance (*P* < 0.01) of lower activities associated with EOTr-intersected domains compared to surrounding regions devoid of interfering transcription (Supplementary File 2 Table S9a–d).

### Histone acetyltransferase p300 binding to DNA at EOTr-associated enhancer domains across cell lines

Histone acetyltransferase p300 deposits H3K27ac marks on histones of active enhancers (9,36,81,82). The connection of p300 binding and H3K27 acetylation for EOTr-intersected enhancer domains was further investigated across cell lines. The positive association of eRNAs with domains of higher p300 and H3K27ac enrichment is well established (81,82). Therefore, eRNAs with cell line specific expression served as a control. Analysis of differential binding across cell lines for p300 and H3K27ac indicated that EOTrs preferentially resided within domains of lower activity. Domains devoid of EOTrs were enriched in p300 and concomitantly displayed higher activities (Figure 7C–F). As anticipated, the reverse correlation was observed for the eRNA-associated enhancers in control datasets. Potentially, the higher p300 occupancy levels in the event of eRNA-intersected domains are responsible for the higher activities as revealed by H3K27ac.

These findings agreed with the fact that eRNA transcription did not occlude TF-binding within intersected domains (Supplementary File 3 sections 3.3 and 3.4) and once more established that eRNAs and EOTrs represent distinct classes of enhancer-associated transcription, which possess entirely different regulatory potential.

### Allele-specific RNA expression to monitor effects of EOTr expression on enhancer activities

**Figure 8.**
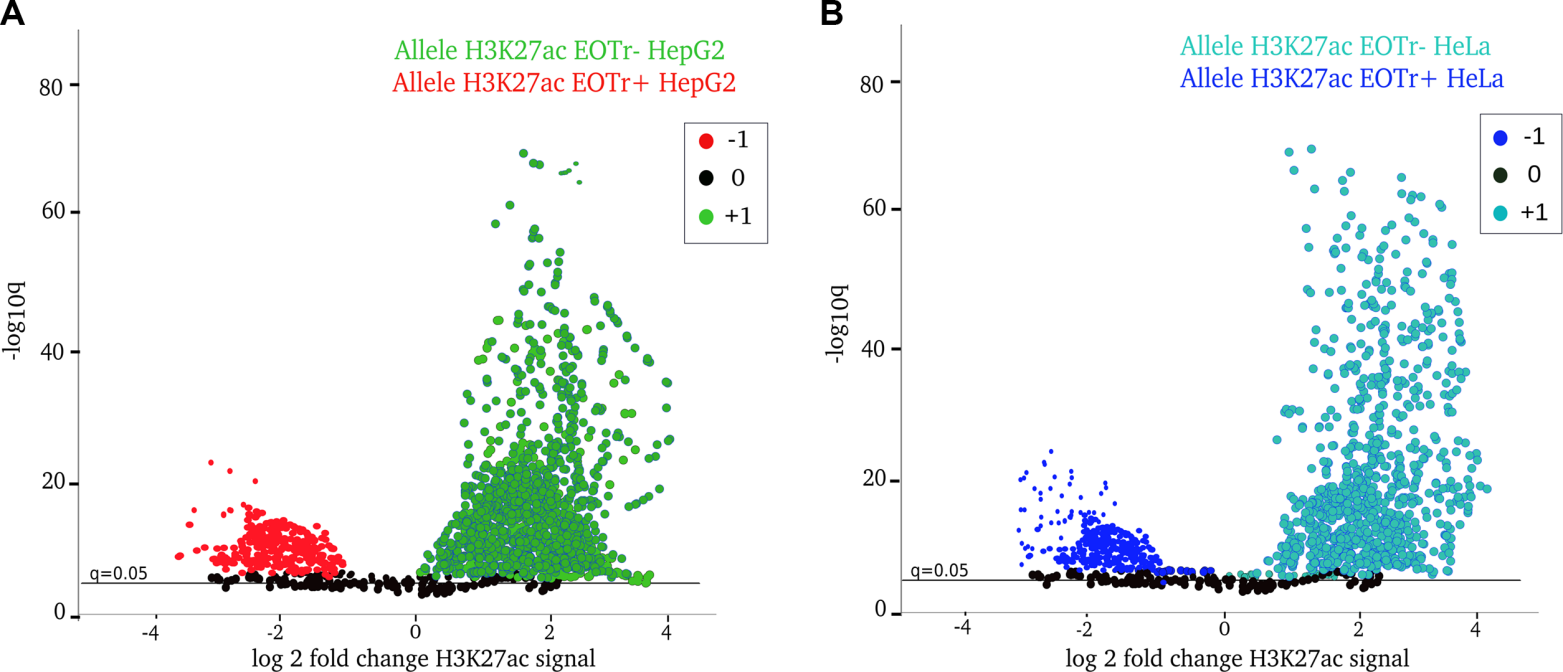
Allele**-**specific differential expression for histone tail modification H3K27ac analyzed within H3K4me1 major peaks for intronic EOTr (EOTr+) and non-EOTr (EOTr–) alleles of the same enhancer for HepG2 (**A**) and HeLa (**B**). Fold changes: +1 = up-regulated, 0 = not differentially regulated and –1 = down-regulated.

In order to further demonstrate the regulatory impact of EOTrs on human enhancer domains, we monitored effects caused by allele-specifically expressed EOTrs (60). With H3K27ac enrichments as analytical read out, enhancer activities for alleles occupied by EOTrs were compared with those devoid of occluding RNA expression (24). This design reflected the outcome of EOTr transcription on virtually the same enhancer domain and cell line. Only domains that displayed H3K4me1/p300 enrichments for both alleles qualified as input data. For RNA deep sequencing data, allele-specific expression and single nucleotide variants (SNVs) were called with GATK and MAMBA tools (Materials and Methods) (60,83). We identified for 47% (intronic 21%; intergenic 26%) and 53% (intronic 28%; intergenic 25%) of EOTr-containing enhancer domains in HepG2 and HeLa cell lines transcripts with allele specific expression. The isolation of 1527 (HepG2 = 814, HeLa 713) SNVs within EOTr-associated enhancer domains permitted the distinction of enrichments for histone tail modifications between individual EOTr alleles. Results for differential H3K27ac binding across alleles, i.e. in the absence or presence of EOTrs, once more suggested the association of lower enhancer activities for alleles intersected by EOTr datasets (Figure 8 and Supplementary file Methods Figure 1D). This further supported our model that EOTrs act via TI at human enhancer domains.

### Interactome (ChIA-PET and 5C) analysis of occluded enhancers

Enhancers and linked promoters reside jointly embedded in networks of, often cell line specific, long-range chromatin interactions (84). Interactome maps enable the characterization of these enhancer domains and their interaction partners. We established the corresponding interactomes for HepG2 and HeLa cell lines with ENCODE 5C and ChIA-PET datasets for EOTr-associated enhancers and their corresponding RefSeq gene (hg19) interaction partners (27,85–87). The vast majority of enhancer domains intersected by EOTrs, i.e. 92% in HepG2 (1515/1647; intronic 51%; intergenic 41%) and 80% in HeLa (1566/1958; intronic 46%; intergenic 34%) were recovered within these interactome maps and preferentially revealed intra-chromosomal looping interactions (intra-chromosomal to inter-chromosomal contact ratio was 5:2). Subsequent investigation of epigenetic footprints associated with these interacting domains discovered enrichments for H3K4me3. This result is strongly indicative of eukaryotic promoters (Materials and Methods, GSC: Supplementary File 2 Tables S10 and 11). The association was statistically evaluated via the GSC (Jaccard index for H3K4me3 compared to CTCF < 0.01). The results confirmed the specificity of our findings (For enhancers associated with cell line specific EOTrs: we identified in HepG2 2343 interacting RefSeq gene promoters [out of these 1327 with intronic enhancers and 1016 with intergenic enhancers] and 3548 interacting RefSeq gene promoters in HeLa [out of these 1812 with intronic enhancers and 1736 with intergenic enhancers]). Therefore, EOTr-associated enhancers participate in the formation of intra-chromosomal looping interactions with human gene promoters.

### Analysis of allele-specific interactomes: the impact of EOTr expression on chromatin looping interactions

**Figure 9.**
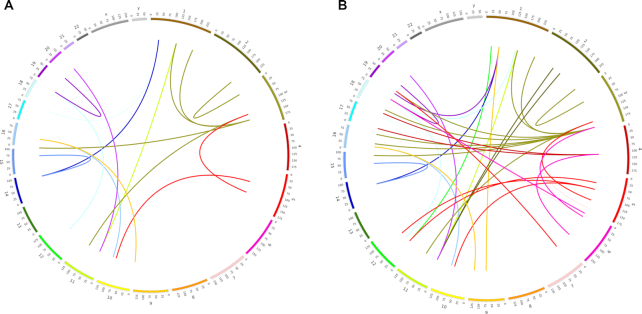
Interactome analysis of intronic enhancers with allele-specific EOTr expression in HeLa cells; interacting loops for EOTr-associated alleles (**A**) are compared to the corresponding alleles devoid of EOTrs (**B**). Enhancer domains devoid of allele specific EOTrs participate in more looping interactions *P*-value < 0.01.

Chromatin interactomes for EOTr loci were investigated in order to identify potential effects related to TI on the formation of enhancer-promoter (E/P) networks. Enhancer domains associated with allele-specific EOTr expression and the corresponding non-EOTr alleles (i.e. enhancer domains devoid of EOTrs for control) were input for this analysis (HepG2-728, HeLa-816). This approach bypassed cell line specific influences on enhancer activities that potentially hamper the analysis across cell lines, and revealed more interacting loci for enhancer alleles devoid of EOTrs (E/P loops EOTr alleles HepG2-1, HeLa-2; non-EOTr alleles HepG2-11, HeLa-23). As a consequence of TI, the resulting interactome complexities, i.e. the number of corresponding E/P interactions, were compromised (Figure 9, χ^2^-test, *P*-value < 0.01).

### RefSeq gene promoters that interact with enhancer domains overlapping EOTrs are predominantly poised

**Figure 10.**
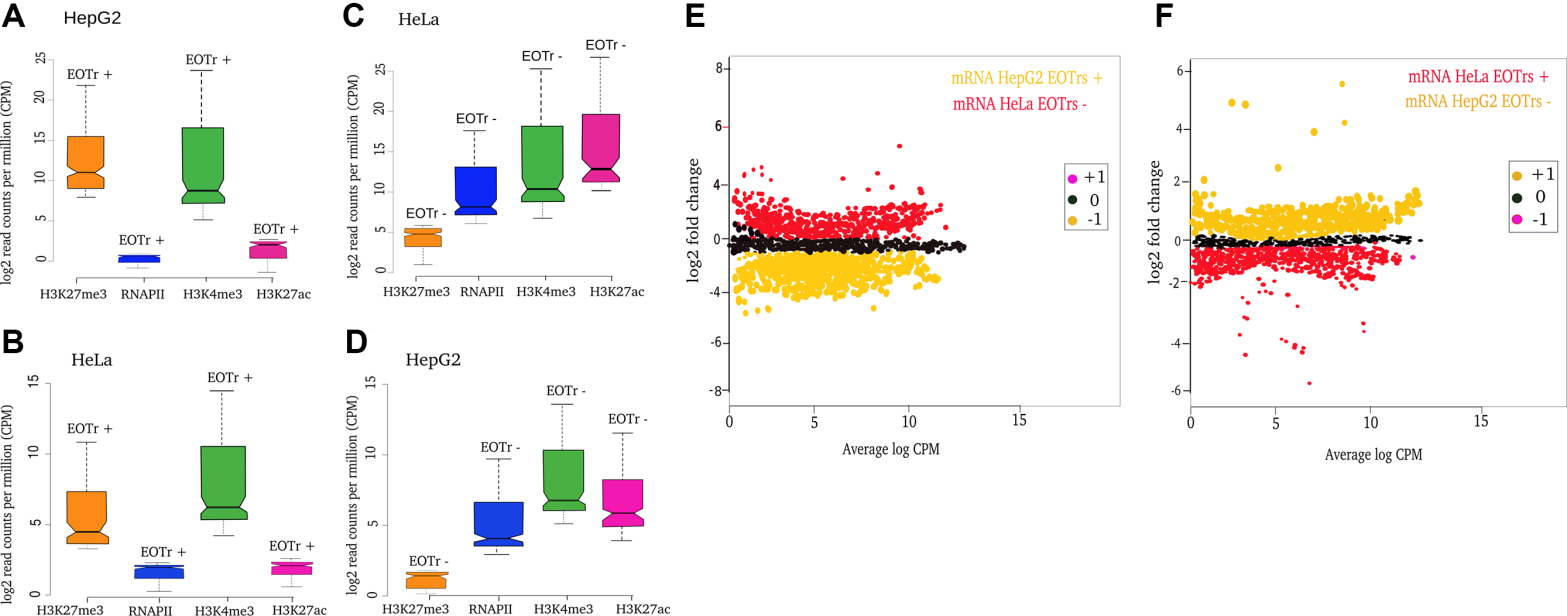
Notched boxplots for histone tail modifications indicative of poised transcription for proximal promoter regions of RefSeq genes regulated by intronic EOTr-associated enhancers in HepG2 (**A**) and HeLa (**B**) cell lines. Analysis across cell lines: (**C**) and (**D**) display results for the analogous analysis with the same set of genes and enhancers but in the absence of EOTrs. Identical RefSeq gene promoters displayed chromatin marks indicative of transcriptional activity when analyzed in cell lines devoid of EOTrs. RNA expression levels of RefSeq genes interacting with intronic enhancers associated with cell line specific EOTrs in HepG2 (**E**) (EOTr+) were compared to the same set of genes in HeLa cells but in the absence of EOTrs (EOTr–). This analysis demonstrated the impact of TI via EOTr-transcription on gene expression. The complementing analysis for EOTr-regulated enhancers in HeLa (**F**) cells is represented on the right (multiple testing correction HepG2→HeLa *n* = 1327 and HeLa→HepG2 *n* = 1812, Log base 2-fold change (L2FC) and *P*-values corrected for multiple testing (*q*) HepG2 ≥ HeLa, *q*-value = 1.3 × 10^−2^, HeLa ≥ HepG2, *q*-value = 1.4 × 10^−3^). Fold changes: +1 = up-regulated, 0 = not differentially regulated and –1 = down-regulated.

As demonstrated, enhancer domains associated with EOTrs displayed lower activities compared to enhancers devoid of EOTrs. We therefore investigated whether this decrease was accompanied by lower promoter activities of linked protein coding RefSeq genes (hg19). ChIA-PET and 5C analysis allowed the identification of interacting gene promoters for HeLa and HepG2 cell lines. Corresponding PPRs were scanned for histone tail modification H3K4me3 in combination with either H3K27ac or H3K27me3, indicative of active or poised promoter states, respectively (45). For proximal promoter regions, which were linked to EOTr-regulated enhancer domains, enrichments of H3K27me3 histone tail modification were uncovered, thereby implying that the corresponding gene promoters (HepG2 = 87%; intronic 52% and intergenic 35% and HeLa = 92%; intronic 58% and intergenic 34%) were transcriptionally silent (Supplementary File 1 Figure S8 for intergenic EOTrs). These results were analyzed with the aid of the GSC to validate the statistical significance of the identified intersection (Table 2, Supplementary File 2 Table S12). To establish this association in broader detail we resorted to the analysis of the same epigenetic footprints for the investigated RefSeq gene promoters across cell lines (compare Figures 10A to 10C and 10B to 10D). In the absence of EOTrs, the corresponding RefSeq gene promoters were embedded within domains of H3K27ac enrichment indicative of active transcription (Supplementary File 1 Figure S9 for intergenic EOTrs). These results demonstrated how the regulation of enhancer domains via TI is transduced to the connected gene promoters. Confirming results were also obtained with the analysis of differential expression for the same set of RefSeq genes. Messenger RNA expression levels were significantly lower for RefSeq genes that interacted with EOTr-regulated enhancer domains (Figure 10E and F).

**Figure 11.**
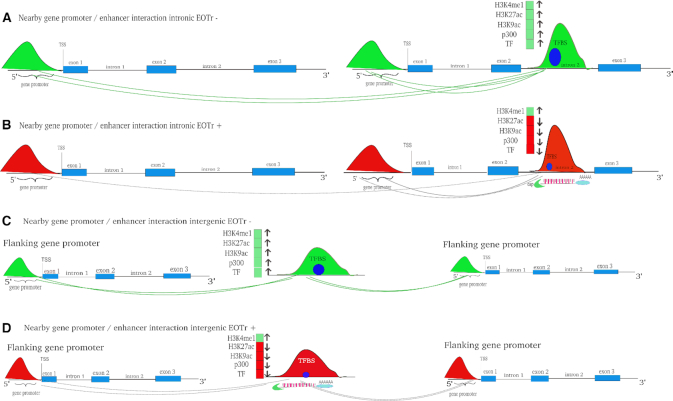
Active intronic enhancer domains and their interactions (green color) with neighboring and ‘host’ gene promoters (see main text) are represented in a cartoon. Intronic enhancer domains devoid of EOTrs (**A**) compared to the same enhancer domains with EOTrs (**B**). Reduced enhancer/promoter interactions and ‘poised activities’ in red color for (B) compared to green in (A). Active intergenic enhancer domains and their interactions (green color) with gene promoters are also represented as cartoon. Enhancer domains, devoid of EOTrs (**C**), compared to the same enhancer domain with EOTrs (**D**). The absence of interacting loops and inactivity of neighboring gene promoters due to EOTr transcription is indicated by dotted lines in (B) and (D).

For intronic EOTr-associated enhancers, ChIA-PET/5C (in HeLa and HePG2 cell lines) analysis revealed that subsets of these intronic enhancers interacted with host gene (host genes are genes with introns containing EOTr-regulated enhancer domains) promoters (63% intronic enhancers in HeLa and HepG2). Interestingly, even these host gene promoters displayed preferentially poised characteristics (Supplementary File 2 Table S13). The resulting network comprising EOTr-associated enhancer and regulated target gene are summarized in Figure 11.

## DISCUSSION

Enhancer domains within multicellular eukaryotes often are transcribed, leading to different classes of potentially regulatory RNAs (6,9,12,14,88). The expression levels of these transcripts are positively correlated with the activity of corresponding enhancer domains and interacting genes (6,23–24). Therefore, enhancer RNAs and ‘RNAs with enhancer-like function’ increase enhancer activity and in turn gene expression. However, for many of these transcript classes, the underlying mechanisms and functions remain subject to further analysis. Enhancer domains represent complex arrays of TFBSs and participate in the formation of pre-initiation complexes for RNA polymerase II transcription. Potentially, RNA polymerase template switching (between promoter and enhancer) could cause spurious transcriptional initiation and, in turn, lead to the generation of these enhancer-derived RNAs. In this context, it is noteworthy that only a minor fraction of elongating RNAPII is associated with conventional promoter dependent transcription (89). A finding, which might suggest that RNA polymerase template switching is a major source of spurious transcription. Notably, eRNAs are not associated with bona fide promoter structures (Supplementary File 3 Figure S1). Therefore, whether and to what extent these enhancer-associated RNAs are by-products of enhancer activity and as such represent products of spurious transcription, still remains to be investigated (9,89–90).

Recently, we uncovered TFbiTrs, transcripts that occlude TF/DNA binding in proximal promoter regions (PPRs) and, in turn, inhibit gene expression (4). Key features of TI encompass the reduction of TF/DNA binding in regions intersected by occluding transcription (3,4). Here, we addressed whether enhancer-associated transcription could also underlie transcriptional interference and reduced activities of intersected domains (5). This process would ultimately lead to the inhibition or reduction of gene expression. We utilized STAP/TRAP analysis for the quantification of relative TF-binding affinities (4,18–19). Relative TF-binding affinities were calculated for the same TF with TFBSs in EOTr (case) and non-EOTr (control) (i.e. TFBSs in enhancers without EOTrs) datasets. The outcome of this analysis depends upon the actual TFBSs and their distribution within both datasets. Therefore, PWMs for TFBSs in EOTr and non-EOTr domains were independently analyzed with KL tests and WebLogo. The high similarities of TFBSs in EOTr and non-EOTr datasets implied that major differences in TF-binding were attributable to EOTr expression acting on otherwise identical TFBSs. Direct analysis of TF-binding as an effect of potentially occluding RNA expression required the comparison of binding affinities for the same set of TFBSs in the absence or presence of EOTrs. For this purpose, we examined EOTrs with cell line and even allele-specific expression. In most EOTr regions, TF-binding was significantly less effective—as revealed by unfavorable binding—compared to the corresponding sites devoid of EOTrs (i.e. across two different cell lines). Apart from EOTr expression, cell line specific heterochromatin states might contribute to the observed TF-binding affinities (91). For the same set of enhancers and cell lines, we compared H3K27me3 enrichments as a marker of facultative heterochromatin, without observing significant differences (Supplementary File 1 section 1.2 and Figure S10). Consequently, altered heterochromatin states between cell lines did not impact on the analysis of relative TF-binding affinities via STAP.

Enhancer activities are positively correlated with H3K27ac enrichments (22,24). The analysis for EOTr-associated enhancer domains, in relation to H3K27ac occupancy levels, was conducted in three-fold: (i) within HepG2 or HeLa cell lines in comparison to non-EOTr enhancers, (ii) across cell lines for the same enhancers and (iii) for allelic pairs. Our results consistently indicated that enhancers associated with EOTrs exhibited significantly lower activity levels. Therefore, EOTr expression is not only reflected by reduced relative binding affinities; in addition, we identified that TI is even correlated with activity levels of intersected enhancer domains.

Pervasive transcription within genomes generates sites of overlapping or interleaved transcription (9,92). The enhancer landscape occupies a substantial fraction of the intronic or intergenic sequence space. The presented analysis was conducted with only three TFs, which were utilized to identify occluding RNA transcription within the human genome. The results, therefore, represent a subset of all occluding transcripts. Potentially, there are many more EOTr-like transcripts, and we propose that TI or related mechanisms could even account for a considerable fraction of the RNA ‘dark matter transcription’ (93,94).

We propose that EOTrs, as enhancer regulating transcripts, are highly flexible molecular tools and as such could serve many functions depending on the regulated enhancer module or occluded TF. Future analysis with more comprehensive datasets will enable to address their functional relevance in broader detail. Also, gene expression in HepG2 and HeLa cell lines is substantially different from non-cancer tissues (95,96). The extrapolation of EOTr functions to non-cancer cells or intact organisms is therefore tentative.

In case of TI, the act of transcription itself is accompanied by the local competition between TFs and RNAPII for DNA binding within overlapping regions. This mechanism could be the underlying cause of lower TF-binding affinities in EOTr regions. To test these assumptions, we inspected the correlation of RNA polymerase pausing and TF-binding within EOTr-intersected domains (55,79–80). The identification of reverse correlations for RNA polymerase pausing and TF-binding argues in favor of TI as the defining mechanism of EOTr action. The concept of RNA decoys for transcription factors is less likely to explain EOTr functions because RNAs are diffusible macromolecules (90,97,98). Accordingly, EOTrs would sequester available TFs and globally reduce the effective concentration of free nuclear TFs. This hypothetical mode of EOTr/TF action would alter TF-binding even in non-EOTr regions. Consequently, there should be no detectable difference for TF-binding at EOTr-intersected TFBSs and sites within domains devoid of EOTrs. Similarly, lower occupancy levels for H3K27ac histone tail modifications were strictly confined to domains associated with EOTrs. This local restriction of apparent EOTr-related effects is consistent with TI as opposed to mechanisms based on sequestration, which are not limited to certain domains or binding sites. To gain further insights into the regulation of human enhancer domains via transcription, we analyzed and compared two entirely different transcript classes: EOTrs, which act via TI and eRNAs (for identification and analysis of eRNAs in HepG2 and HeLa cell lines, Supplementary File 3 sections 3.1–3.6) that map to domains of higher enhancer activity (23–24,37,49). Initially, this observation might seem contradictory, prompting questions as to why eRNAs do not occlude TF-binding in ways similar to EOTrs. TI (or related mechanisms) depends on the relative promoter strength of occluding RNAs and only promoters that initiate transcription at sufficiently high rates are capable to occlude TF-binding (3,4). Once more, we used STAP/TRAP analysis tools for the identification of expression thresholds that cause TI. Expression levels of eRNAs within HepG2 and HeLa cell lines ranked substantially below these critical thresholds (Supplementary File 3 section 3.2 and Table S2). This finding might, at least in part, explain why EOTrs but not eRNAs are capable of occluding TF-binding within the analyzed domains. Congruently, relative binding affinities for TFBSs within eRNA domains suggested favorable binding compared to EOTrs as well as genome-wide controls (Supplementary File 3 sections 3.4 and 3.5). Lower expression levels of eRNAs were also mirrored by the epigenetic landscapes that typically were associated with this class of transcripts: For instance, the substantially lower occupancy levels for H3K36me3 within transcribed regions. In addition, the 1 kb upstream regions of eRNAs lacked H3K4me3 signatures (Figure 2 and Supplementary File 3 Figure S1). EOTrs, on the other hand, displayed epigenetic key features of long npcRNA transcription and hence differ substantially from the enhancer derived eRNA transcripts (9,15,34).

Decreased activities for EOTr-associated enhancers, were correlated with reduced enhancer/promoter interactions as revealed by ChIA-PET and 5C. Fewer interacting loops were identified for EOTr-intersected enhancers per regulated promoter than for enhancer domains devoid of the occluding transcription. Therefore, even enhancer/promoter interactions are reduced in number and might be inhibited by interfering EOTr expression (Supplementary File 2 Table S14a and b). Notably, RefSeq gene promoters that interacted with EOTr-associated enhancer domains displayed poised characteristics. This association, however, is entirely reversed when the same PPRs were analyzed in the absence of regulatory EOTr expression. In this case, enhancers and corresponding RefSeq gene promoters were embedded in domains of transcriptional activity. In contrast, RefSeq genes linked to enhancer domains associated with eRNAs consistently ranked amongst the highly expressed genes (Supplementary File 3 section 3.6 and Figure S5). To the best of our knowledge, this analysis represents the first genome-wide investigation of TI-related mechanisms acting on human enhancer domains (Supplementary File 1 Figure S11). EOTrs add a further level to the intricate regulation of eukaryotic networks of gene expression.

## Supplementary Material

gkaa026_Supplemental_Files

## Notes

Present address: Amit Pande, Max-Delbrück-Centrum für Molekulare Medizin (MDC), Robert-Rössle-Str. 10, 13092 Berlin, Germany.
