# Supplementary material for: Enhancer occlusion transcripts regulate the activity of human enhancer domains via transcriptional interference: a computational perspective
Source: Nucleic Acids Res. 2020 Mar 5;48(7):3435–54. doi: 10.1093/nar/gkaa026 (PMC7144904; doi:10.1093/nar/gkaa026)
Supplement: gkaa026_Supplemental_Files [file gkaa026_supplemental_files.zip › Supplement_File_3_NAR_01_03_2020.docx]

**3.1 Introduction**

EOTrs do not represent the only class of enhancer-associated, (potentially) regulatory transcripts in the human genome. eRNAs (enhancer RNAs) are distinct from EOTrs, as the former occupy active enhancers (1-4) [devoid of detectable H3K36me3 enrichments (5,6). Because of these biological features, we utilized this class of RNAs as control group for our EOTr analysis. Also, the comparison of these distinct transcript classes might enable a better understanding of enhancer-associated transcription. At the time of analysis, there were no comprehensive eRNA annotations available for HeLa and HepG2 cell lines. We therefore resorted to the below defined analytical protocol for eRNA identification (Supplementary File 3 table S1)](#_ENREF_1) (5-9)[, which also enabled the analysis of eRNAs and EOTrs with the same datasets. Similar to EOTrs the following key features were characterized: landscapes of eRNA proximal promoter regions, relative TF-binding affinities of intersected TFBSs, expression levels of eRNAs and genes interacting with eRNA-associated enhancer domains. Our computational analysis relied on the exact same set of tools that are already described for EOTrs in the main text, the Material and Methods section and its accompanying supplement: edgeR](#_ENREF_5) (10), DiffBind (11)[, STAP/TRAP](#_ENREF_10) (12,13) and the GSC[.](#_ENREF_12)

**Supplementary File 3 table S1.** Metrics to define active and poised eRNAs in HepG2 and HeLa cell lines.

| Active eRNAs | Poised eRNAs | | +  +  -  -  -  - |  | | |
| --- | --- | --- | --- | --- | --- | --- |
| (A) H3K4me1 | + | 1. H3K4me1 |  |  |  |  |
| (B) H3K9ac | + | 1. H3K27me3 |  |  |  |  |
| (C) H3K27ac | + | (C) H3K27ac |  |  |  |  |
| (D) p300 | + | (D) H3K9ac |  |  |  |  |
| (E) H3K36me3 | - | (E) H3K36me3 |  |  |  |  |
| (F) H3K4me3 | - | (E) H3K4me3 |  |  |  |  |
|  |  | |  | |  |  |
| **Cell line** | **eRNAs** | | **CAGE HMM / eRNA with CAGE HMM** | | **Bidirectional eRNAs** | **Unidirectional eRNA** |
| HepG2 | 3123 | | 330094/ 32 | | 2789 | 334 |
| HeLa | 2976 | | 191018/ 21 | | 2138 | 838 |

**3.2 RNA expression levels offer a possible explanation for opposing effects caused by different classes of enhancer-associated RNA transcription**

Regulatory effects of transcriptional interference (TI) are correlated with the expression levels of the occluding RNAs (14-17)[. We therefore analyzed and compared expression levels of eRNAs and EOTrs. Results indicated that for the same cell line eRNA expression is consistently lower than that of EOTrs (Supplementary File 3 table S2). Comparison of threshold levels that were minimally required for the occlusion of TF/DNA interactions in case of EOTrs, suggested that eRNAs are expressed below these critical levels (actual levels for eRNA and for EOTrs) and hence do not cause the occlusion of effective TF-binding (STAP analysis main text). This observation provides a possible explanation of why eRNAs might not be capable to interfere with effective TF-binding](#_ENREF_14) (Supplementary File 3 table S2).

**Supplementary File 3 table S2.** Comparison of EOTr and eRNA expression levels in HepG2 and HeLa cell lines.

| **Cell line** | **EOTr (CPM per bin)** | **eRNA (CPM per bin)** |
| --- | --- | --- |
| **HepG2** | 2.53-3.6 | 0.42-1.70 |
| **HeLa** | 3.8-4.2 | 0.53-1.21 |


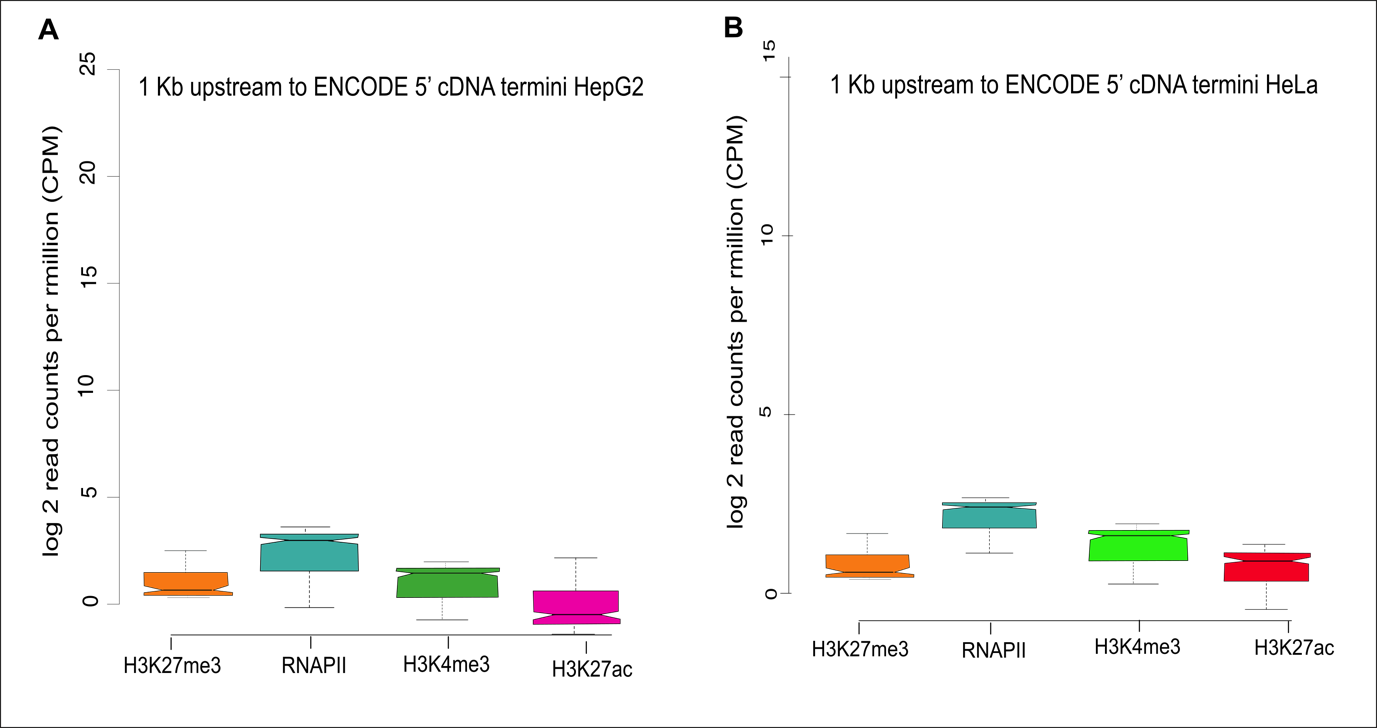


**Supplementary File 3 figure S1.** Notched box plots for ChIP-seq signals of histone tail modifications analyzed within potential PPRs (Proximal Promoter Regions, i.e., the 1Kb upstream regions to annotated 5’ termini of ENCODE cDNA contigs) of eRNAs for HepG2 (**A**) and HeLa (**B**) cell lines. The comparison of these epigenetic footprints with those of our candidate datasets (Figure 1 main text) revealed that EOTrs represent a separate class of RNA transcripts.

**3.3. STAP/TRAP analysis to delineate relative transcription factor binding affinities for eRNA-intersected enhancer domains compared to EOTr-containing counterparts in HeLa and HepG2 cell lines**

Elevated enhancer activities, presumably, are also reflected in higher relative TF-binding affinities for TFBSs within eRNA-intersected domains compared to genome-wide control datasets. As a control, we resorted to enhancers with EOTrs or those devoid of any detectable RNA expression. Analysis via STAP/TRAP (Sequence To Affinity Prediction/Transcription Factor Affinity Prediction) for quantification of relative TF-binding affinities (Materials and Methods) within eRNA and EOTr domains, revealed favorable binding for TFs c-Myc, c-Jun and BRCA1 in eRNA regions (12,13) (Supplementary File 3 table S3 and Supplementary File 3 figure S2). These results demonstrated that TFBSs intersected by the eRNAs displayed higher relative binding affinities. The STAP/TRAP analysis was also reconfirmed by ENCODE ChIP-seq data for TFs c-Myc, c-Jun and BRCA1 (Supplementary File 3 figure S3).

.

**Supplementary File 3 table S3.** STAP (Sequence To Affinity Prediction) analysis for c-Myc, c-Jun and BRCA1 transcription factor/DNA binding for intronic enhancer domains with EOTrs and eRNAs in HepG2 and HeLa cell lines: maxBindingWts=PWM scores, inFactorIntMat=favourable (>1)/unfavourable (<1) binding and expRatios=Pearson’s correlation (Materials and Methods). Favorable binding of TFs within eRNA regions compared to EOTrs differentiates enhancer domains of either dataset.

| Cell line | Transcription factor | | maxBindingWts | | inFactorIntMat | | expRatios | |
| --- | --- | --- | --- | --- | --- | --- | --- | --- |
|  |  |  | eRNA | EOTR | eRNA | EOTrs | eRNAs | EOTrs |
| HepG2 | 1. | c-Myc | 89.21 | 87.69 | 1.31 | 0.001 | 0.81 | 0.04 |
|  | 2. | c-Jun | 71.93 | 74.52 | 1.77 | 0.13 | 0.76 | 0.001 |
|  | 3. | BRCA1 | 80.72 | 81.69 | 1.92 | 0.02 | 0.66 | 0.034 |
|  | . |  |  |  |  |  |  |  |
| HeLa | 1. | c-Myc | 89.36 | 87.44 | 1.36 | 0.03 | 0.78 | 0.01 |
|  | 2. | c-Jun | 79.32 | 75.20 | 1.89 | 0.09 | 0.81 | 0.05 |
|  | 3. | BRCA1 | 83.29 | 87.97 | 1.35 | 0.22 | 0.59 | 0.3 |

PWMs (position weight matrix) for case and control datasets were established independently and the KL-test confirmed that the distribution of TF-motifs within either dataset were almost the same (Table 4 main text). The fortuitous association of eRNA-associated enhancers with TFBSs of higher relative affinity is therefore less likely.


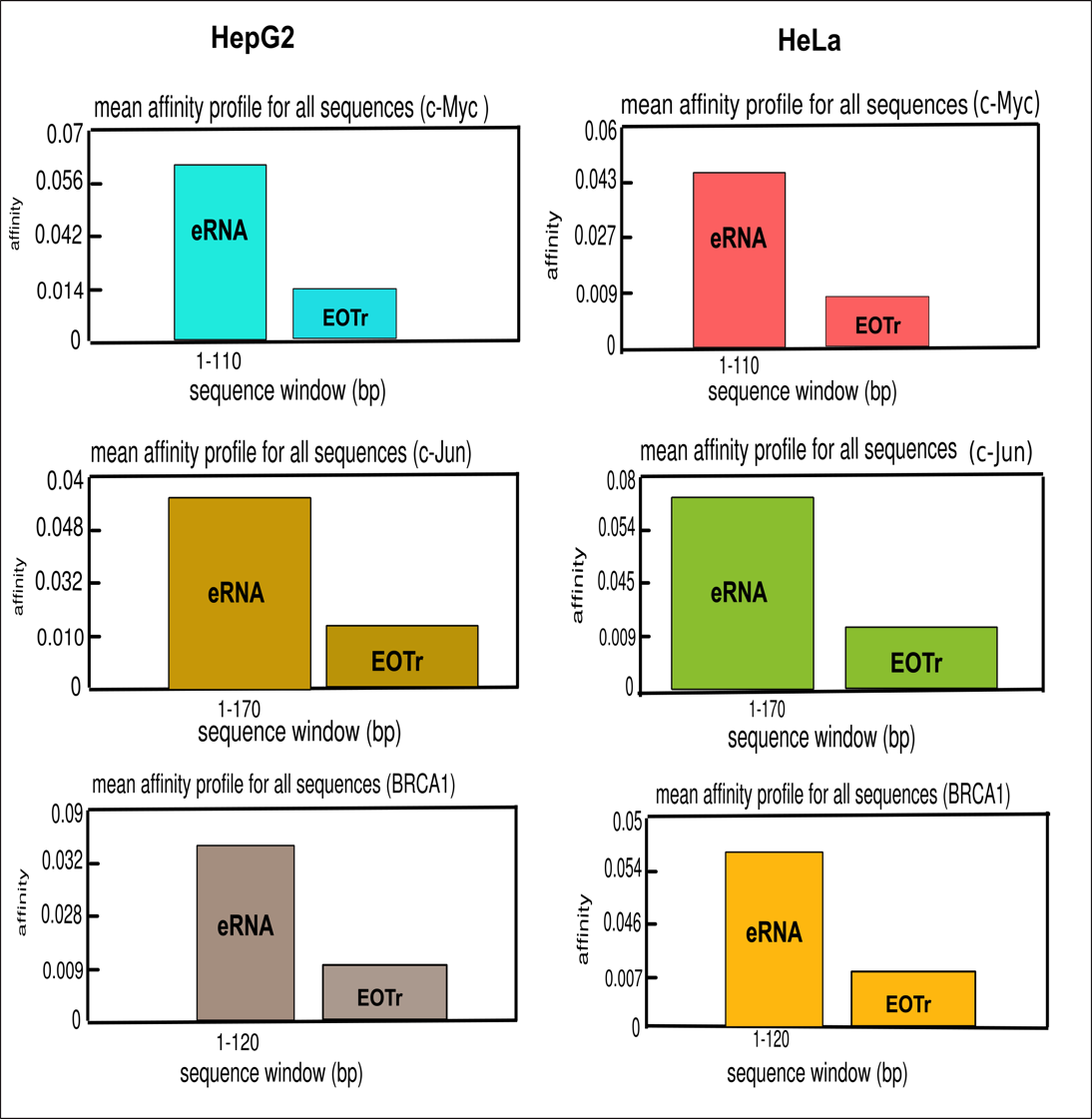


**Supplementary File 3 figure S2a.** TRAP (transcription factor affinity prediction) analysis of relative DNA-binding affinities for TFs (transcription factor) c-Myc, c-Jun and BRCA1 for intronic enhancer domains intersected by EOTrs or eRNAs in HepG2 (left) and HeLa cell lines (right). Each graph displays relative DNA binding affinities (y-axis) and sequence positions (x-axis) for the indicated transcription factors (broad peaks). Lower relative binding affinities in EOTr regions differentiate effects correlated with EOTr and eRNA transcription.


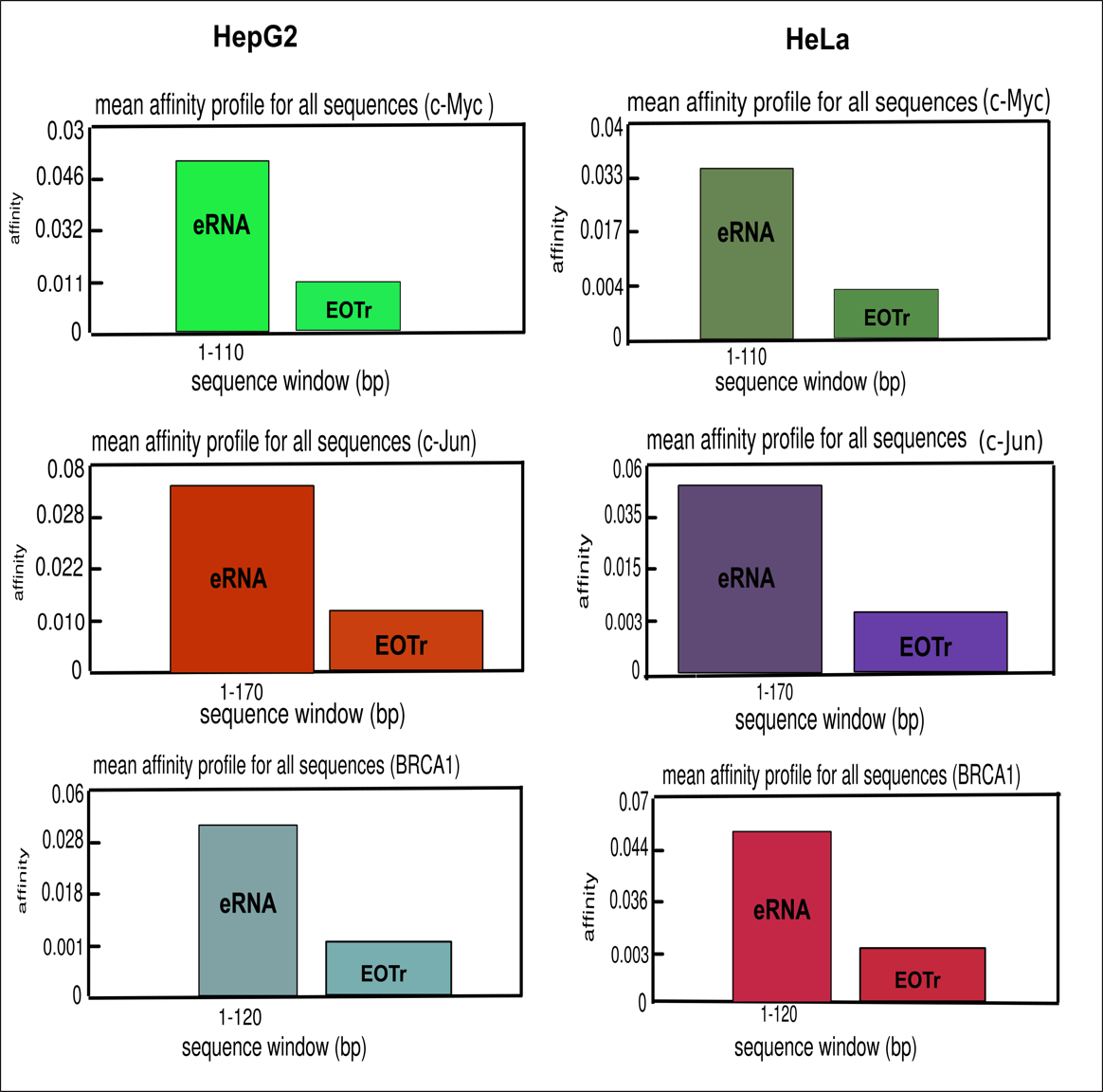


**Supplementary File 3 figure S2b.** TRAP (transcription factor affinity prediction) analysis of relative DNA-binding affinities for TFs (transcription factor) c-Myc, c-Jun and BRCA1 for intergenic enhancer domains intersected by EOTrs or eRNAs in HepG2 (left) and HeLa cell lines (right). Each graph displays relative DNA binding affinities (y-axis) and sequence positions (x-axis) for the indicated transcription factors (broad peaks). Lower relative binding affinities in EOTr regions differentiate effects correlated with EOTr and eRNA transcription.


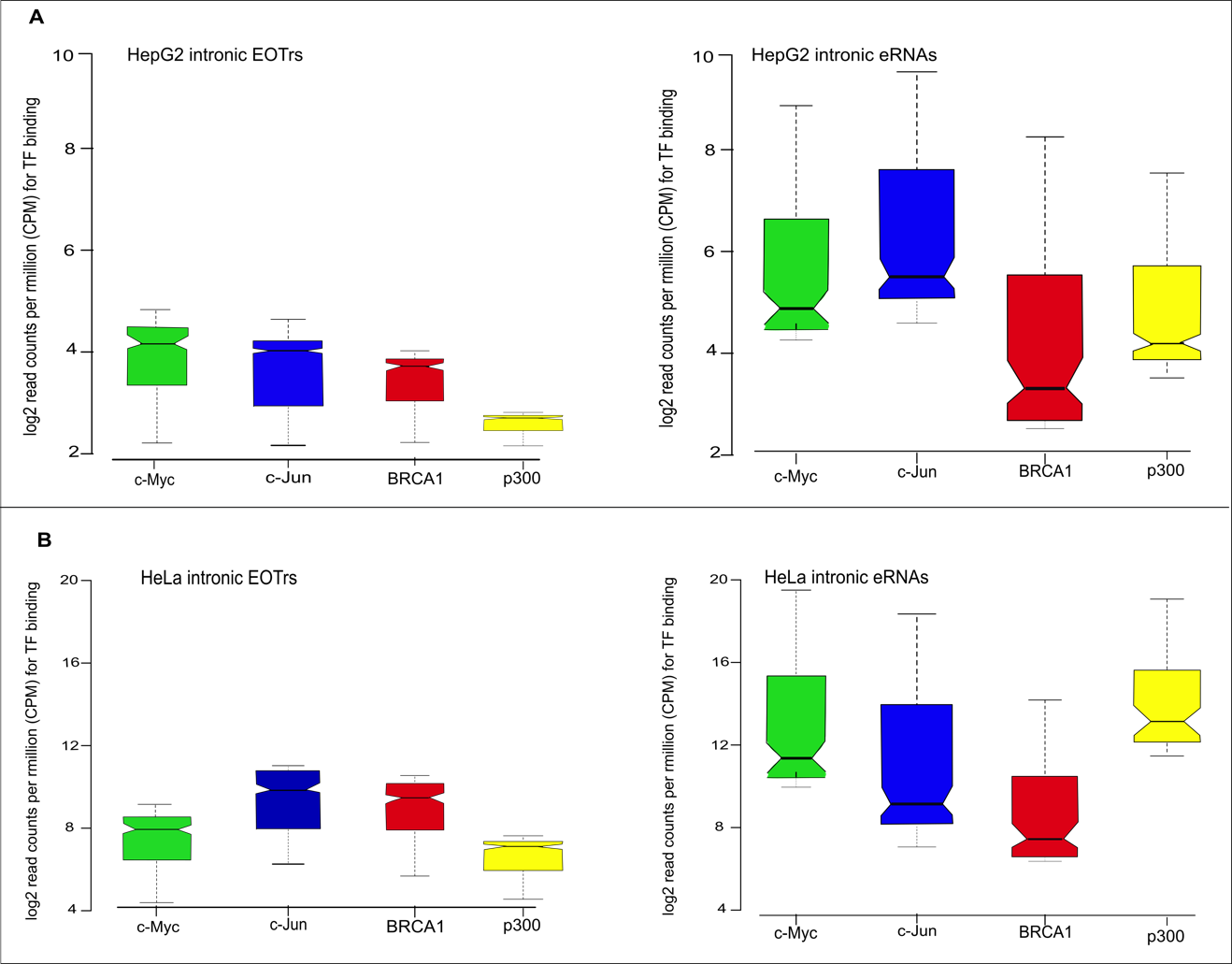


**Supplementary File 3 figure S3a.** Notched boxplots for enrichments of TFs (transcription factor) c-Myc, c-Jun, BRCA1 and p300; displayed are intronic enhancer domains intersected by EOTrs or eRNAs in HepG2 (**A**) and HeLa (**B**) cell lines. ChIP-seq signals for TF/DNA binding were monitored with p300 broad peaks as reference. Lower TF/DNA occupancy levels for regions intersected by EOTrs reflect the different outcomes of EOTr and eRNA-related transcription. Higher occupancy levels for the eRNA-intersected domains agreed with the STAP analysis and higher activities of the corresponding enhancers.


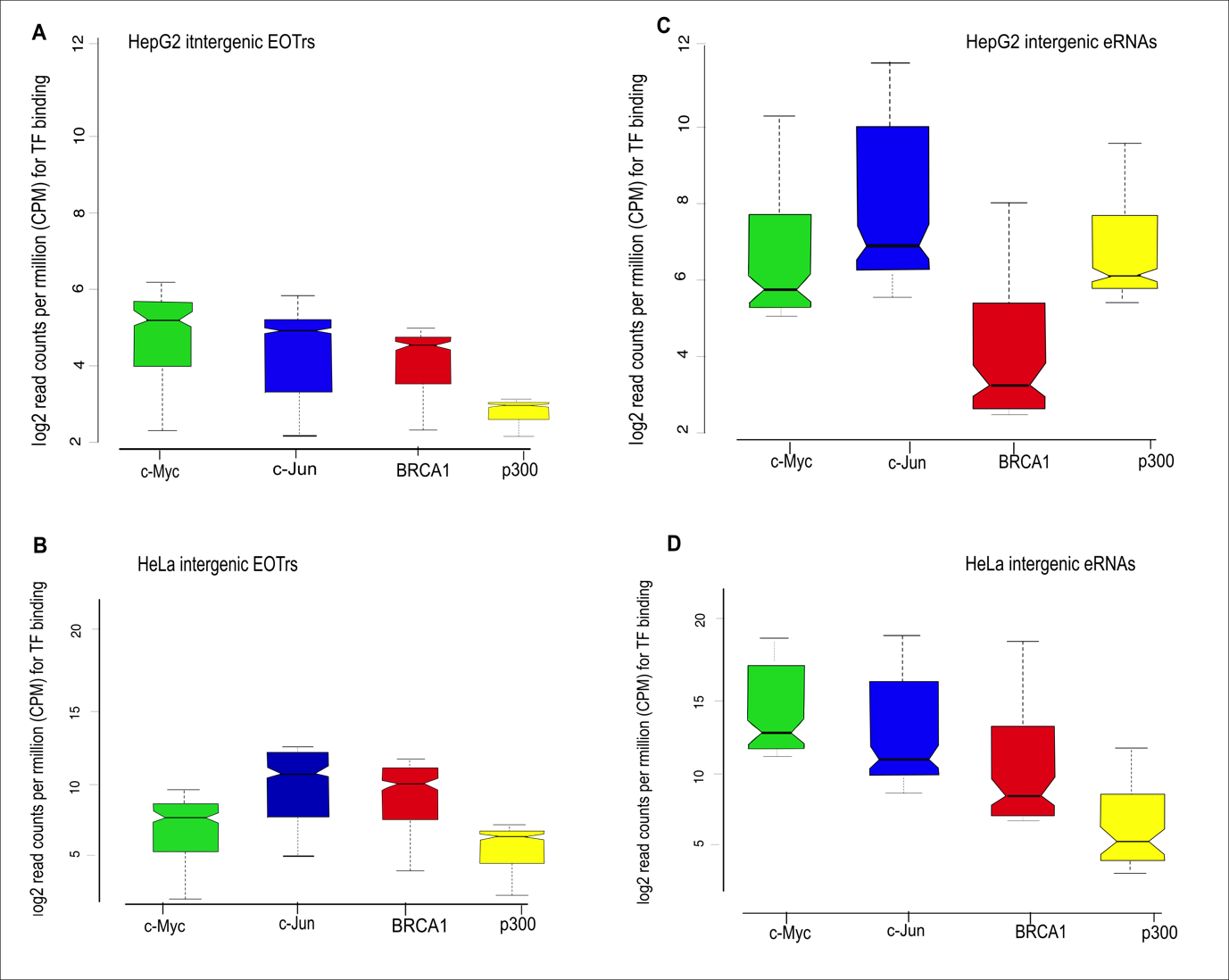


**Supplementary File 3 figure S3b.** Notched boxplots for enrichments of TFs (transcription factor) c-Myc, c-Jun, BRCA1 and p300; displayed are intergenic enhancer domains intersected by EOTrs or eRNAs in HepG2 (**A**) and HeLa (**B**) cell lines. ChIP-seq signals for TF/DNA binding were monitored with p300 broad peaks as reference. Lower TF/DNA occupancy levels for regions intersected by EOTrs reflect the different outcomes of EOTr and eRNA-related transcription. Higher occupancy levels for the eRNA-intersected domains agreed with the STAP analysis and higher activities of the corresponding enhancers.

**3.4 STAP/TRAP analysis to delineate relative transcription factor binding affinities for eRNA-intersected enhancer domains compared to genome-wide counterparts devoid of RNA expression**

As a further control for the analysis of TF-binding affinities in eRNA-intersected domains we compared relative affinities for TF-binding in eRNA-associated enhancers and domains devoid of RNA expression (the latter dataset excluded therefore EOTrs, eRNAs, H3K36me3+ and RNAPII+ regions). The results established unfavorable binding for sites lacking detectable levels of eRNA transcription (Supplementary File 3 table S4).

**Supplementary File 3 table S4.** STAP (sequence to affinity prediction) analysis for c-Myc, c-Jun and BRCA1 transcription factor/DNA binding for intergenic enhancer domains with enhancers devoid of RNA expression and eRNAs in HepG2 and HeLa cell lines: maxBindingWts=PWM scores, inFactorIntMat=favourable (>1)/unfavourable (<1) binding and expRatios=Pearson’s correlation (Materials and Methods).

| Cell line | Transcription factor | | maxBindingWts | | inFactorIntMat | | expRatios | |
| --- | --- | --- | --- | --- | --- | --- | --- | --- |
|  |  |  | eRNA | enhancers  devoid of RNA expression | eRNAs | enhancers devoid of RNA expression | eRNAs | enhancers devoid of RNA expression |
| HepG2 | 1. | c-Myc | 89.21 | 87.12 | 1.31 | 1.02 | 0.81 | 0.21 |
|  | 2. | c-Jun | 71.93 | 72.14 | 1.77 | 1.25 | 0.76 | 0.42 |
|  | 3. | BRCA1 | 80.72 | 79.86 | 1.92 | 1.01 | 0.66 | 0.20 |
|  | . |  |  |  |  |  |  |  |
| HeLa | 1. | c-Myc | 89.36 | 89.00 | 1.36 | 1.12 | 0.78 | 0.47 |
|  | 2. | c-Jun | 79.32 | 78.56 | 1.89 | 1.35 | 0.81 | 0.51 |
|  | 3. | BRCA1 | 83.29 | 81.22 | 1.35 | 1.03 | 0.59 | 0.37 |

These results revealed higher relative binding affinities for enhancer domains associated with eRNAs compared to domains devoid of RNA expression. The results of this analysis were in line with earlier findings that eRNA-intersected domains are connected to active enhancers (1,18,19)[.](#_ENREF_19)

**3.5. H3K9ac and H3K27ac enrichments for eRNA-intersected enhancer domains in HeLa and HepG2 cell lines**

eRNAs (enhancer RNAs) reside within enhancer domains of higher activity (1,18) as revealed by enrichments of H3K9ac and H3K27ac histone tail modifications. We compared occupancy levels for these histone tail modifications in EOTr and eRNA domains to illustrate actual differences of both transcript classes (20-22)[. As anticipated, higher occupancy levels were detected within eRNA-intersected domains compared to EOTr-associated enhancers (Supplementary File 3 figures S4a and b).](#_ENREF_22)


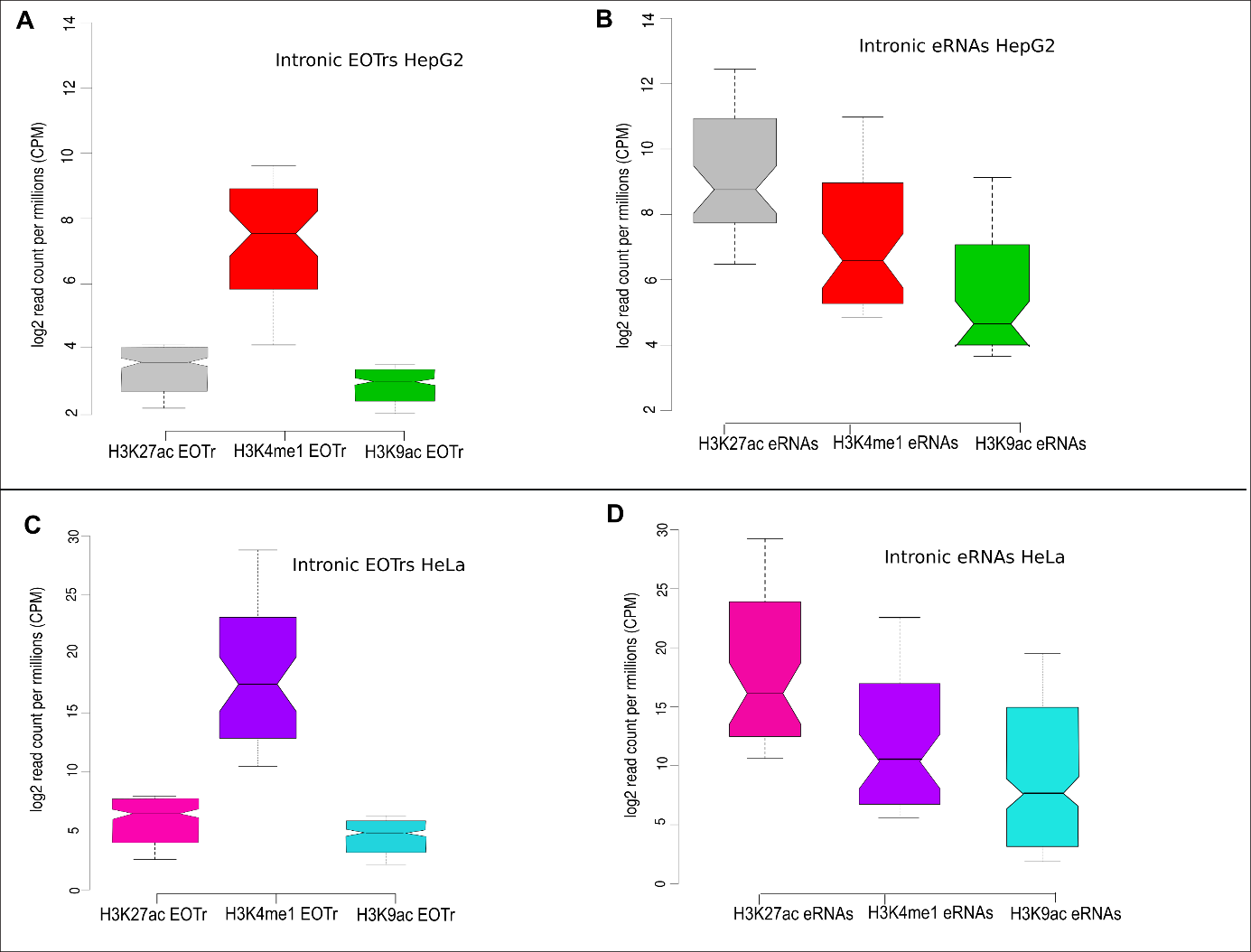


**Supplementary File 3 figure S4a.** Notched boxplots for H3K9ac and H3K27ac enrichments analyzed with the major p300 peak as reference for intronic enhancer domains associated with EOTrs and eRNAs, respectively. Results for intronic enhancer domains associated with EOTrs (**A**) and eRNAs (**B**) for HepG2 cells. The complementing analysis for HeLa cells is displayed in (**C**) and (**D**). H3K27ac and H3K9ac enrichments were lower for enhancers associated with EOTrs compared to eRNA-intersected domains.

**
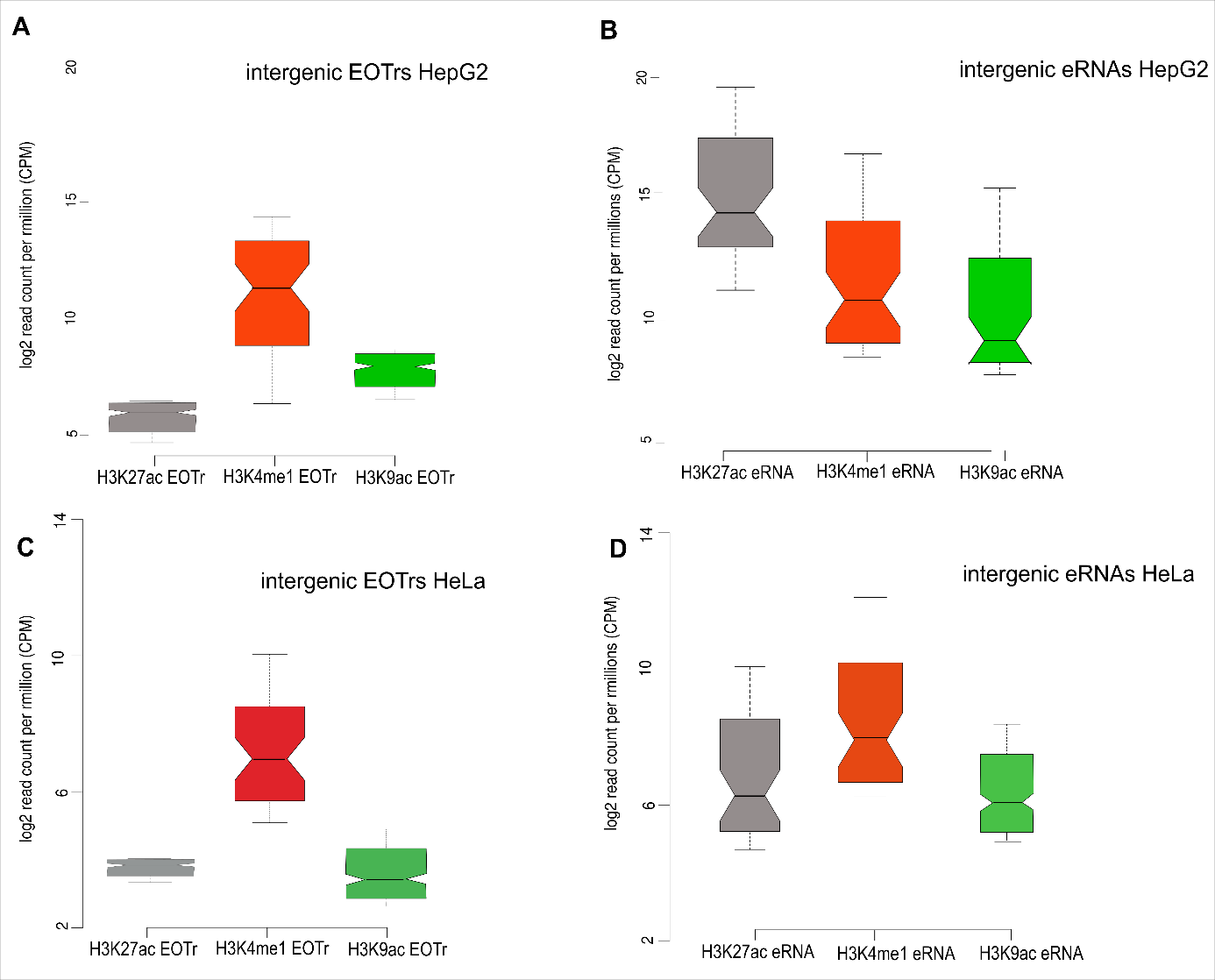
**

**Supplementary File 3 figure S4b.** Notched boxplots for H3K9ac and H3K27ac enrichments analyzed with the major p300 peak as reference for intergenic domains associated with EOTrs and eRNAs, respectively. Results for intergenic enhancer domains associated with EOTrs (**A**) and eRNAs (**B**) for HepG2 cells. The complementing analysis for HeLa cells is displayed in (**C**) and (**D**). H3K27ac and H3K9ac enrichments were lower for enhancers associated with EOTrs compared to eRNA-intersected domains.

**3.6 ChIA-PET and 5C analysis to derive interactomes for eRNA-intersected enhancer domains in HeLa and HepG2 cell lines**

ChIA-PET (Chromatin Interaction Analysis by Paired-End Tag Sequencing) and 5C analysis enabled the genome-wide characterization of chromatin looping interactions for eRNA datasets. The resulting interactomes were further analyzed for gene promoters interacting with eRNA-controlled enhancer domains. Higher enhancer activities, as identified in case of eRNA-coupled enhancer domains, were reflected in elevated expression levels of connected protein-coding RefSeq genes as demonstrated by the comparison to genes regulated by EOTr–associated enhancers (Supplementary File 3 figure S5). For ranking of RefSeq genes according their expression, we categorized the entire mRNA dataset in three groups (high, medium and low - high (HepG2 CPM ≥ 7.05 ≤ 12.6, HeLa ≥ 8.03 ≤ 14.1), medium (HepG2 CPM> 4.02 ≤ 10.7, HeLa >5.01≤ 11.2) and low (HepG2 CPM≤ 0.80-1.2, HeLa ≤ 0.60-2.3 ) *via* K-means (23)[. RefSeq genes interacting with eRNA-coupled enhancers ranked among the group of highly expressed genes; however, their counterparts linked to EOTr-associated enhancers were only of low expression (Supplementary File 3 figure S5).](#_ENREF_25)


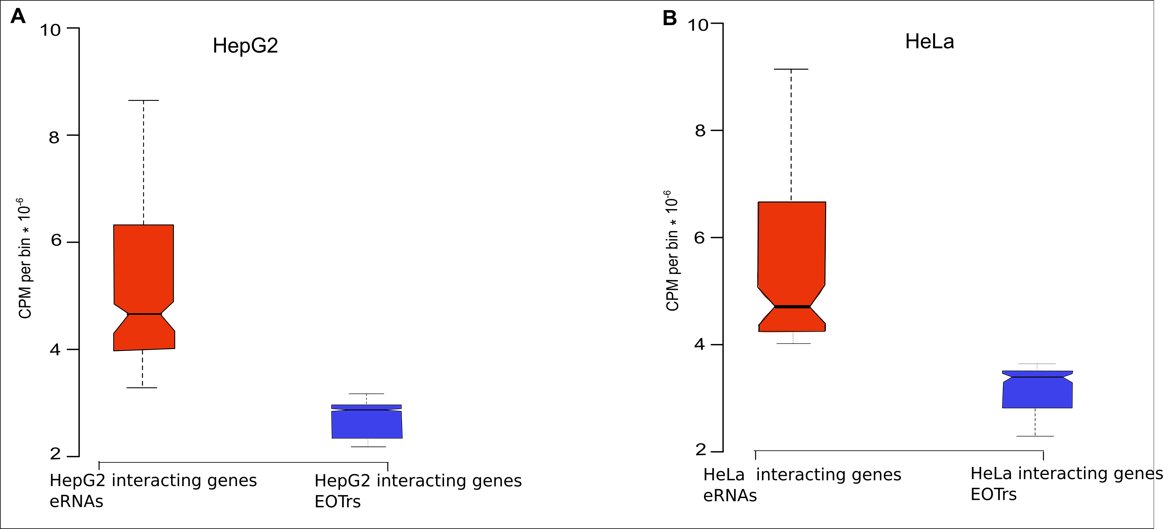


**Supplementary File 3 figure S5.** Gene expression for RefSeq genes associated with EOTrs and eRNA regulated enhancers in (**A**) HepG2 and (**B**) HeLa cell lines. This analysis demonstrated the impact of EOTr-mediated regulation on gene expression. Values represent log2 CPM (per bin) = number of reads per bin / number of mapped reads (in millions) to account for feature length.

**References:**

1. Kim, T.K., Hemberg, M., Gray, J.M., Costa, A.M., Bear, D.M., Wu, J., Harmin, D.A., Laptewicz, M., Barbara-Haley, K., Kuersten, S. *et al.* (2010) Widespread transcription at neuronal activity-regulated enhancers. *Nature*, **465**, 182-U165.

2. Andersson, R., Gebhard, C., Miguel-Escalada, I., Hoof, I., Bornholdt, J., Boyd, M., Chen, Y., Zhao, X., Schmidl, C., Suzuki, T. *et al.* (2014) An atlas of active enhancers across human cell types and tissues. *Nature*, **507**, 455-461.

3. De Santa, F., Barozzi, I., Mietton, F., Ghisletti, S., Polletti, S., Tusi, B.K., Muller, H., Ragoussis, J., Wei, C.L. and Natoli, G. (2010) A Large Fraction of Extragenic RNA Pol II Transcription Sites Overlap Enhancers. *Plos Biol*, **8**.

4. Melgar, M.F., Collins, F.S. and Sethupathy, P. (2011) Discovery of active enhancers through bidirectional expression of short transcripts. *Genome Biol*, **12**.

5. Blinka S, Reimer MH Jr, Pulakanti K, Pinello L, Yuan GC, Rao S. Identification of Transcribed Enhancers by Genome-Wide Chromatin Immunoprecipitation Sequencing. *Methods Mol Biol.* 2017;1468:91-109. doi: 10.1007/978-1-4939-4035-6_8.

6. Melo, C.A., Drost, J., Wijchers, P.J., van de Werken, H., de Wit, E., Oude Vrielink, J.A., Elkon, R., Melo, S.A., Leveille, N., Kalluri, R. *et al.* (2013) eRNAs are required for p53-dependent enhancer activity and gene transcription. *Molecular cell*, **49**, 524-535

7. Cheng, J.H., Pan, D.Z., Tsai, Z.T. and Tsai, H.K. (2015) Genome-wide analysis of enhancer RNA in gene regulation across 12 mouse tissues. *Scientific reports*, **5**, 12648.

8. Kim, T.K. and Shiekhattar, R. (2015) Architectural and Functional Commonalities between Enhancers and Promoters. *Cell*, **162**, 948-959.

9. Kim, T.K., Hemberg, M. and Gray, J.M. (2015) Enhancer RNAs: a class of long noncoding RNAs synthesized at enhancers. *Cold Spring Harb Perspect Biol*, **7**, a018622.

10. Robinson, M.D., McCarthy, D.J. and Smyth, G.K. (2010) edgeR: a Bioconductor package for differential expression analysis of digital gene expression data. *Bioinformatics*, **26**, 139-140.

11. Ross-Innes, C.S., Stark, R., Teschendorff, A.E., Holmes, K.A., Ali, H.R., Dunning, M.J., Brown, G.D., Gojis, O., Ellis, I.O., Green, A.R. *et al.* (2012) Differential oestrogen receptor binding is associated with clinical outcome in breast cancer. *Nature*, **481**, 389-U177.

12. He, X., Chen, C.C., Hong, F., Fang, F., Sinha, S., Ng, H.H. and Zhong, S. (2009) A biophysical model for analysis of transcription factor interaction and binding site arrangement from genome-wide binding data. *PloS one*, **4**, e8155.

13. Thomas-Chollier, M., Hufton, A., Heinig, M., O'Keeffe, S., Masri, N.E., Roider, H.G., Manke, T. and Vingron, M. (2011) Transcription factor binding predictions using TRAP for the analysis of ChIP-seq data and regulatory SNPs. *Nature protocols*, **6**, 1860-1869.

14. Shearwin, K.E., Callen, B.P. and Egan, J.B. (2005) Transcriptional interference--a crash course. *Trends Genet*, **21**, 339-345.

15. Pande, A., Brosius, J., Makalowska, I., Makalowski, W. and Raabe, C.A. (2018) Transcriptional interference by small transcripts in proximal promoter regions. *Nucleic Acids Res*, **46**, 1069-1088.

16. Hartzog, G.A. and Martens, J.A. (2009) ncRNA transcription makes its mark. *EMBO J*, **28**, 1679-1680.

17. Erokhin, M., Davydova, A., Parshikov, A., Studitsky, V.M., Georgiev, P. and Chetverina, D. (2013) Transcription through enhancers suppresses their activity in Drosophila. *Epigenet Chromatin*, **6**.

18. Liu, F. (2017) Enhancer-derived RNA: A Primer. *Genom Proteom Bioinf*, **15**, 196-200.

19. Mikhaylichenko, O., Bondarenko, V., Harnett, D., Schor, I.E., Males, M., Viales, R.R. and Furlong, E.E.M. (2018) The degree of enhancer or promoter activity is reflected by the levels and directionality of eRNA transcription. *Gene Dev*, **32**, 42-57.

20. Creyghton, M.P., Cheng, A.W., Welstead, G.G., Kooistra, T., Carey, B.W., Steine, E.J., Hanna, J., Lodato, M.A., Frampton, G.M., Sharp, P.A. *et al.* (2010) Histone H3K27ac separates active from poised enhancers and predicts developmental state. *Proc Natl Acad Sci U S A*, **107**, 21931-21936.

21. Karmodiya, K., Krebs, A.R., Oulad-Abdelghani, M., Kimura, H. and Tora, L. (2012) H3K9 and H3K14 acetylation co-occur at many gene regulatory elements, while H3K14ac marks a subset of inactive inducible promoters in mouse embryonic stem cells. *BMC genomics*, **13**, 424.

22. Calo, E. and Wysocka, J. (2013) Modification of enhancer chromatin: what, how, and why? *Mol Cell*, **49**, 825-837.

23. Hartigan, J.A. and Wong, M.A. (1979) Algorithm AS 136: A K-Means Clustering Algorithm. *Journal of the Royal Statistical Society. Series C (Applied Statistics)*, **28**, 100-108.
