# Supplementary material for: Enhancer occlusion transcripts regulate the activity of human enhancer domains via transcriptional interference: a computational perspective
Source: Nucleic Acids Res. 2020 Mar 5;48(7):3435–54. doi: 10.1093/nar/gkaa026 (PMC7144904; doi:10.1093/nar/gkaa026)
Supplement: gkaa026_Supplemental_Files [file gkaa026_supplemental_files.zip › Supplementary_file_Methods_NAR_01_03_2020.doc]

**Workflow to isolate EOTrs**

ENCODE long RNA deep sequencing datasets were screened for the identification non-protein coding transcripts (>200nt) devoid of known functions. Transcriptionally active genes, i.e., mRNA (messenger RNA) and npcRNAs (non-protein coding RNA), reside in domains of H3K4me3/H3K36me3 enrichment (1). ENCODE cDNA contigs were intersected with these domains to reduce the number of intronic degradation products within input data. Resulting datasets were further analyzed with CAGE clusters selected via HMMs to identify RNAs connected to bona fide promoter structures.

Eukaryotic enhancers are imbedded within domains of relative H3K4me1 enrichment (2,3); to ensure that our RNA collections overlap with human enhancer domains, only transcripts, which intersected regions of H3K4me1 enrichments were considered for further analysis. To restrict the input data to novel RNAs, we employed CEAS (Cis-regulatory Element Annotation System), which allowed the identification of transcripts for which no functions were assigned. CEAS conventions were retained and we arbitrarily defined transcripts located >2Kb upstream or 1Kb downstream from RefSeq protein coding genes as intergenic transcripts (4). For reconfirm the detection of non-protein coding transcripts, devoid of detectable open reading frames, RNA collections were finally investigated *via* CPAT (Coding Potential Assessment Tool) (5). Transcriptional interference (TI) acting on TFBSs (Transcription Factor Binding Sites) within enhancer domains is correlated with significantly reduced TF-(Transcription factor) binding affinities. For identification, relative TF-binding affinities for sites intersected by our transcript datasets were compared to control datasets devoid of H3K36me3 (EOTr-) *via* STAP analysis (Sequence To Affinity Prediction) (6). Transcripts, which intersected TFBSs of lower relative binding affinity entered our input datasets and are referenced as EOTrs (see main text).

**Supplementary methods figure S1:** Analyzed features for EOTr, non-EOTr and eRNA domains. Histone tail modifications have been all drawn on log 2 scale. For abbreviations see main text.

Enrichments for analyzed histone modifications indicating enhancer activity are depicted for EOTr (candidates) (left) in comparison to eRNA-intersected (right) domains **(A)**. Higher enhancer activities as revealed by H3K9ac (yellow) and H3K27ac (dark blue) enrichments are displayed for eRNA (right) compared to EOTr intersected domains (left). eRNA intersected domains, unlike the EOTr-associated enhancers (left), were devoid of H3K36m3 (light green) enrichments. Upstream regions of eRNAs (right) were devoid of detectable enrichments for H3K4me3 (light red) promoter marks compared to EOTrs (left).

A comparison of analyzed histone tail modifications for EOTr (candidates) and non-EOTr regions is depicted in **(B)**. Enrichments for H3K9ac (yellow) and H3K27ac (dark blue) for non-EOTr (EOTr-) domains (right) are denoted. The absence of EOTrs is correlated with increase in TF-binding affinities (red). Absence of EOTr expression is also reflected by the diminished H3K36me3 enrichments (light green) in EOTr- (right) compared to EOTr+ domains (left). EOTr- regions reflect therefore key features of eRNA intersected enhancer domains.

The comparison for enhancers intersected by EOTrs with cell line specific expression is illustrated in **(C)**. Similar to **(B)** the non-transcribed enhancer datasets lacked H3K36me3 (light green) enrichments (right) but display increased occupancies for histone tail modifications indicating enhancer activity: H3K9ac (yellow) and H3K27ac (dark blue). Similarly, increased ChIP-seq signals for TF-binding (red) were observed (right vs left).

A cartoon to display analyzed features for the investigation of enhancers intersected by EOTrs with allele-specific expression is depicted in **(D).** As in **(C)** H3K36me3 enrichments (light green) coincide with EOTr expression (left) compared to alleles devoid of EOTrs (right). Enrichments for H3K9ac (yellow) and H3K27ac (dark blue) are indicative of increased enhancer activity for alleles devoid of EOTrs and correlate with increased TF-binding (red) (right).

**Identification of EOTrs - Intersection of candidate transcripts with domains of H3K36me3/H3K4me3 and H3K4me1 enrichment**

**Bedtools**

intersectBed –a transcripts.bed –b H3K36me3.bed –f 0.8 –r

intersectBed –a transcripts.bed –b H3K4me1.bed –f 0.8 –r

bedtools intersect -wa -wb / -a transcripts.bed / -b H3K36me3.bed H3K4me3.bed / -sorted / -filenames

**CEAS for enrichment of transcripts devoid of known function**

**CEAS code**

ceas --name=transcript_regions_ceas --pf-res=20 --gn-groups=top10.txt,bottom10.txt --gn-group-names='Top 10%,Bottom 10%' -g ./hg19/refGene -b hg19_ceas_pval1e-5_peaks.bed –o annotation.png

**Calculation of RNA expression via edgeR**

**edgeR code:**

cpm <- function(y)

UseMethod("cpm")

cpm.DGEList <- function(y, normalized.lib.sizes=TRUE, log=FALSE, prior.count=2)

\#	Counts per million for a DGEList

{	lib.size <- y$BAM$lib.size

if(normalized.lib.sizes) lib.size <- lib.size*y$samples$norm.factors

cpm.default(y$counts,lib.size=lib.size,log=log,prior.count=prior.count)}

cpm.default <- function(y, lib.size=NULL, log=FALSE, prior.count=2)

\#	Counts per million for a matrix

{#	Check y

y <- as.matrix(y)

if (any(dim(y)==0L)) {

return(y)	}

\#	Check lib.size

if(is.null(lib.size)) lib.size <- colSums(y)

if(!is.double(lib.size)) {

if(!is.numeric(lib.size)) stop("lib.size must be numeric")

storage.mode(lib.size) <- "double"}

lib.size <- makeCompressedMatrix(lib.size, dim(y), byrow=TRUE)

check.range <- suppressWarnings(range(lib.size))

if (any(is.na(check.range)) || check.range[1] <= 0) {

stop ("library sizes should be finite and non-negative")	}

\#	Calculating in C++ for max efficiency

if(log) {prior.count <- .compressPrior(y, prior.count)

out <- .Call(.cxx_calculate_cpm_log, y, lib.size, prior.count)

} else {	out <- .Call(.cxx_calculate_cpm_raw, y, lib.size)	}

\#	Cleaning up

dimnames(out) <- dimnames(y)

out}

**List edgeR**

\## S3 method for class 'DGEList'

cpm (y, normalized.lib.sizes = TRUE, log = FALSE, prior.count = 2) dispersion = 0.05, offset = NULL, weights = NULL, ...)

**PlotBCV**:

plotBCV(y, xlab="Average log CPM", ylab="Biological coefficient of variation", pch=16, cex=0.2, col.common="red", col.trend="blue", col.tagwise="black")

**Extract Difference in group of genes**

exactTest <- function(object, pair=1:2, dispersion="auto", rejection.region="doubletail", big.count=900, prior.count=0.125)

\#	Calculates exact p-values for the differential expression levels of tags in the two groups being compared.

{#	Check input if(!is(object,"DGEList")) stop("Currently only supports DGEList objects as the object argument.")

if(length(pair)!=2) stop("Pair must be of length 2.") rejection.region <- match.arg(rejection.region,c("doubletail","deviance","smallp"))

\#	Get group names

group <- as.factor(object$samples$group) levs.group <- levels(group)

if(is.numeric(pair))

pair <- levs.group[pair]	else

pair <- as.character(pair)

if(!all(pair %in% levs.group)) stop("At least one element of given pair is not a group.\n Groups are: ", paste(levs.group, collapse=" "))

\#	Get dispersion vector

if(is.null(dispersion)) dispersion <- "auto"

if(is.character(dispersion)) { dispersion <- match.arg(dispersion,c("auto","common","trended","tagwise"))

dispersion <- switch(dispersion, "common"=object$common.dispersion, "trended"=object$trended.dispersion,

"tagwise"=object$tagwise.dispersion, "auto"=getDispersion(object) )

if(is.null(dispersion)) stop("specified dispersion not found in object")

if(is.na(dispersion[1])) stop("dispersion is NA")	}

ldisp <- length(dispersion) ntags <- nrow(object$counts)	if(ldisp!=1 && ldisp!=ntags) stop("Dispersion provided by user must have length either 1 or the number of tags in the DGEList object.")	if(ldisp==1) dispersion <- rep(dispersion,ntags)

\#	Reduce to two groups

group <- as.character(group)

j <- group %in% pair

y <- object$counts[,j,drop=FALSE]

lib.size <- object$samples$lib.size[j]

norm.factors <- object$samples$norm.factors[j]

group <- group[j]

if(is.null(rownames(y))) rownames(y) <- paste("tag",1:ntags,sep=".")

\#	Normalized library sizes

lib.size <- lib.size * norm.factors

offset <- log(lib.size) lib.size.average <- exp(mean(offset))

\#	logFC

prior.count <- prior.count*lib.size/mean(lib.size)

offset.aug <- log(lib.size+2*prior.count)

j1 <- group==pair[1]

n1 <- sum(j1)

if(n1==0) stop("No libraries for",pair[1])

y1 <- y[,j1,drop=FALSE]

abundance1 <- mglmOneGroup(y1+matrix(prior.count[j1],ntags,n1,byrow=TRUE),offset=offset.aug[j1],dispersion=dispersion)

j2 <- group==pair[2]

n2 <- sum(j2)

if(n1==0) stop("No libraries for",pair[2])

y2 <- y[,j2,drop=FALSE]

abundance2 <- mglmOneGroup(y2+matrix(prior.count[j2],ntags,n2,byrow=TRUE),offset=offset.aug[j2],dispersion=dispersion)

logFC <- (abundance2-abundance1)/log(2)

\#	Equalize library sizes

abundance <- mglmOneGroup(y,dispersion=dispersion,offset=offset)

e <- exp(abundance)

input.mean <- matrix(e,ntags,n1)

output.mean <- input.mean*lib.size.average

input.mean <- t(t(input.mean)*lib.size[j1])

y1 <- q2qnbinom(y1,input.mean=input.mean,output.mean=output.mean,dispersion=dispersion)

input.mean <- matrix(e,ntags,n2)

output.mean <- input.mean*lib.size.average

input.mean <- t(t(input.mean)*lib.size[j2])

y2 <- q2qnbinom(y2,input.mean=input.mean,output.mean=output.mean,dispersion=dispersion)

exact.pvals <- switch(rejection.region,

doubletail=exactTestDoubleTail(y1,y2,dispersion=dispersion,big.count=big.count),

deviance=exactTestByDeviance(y1,y2,dispersion=dispersion),

smallp=exactTestBySmallP(y1,y2,dispersion=dispersion)	)

AveLogCPM <- object$AveLogCPM

if(is.null(AveLogCPM)) AveLogCPM <- aveLogCPM(object)

de.out <- data.frame(logFC=logFC, logCPM=AveLogCPM, PValue=exact.pvals)

rn <- rownames(object$counts)

if(!is.null(rn)) rownames(de.out) <- make.unique(rn)

new("DGEExact",list(table=de.out, comparison=pair, genes=object$genes)) }

**Coding potential of long transcripts**

**CPAT code**

\# use BED file as input. '-r' is required

$ cpat.py -r /database/hg19.fa -g Human_test_coding_mRNA_hg19.bed -d ../dat/Human_logitModel.RData -x ../dat/Human_Hexamer.tsv -o output1

$ cpat.py -r /database/hg19.fa -g EOTr.bed -d ../dat/Human_logitModel.RData -x ../dat/Human_Hexamer.tsv -o output1

make_hexamer_tab.py -c Human_coding_transcripts_CDS.fa -n EOTr_RNA.fa >Human_Hexamer.tsv

make_logitModel.py -x Human_Hexamer.tsv -c Human_coding_transcripts_mRNA.fa -n EOTr_RNA.fa -o Human

**ChIP-seq for histone tail modifications and transcription factors - peak calling**

**Macs2**

macs2 callpeak -t c-myc.bam -c Control.bam -f BAM -g hs -n peaks -B -q 0.01 -m 10 100

**DiffBind Code:**

1. Construction of a new DBA object

dba(DBA,mask, minOverlap=2, sampleSheet="EOTr.csv", config=data.frame(RunParallel=TRUE, reportInit="DBA", DataType=DBA_DATA_GRANGES, AnalysisMethod=DBA_edgeR, minQCth=15, fragmentSize=125, bCorPlot=FALSE, th=0.05, bUsePval=FALSE), peakCaller="raw", peakFormat, scoreCol, bLowerScoreBetter, filter, skipLines=0, bAddCallerConsensus=FALSE, bRemoveM=TRUE, bRemoveRandom=TRUE, bSummarizedExperiment=FALSE, bCorPlot, attributes, dir)

2. differential binding affinity analysis

dba.analyze(DBA, method=DBA$config$AnalysisMethod, bSubControl=TRUE, bFullLibrarySize=TRUE, bTagwise=TRUE,

filter=0, filterFun=max, bCorPlot=DBA$config$bCorPlot, bReduceObjects=TRUE,

bParallel=DBA$config$RunParallel)

3. contrasts for differential binding affinity analysis

dba.contrast(DBA, group1, group2=!group1, name1="BRCA1", name2="c-MYc", name3="c-Jun",

minMembers=3, block, bNot=FALSE,

categories=c(DBA_TISSUE,DBA_FACTOR,DBA_CONDITION,DBA_TREATMENT))

4. Counts reads in binding site intervals

dba.count(DBA, peaks, minOverlap=2, score=DBA_SCORE_TMM_MINUS_FULL, bLog=FALSE,

fragmentSize=DBA$config$fragmentSize,

summits, filter=0, bRemoveDuplicates=FALSE, bScaleControl=TRUE,

mapQCth=DBA$config$mapQCth,

filterFun=max, bCorPlot=DBA$config$bCorPlot, bUseSummarizeOverlaps=FALSE, readFormat=DBA_READS_DEFAULT, bParallel=DBA$config$RunParallel)

5. subset of peaksets

dba.mask(DBA, attribute, value, combine='or', mask, merge='or', bApply=FALSE, peakset, minValue=-1)

6. report for a differential binding affinity analysis

dba.report(DBA, contrast, method=DBA$config$AnalysisMethod, th=DBA$config$th, bUsePval=DBA$config$bUsePval,

fold=0, bNormalized=TRUE, bFlip=FALSE, precision, bCalled=FALSE, bCounts=FALSE, bCalledDetail=FALSE,

bDB, bNotDB, bAll=TRUE, bGain=FALSE, bLoss=FALSE, file,initString=DBA$config$reportInit,ext='EOTr.csv',DataType=DBA$config$DataType)

7. Boxplots for read count distributions within differentially bound sites

dba.plotBox(DBA, contrast=1, method=DBA$config$AnalysisMethod, th=DBA$config$th, bUsePval=DBA$config$bUsePval,

bNormalized=TRUE, attribute=DBA_GROUP (EOTr_non-EOTr), bAll=FALSE, bAllIncreased=FALSE, bAllDecreased=FALSE,

bDB=TRUE, bDBIncreased=TRUE, bDBDecreased=TRUE, pvalMethod=wilcox.test, bReversePos=FALSE, attribOrder, vColors, varwidth=TRUE, notch=TRUE)

**Analysis of transcription factor binding and relative binding affinities**

**Pscan code**

pscan_chip -r EOTr.bed -g hg19 -M jaspar.wil -bg BG/HepG2.jaspar.bg

pscan_chip -r EOTr.bed -g hg19 -M jaspar.wil -bg BG/HeLa.jaspar.bg

**STAP code**

./seq2binding -s <EOTr.fa> -d <TFBS> -m <motifs> -io 3 –cc1 5 –dt all -et 10 –pm

**GRO-seq (global run-on-sequencing) data**

fastq-dump -I --split-files GSM2428726

fastq-dump -I --split-files GSE62046

homerTools trim -3 AAAAAAAA s_1_sequence.txt

FASTX (-q 10 -p 97)

Bowtie (-v 2 -k 3 -m 1 --best)

annotatePeaks.pl rb.distalPeaks.txt hg19 -size 4000 -hist 25 -d HepG2-GroSeq.bam -pc 1 > outputFile.txt

annotatePeaks.pl rb.distalPeaks.txt hg19 -size 4000 -hist 25 -d HeLa-GroSeq.bam -pc 1 > outputFile.txt

**Allele specific (AE) expression of EOTrs and accompanying activity of enhancer domains**

java -jar GenomeAnalysisTK.jar	-R reference.fasta	-T ASEReadCounter	-o EOTr_allele_name.csv	-I EOTr.bam	-sites sites.vcf

-U ALLOW_N_CIGAR_READS [-minDepth 10]	[--minMappingQuality 10]	[--minBaseQuality 2] [-drf DuplicateRead]

sprint prepare | [options] | reference_genome (hg19.fa) | bwa_path

sprint main | [options] | reference_genome(hg19.fa) | output_path | bwa_path | samtools_path

sprint_from_bam | [options] | aligned_reads(.bam) | reference_genome(hg19.fa) | output_path | samtools_path

**ChIA-PET analysis for HeLa cells**

JDK>=1.8(https://www.oracle.com/technetwork/java/javase/downloads/index.html) ,BWA(http://bio-bwa.sourceforge.net/), SAMtools(http://samtools.sourceforge.net/), BEDTools(https://bedtools.readthedocs.io/en/latest/), R(https://www.r-project.org/), R package grid(install.packages("grid")), R package xtable(install.packages("xtable")), R package RCircos (install.packages("RCircos"))

java -jar ChIA-PET.jar Necessary options: --mode: 1 for long read. --fastq1: path of read1 fastq file. --fastq2: path of read2 fastq file.

--linkerA: linker sequence, --linkerB: linker sequence, --minimum_linker_alignment_score: Specifies the allowed minimum alignment score.

--GENOME_INDEX: specifies the path of BWA index file --CHROM_SIZE_INFO: specifies the file that contains the length of each chromosome. --CYTOBAND_DATA: specifies the ideogram data used to plot intra-chromosomal peaks and interactions. --SPECIES: 1

Optional options:

--start_step: start with which step, 1: linker filtering; 2: mapping to genome; 3: removing redundancy; 4:

categorization of PETs; 5: peak calling; 6: interaction calling; 7: visualizing, default: 1

--output:default: ChIA-PET_Tool_V3/

output.

--prefix: default: out

--minimum_tag_length: 18

--maximum_tag_length: 1000

--minSecondBestScoreDiff: 3

--output_data_with_ambiguous_linker_info: 1

--thread: 1

--MAPPING_CUTOFF: 30

--MERGE_DISTANCE: 2

--SELF_LIGATION_CUFOFF: 8000

--EXTENSION_LENGTH: 500

--MIN_COVERAGE_FOR_PEAK: 5

--PEAK_MODE: 2

--MIN_DISTANCE_BETWEEN_PEAK: 500

--GENOME_COVERAGE_RATIO: 0.8

--PVALUE_CUTOFF_PEAK: 0.00001

--INPUT_ANCHOR_FILE: null

--PVALUE_CUTOFF_INTERACTION: 0.05

**5C analysis for HepG2 cells**

> hifive 5c-data [-h] (-B BAM BAM | -C COUNT) [-q] fragment output

> hifive 5c-normalize [-hg19] [-m 1] [-x 500] [-r EOTr.bed] [-o OUTPUT] [-q] [0.5]

> hifive 5c-[-r RE] [-g hg19] (-B hiC.bam | -C count) [-f 10] [-m 500] [-x 1000]

[-r EOTr.bed] (-o OUTPUT | -P PREFIX) [-q] [normalization options] bed

**Binning**

fivec.find_binning_fragment_corrections(max_iterations=1000,mindistance=50000,maxdistance=0, num_bins=[10, 10],

model=['len', 'gc'],parameters=['even', 'fixed-const'], usereads='cis',learning_threshold=1.0)

Sections A and B are adapted from (7).

**2.1. Section A:** Genome Structure Correction (8,9)

The Genome Structure Correction (GSC) measures physical distances between input data (i.e., the query) and annotated genomic features. As functional relationships between any two features statistically are defined on the basis of proximity/overlap/nearness to each other, observed deviations from expected distributions between any two features (e.g., H3K4me3 peaks overlapping 1Kb up- and downstream regions from the RefSeq annotated TSS, etc.) are indicative of associations (or non-associations) and hence their biological relevance. The GSC consists of four distinct statistical tests:

1. Relative distance Kolmogorov-Smirnov test

Reference and query intervals are investigated through their respective midpoints. A query point splits the distance between two reference points. The Null hypothesis suggests the distribution to be uniform. The real distribution, i.e., as displayed within analyzed data, is compared with the uniform *via* the Kolmogorov-Smirnov test.

2. The absolute distance test

The distance between a query point and the closest reference point is calculated. Results are compared with the distribution of distances obtained from permuted queries.

3. Jaccard test

The query-to-reference correspondence is calculated *via* the Jaccard measure (i.e., the ratio of powers of intersection and union). The results are compared with the distribution obtained from permuted queries.

4. Projection Bernoulli test

The probability (P) for a query to fall into a reference interval equals the part of the chromosome covered by the reference. If the query was independent of reference then the number of query points inside (or outside) the reference intervals follow a Bernoulli process, with the probability P and the number of trials that equals the number of query points. The outcome of this process (i.e., number of query points that overlap reference intervals) evaluates to the p-value for the (Bernoulli) Null hypothesis.

Supplementary table S1.A: Definition of active and poised enhancer state(s) in EOTrs and control regions.

| Active Enhancers | EOTrs | | Control regions | | Poised Enhancers | EOTrs | | Control regions | |
| --- | --- | --- | --- | --- | --- | --- | --- | --- | --- |
| (A) H3K4me1 | + | + | + | + | (A) H3K4me1 | + | + | + | + |
| (B) H3K9ac | + | + | + | + | (B) H3K27me3 | + | + | + | + |
| (C) H3K27ac | + | + | + | + | (C) H3K27ac | - | - | - | - |
| (D) p300 | + | + | + | + | (D) H3K9ac | - | - | - | - |

Combinations of peaks for histone tail modifications that were analyzed in EOTr and control (non-EOTr) regions for detection of active and poised enhancer or promoter states, respectively (Table B below). The Jaccard similarity, between vectors A, B, C and D is calculated as the combinatorial presence of active and poised states for EOTr and control regions.

Supplementary table S1.B: Analysis of active and poised promoter state(s)

Active and poised promoter state(s) calculated within 1Kb upstream regions (PPRs) for EOTrs and RefSeq genes (Table 2 main text).

| Active Promoters | Active EOTr promoters | | Control regions | | Poised Promoters | Poised EOTr promoters | | Control regions | |
| --- | --- | --- | --- | --- | --- | --- | --- | --- | --- |
| (A) H3K4me3 | + | + | + | + | (A) H3K4me3 | + | + | + | + |
| (B) H3K4me2 | + | + | + | + | (B) H3K27me3 | + | + | + | + |
| (C) H3K27ac | + | + | + | + | (C) H3K27ac | - | - | - | - |
| (D) RNAPII | + | + | + | + | (D) RNAPII | - | - | - | - |

**2.2. Section B: Supplementary Methods**

**Statistical Analysis**

**GSC (Genome Structure Correction)** (8,9)

The GenometriCorr R (Subsampling Methods for Genomic Inference) package was used to analyse the significance of overlaps between datasets. BED regions were used for the identification of active and poised enhancer/promoter states. Any detectable difference accounts for features (e.g., enrichments of histone tail modification, etc.) that are specifically associated with EOTr regions, compared to control data (i.e., the same feature and its overlap with control regions).

Jaccard index and GSC

a. The overlap of histone tail modifications within BED (Browser Extensible Data) regions representing active (H3K4me3+H3K4me2+H3K27ac+RNAPII-H3K27me3) and poised (H3K4me3+H3K27me3+RNAPII - H3K27ac) promoter states for EOTrs and RefSeq genes was calculated within 1Kb upstream region from the major CAGE (Cap Analysis of Gene Expression) peak. Results were compared to 1Kb downstream BED regions of EOTrs for the same combinations of histone tail modifications. Features representing poised and active stares are displayed separately within Supplementary 2.

b. The overlap of BED regions comprised active (H3K4me1+H3K9ac+H3K27ac+p300) and poised (H3K4me1+H3K27me3-H3K27ac) enhancer states within EOTr/eRNA loci. For EOTrs depending on the actual comparison non-EOTrs, i.e., as indicated, served as control. The association of combinations representing active and poised enhancer states in case of non-EOTrs were controlled with enrichments for the same histone tail modifications within EOTr BED regions. Features representing poised and active stares are displayed separately within Supplementary 2.

c. The analysis of overlapping BED regions for H3K36me3 histone tail modification within EOTr, i.e., 200bp upstream from the EOTr TTS (transcription termination site) and control regions (control regions: 200bps downstream from candidate EOTr 3’termini).

d. The interactome partners of EOTrs as identified by TargetFinder were scanned for enrichment of promoter, enhancer and insulator states. This was tested *via* GSC. H3K4me3 (promoter), H3K4me1 + p300 (enhancer) and CTCF (insulator) peak regions were scanned for interacting partners of intronic and intergenic enhancers regulated by EOTrs. Different histone states were compared against each other (i.e., promoter vs. enhancer, promoter vs. insulator and *vice versa*) to identify the dominant state for given interaction arm(s).

e. The overlapping BED regions of H3K27ac and H3K9ac peaks within EOTr and non-EOTr regions (control). Intersections were computed with starting peaks for analysed features within EOTr and non-EOTr regions while the union comprised of the starting peaks within the entire analysed regions and 1Kb upstream and downstream flanks.

**References**

1.	Kolasinska-Zwierz, P., Down, T., Latorre, I., Liu, T., Liu, X.S. and Ahringer, J. (2009) Differential chromatin marking of introns and expressed exons by H3K36me3. *Nat Genet*, 41, 376-381.

2.	Barski, A., Cuddapah, S., Cui, K., Roh, T.Y., Schones, D.E., Wang, Z., Wei, G., Chepelev, I. and Zhao, K. (2007) High-resolution profiling of histone methylations in the human genome. *Cell*, 129, 823-837.

3.	Heintzman, N.D., Stuart, R.K., Hon, G., Fu, Y., Ching, C.W., Hawkins, R.D., Barrera, L.O., Van Calcar, S., Qu, C., Ching, K.A. *et al.* (2007) Distinct and predictive chromatin signatures of transcriptional promoters and enhancers in the human genome. *Nat Genet*, 39, 311-318.

4.	Shin, H.J., Liu, T., Manrai, A.K. and Liu, X.S. (2009) CEAS: cis-regulatory element annotation system. *Bioinformatics*, 25, 2605-2606.

5.	Wang, L., Park, H.J., Dasari, S., Wang, S., Kocher, J.P. and Li, W. (2013) CPAT: Coding-Potential Assessment Tool using an alignment-free logistic regression model. *Nucleic Acids Res*, 41, e74.

6.	He, X., Chen, C.C., Hong, F., Fang, F., Sinha, S., Ng, H.H. and Zhong, S. (2009) A Biophysical Model for Analysis of Transcription Factor Interaction and Binding Site Arrangement from Genome-Wide Binding Data. *PloS one*, 4.

7.	Pande, A., Brosius, J., Makalowska, I., Makalowski, W. and Raabe, C.A. (2018) Transcriptional interference by small transcripts in proximal promoter regions. *Nucleic Acids Res*.

8.	Favorov, A., Mularoni, L., Cope, L.M., Medvedeva, Y., Mironov, A.A., Makeev, V.J. and Wheelan, S.J. (2012) Exploring Massive, Genome Scale Datasets with the GenometriCorr Package. *Plos Comput Biol*, 8.

9.	Bickel, P.J., Boley, N., Brown, J.B., Huang, H.Y. and Zhang, N.R. (2010) Subsampling Methods for Genomic Inference. *Ann Appl Stat*, 4, 1660-1697.
