# Supplementary material for: Enhancer occlusion transcripts regulate the activity of human enhancer domains via transcriptional interference: a computational perspective
Source: Nucleic Acids Res. 2020 Mar 5;48(7):3435–54. doi: 10.1093/nar/gkaa026 (PMC7144904; doi:10.1093/nar/gkaa026)
Supplement: gkaa026_Supplemental_Files [file gkaa026_supplemental_files.zip › Supplement_File_1_NAR_01_03_2020.doc]

**Supplementary File 1 figure S1.** Key epigenetic features that were analysed via the GSC (Genome Structure Correction) for intronic and intergenic EOTr (candidate) regions, enrichments for histone tail modifications indicative of promoter (H3K4me3) or enhancer (H3K4me1) states and active transcription (H3K36me3) were analysed as depicted. For calculation of Jaccard indices via the GSC we considered the number of starting peaks within 1Kb and 200bp flanking regions surrounding the TSS (Transcription Start Site) or TTS (Transcription Termination Site), respectively, as intersection. The union is represented by the number of analysed features within regions as displayed above, i.e., 2Kb in case of H3K4me3 promoter marks and 400bp for the analysis of H3K36me3 histone tail modifications near the candidate TTS. Figure not drawn to scale.

**Supplementary File 1 figure S2.** CPAT (Coding-Potential Assessment Tool) analysis of EOTr candidates in comparison to human RefSeq (hg19) CDS (Coding Sequence) exons. Results underscored that EOTrs are non-protein coding transcripts (Coding Potential >=0.364 indicates coding sequence, CP < 0.364 indicates non-protein coding sequence).

**Supplementary File 1 figure S3**. TRAP (Transcription factor Affinity Prediction) analysis of relative DNA-binding affinities for TFs (transcription factor): c-Myc, c-Jun and BRCA1 for intergenic non-EOTr (EOTr -) and EOTr (EOTr +) regions in HepG2 (left) and HeLa (right) cell lines (broad peaks). Each graph depicts relative binding affinities (y-axis) and sequence positions (x-axis) for the investigated TFs.

**Supplementary File 1 figure S4.** Notched boxplots for ChIP-seq signals of TFs (Transcription Factors) c-Myc, c-Jun, BRCA1 and p300. Depicted are intergenic EOTr (EOTrs +) and non-EOTr (EOTrs -) regions in HepG2 (**A**) and HeLa (**B**) cell lines. All signals of TF/DNA binding were monitored with p300 broad peaks as reference. Lower TF/DNA occupancy levels for regions intersected by EOTrs suggested the occlusion of TF-binding.

**Supplementary File 1 figure S5.** Combinations of histone tail modifications indicative of active promoters for intergenic EOTrs analysed within 1Kb upstream regions from the major CAGE (Cap Analysis of Gene Expression) cluster peak in HepG2 (**A**) and HeLa (**B**) cell lines*.* As a control, ChIP-seq footprints for the same modifications also were analysed within the corresponding 1Kb downstream regions from EOTr 3’ ends for HepG2 (**C**) and HeLa (**D**) cell lines.

**Supplementary File 1 figure S6**. Notched boxplots for H3K9ac and H3K27ac enrichments for EOTr and non-EOTr intergenic regions. Signals were analysed with p300 broad peaks as reference. Results for intergenic EOTr (EOTr +) and non-EOTr (EOTr -) regions for HepG2 (**A**) and HeLa (**B**) cell lines are displayed. Occupancy levels of H3K27ac and to lesser extent H3K9ac were lower for enhancer domains associated with EOTrs compared to non-EOTr regions.

**Supplementary File 1 section 1.1 EOTrs and enhancer activities: analysis across cell lines**

Enhancer activities were also analyzed for the same sites across cell lines. Only loci with cell line specific EOTr expression qualified as input data (i.e., either HepG2 or HeLa, multiple testing correction H3K27ac: HepG2→HeLa n=1103 and HeLa→HepG2 n=1883 and p-value corrected for multiple testing (q) HepG2→HeLa, q=2.9×10^–1^ and HeLa→HepG2, q=2.4×10^–3^ figure S7). Only domains associated with H3K4me1 enrichments in HeLa and HepG2 entered the analysis to ensure that H3K9ac and H3K27ac enrichments reflected activities of bona fide enhancers. This procedure allowed the investigation of effects related to EOTr expression for the same enhancer domains across cell lines. In the absence of EOTrs, the same enhancer domains were enriched for both histone tail modifications, indicating concomitantly higher activities.

**Supplementary File 1 figure S7.** Analysis of differential enrichments for H3K27ac (**A** and **B**) and H3K9ac (**C** and **D**) histone tail modifications for intergenic enhancer domains across HepG2 and HeLa cell lines; fold changes of ChIP-seq signals were calculated for enhancers that are intersected by EOTrs in HeLa but devoid of EOTrs in HepG2 cell lines (**A** and **C**). The complementing analysis for HepG2 cells is depicted in **B** and **D**. The results demonstrated consistently increased occupancy levels for either modification in the absence of EOTrs, suggesting that EOTr expression regulates the activity of corresponding enhancer domains. H3K9ac: HepG2→HeLa=1101 and HeLa→HepG2= 1942. Log base 2-fold change [L2FC] and p-value corrected for multiple testing (q) HepG2→HeLa, q=2.3×10^–1^ and HeLa→HepG2, q=3.7×10^–3^ .

.

**Supplementary File 1 figure S8.** Notched boxplots for RefSeq gene promoters (hg19) interacting with enhancers regulated *via* intergenic EOTrs in HepG2 (**A**) and HeLa (**B**) cell lines; chromatin landscapes revealed poised states for linked RefSeq gene promoters. H3K27me3 and H3K27ac signals were computed with H3K4me3 (broad peaks) as reference.

**Supplementary File 1 figure S9.** Analysis of epigenetic landscapes for RefSeq (hg19) gene promoters that interacted with intergenic enhancers regulated via EOTrs across cell lines: only gene promoters connected to enhancers that are regulated by cell line specific EOTrs entered this analysis. Results demonstrated the impact of EOTr expression on the corresponding promoter activities. Histone tail modifications indicating active and poised promoter states were computed with H3K4me3 as reference. Epigenetic footprints for promoters interacting with enhancers regulated by cell line specific EOTrs (EOTrs) are displayed for HepG2 (**A**) and HeLa (**B**) cell lines. Complementing analysis of promoter activities in the absence of EOTrs (EOTr-) for HeLa (**C**) and HepG2 **(D)** cell lines. HepG2→HeLa n=1016 and HeLa→HepG2 n=1736 Log base 2-fold change (L2FC) and p-value corrected for multiple testing (q) for H3K27ac- HepG2→HeLa, q=8.2×10^–3^ and HeLa→HepG2, q=6.2×10^–3^.

**Supplementary File 1 section 1.2 Analysis of H3K27me3 enrichments within EOTr loci**

Spurious transcription is limited by wrapping intergenic regions into heterochromatin. This shields DNA both from TF (transcription factor) and RNA polymerase binding. Across cell lines, we investigated whether enhancer domains associated with EOTrs were enriched in heterochromatin, which could be the reason for the lower enhancer activities and reduced affinities for TF-binding in case of EOTr-associated domains. No such associations or overlap of EOTrs with domains of heterochromatin environment were identified (Supplementary File 1 figure S10, HepG2→HeLa n=1179 and HeLa→HepG2 n=1989). Results revealed (almost) identical occupancy levels for H3K27me3 in enhancer domains independent of EOTr expression. Cell line specific spread of heterochromatin was not the cause of lower occupancy levels for TFs within enhancer domains associated with EOTrs. Hence, EOTr expression itself might be the major source of reduced relative TF/DNA binding affinities identified for the same enhancer domains across cell lines.

**Supplementary File 1 figure S10.** Notched boxplots to display enrichments of heterochromatin mark H3K27me3 for intronic EOTr regions within HepG2 (**A**) and HeLa (**B**) cell lines. Volcano plots for the analysis of differential H3K27me3 binding across cell lines for EOTr (EOTr+) regions compared to identical domains in the absence of EOTrs (EOTr-) for HepG2 (**C**) and HeLa (**D**) cell lines. Log base 2-fold change (L2FC) and p-value corrected for multiple testing (q) (HepG2→HeLa n= 1179, H3K27me3 q = 1.2 × 10^–9^, HeLa→HepG2 n=1989, H3K27me3 q = 1.2 × 10^–7^). H3K27me3 enrichments did not significantly alter across cell lines, suggesting that changes in heterochromatin organization are unlikely the reason for the lower activities of EOTr-associated enhancer domains.

**Supplementary File 1 figure S11.** Examples of transcriptional interference exerted via regulatory EOTr expression acting on intronic enhancer domains for the *MYTL1*, *MAPK3*, *THYN1* and *ARHGEF1* genes in HepG2 (**A** and **B**, **1^st^ frame**) and HeLa (**C** and **D**, **1^st^ frame**) cell lines, compared to the same enhancers in the absence of EOTrs for HeLa (**A** and **B**, **2^nd^ frame**) and HepG2 (**C** and **D**, **2^nd^ frame)** cell lines, respectively. Histone tail modifications indicating enhancer activities and transcription factor binding: H3K4me1 (blue), H3K9ac (green), and H3K27ac (brown and brown-red), p300 (pink) and c-Myc (red). Expression levels of EOTrs are displayed in black, genomic positions of analysed features and genes are indicated. Figures were drawn with the aid of the Human Epigenome Browser (http://epigenomegateway.wustl.edu/).

**References**

1.	Palazzo, A.F. and Lee, E.S. (2015) Non-coding RNA: what is functional and what is junk? *Front Genet*, **6**, 2.
