# Supplementary material for: Enhancer occlusion transcripts regulate the activity of human enhancer domains via transcriptional interference: a computational perspective
Source: Nucleic Acids Res. 2020 Mar 5;48(7):3435–54. doi: 10.1093/nar/gkaa026 (PMC7144904; doi:10.1093/nar/gkaa026)
Supplement: gkaa026_Supplemental_Files [file gkaa026_supplemental_files.zip › Supplement_File_2_NAR_01_03_2020.doc]

**Supplementary File 2 table S1.1.** CAGE (cap analysis of gene expression) cluster libraries in HepG2 and HeLa cell lines.

| **Cell line** | | **CAGE** |
| --- | --- | --- |
| **1** | HepG2 | https://www.ncbi.nlm.nih.gov/geo/query/acc.cgi?acc=GSE34448 |
| **2** | HeLa | https://www.ncbi.nlm.nih.gov/geo/query/acc.cgi?acc=GSE34448 |

**Supplementary File 2 table S1.2.** ChIP-seq libraries and ENCODE TFBS (Transcription Factor Binding Site) track for transcription factors in HepG2 and HeLa cell lines.

| **Cell line** | | **Transcription factor** | **Transcription factor ChIP-seq library** |
| --- | --- | --- | --- |
| **1** | HepG2  HeLa | c-Jun, c-Myc, BRCA1 and p300 | http://www.ncbi.nlm.nih.gov/geo/query/acc.cgi?acc=GSE31477  http://www.ncbi.nlm.nih.gov/geo/query/acc.cgi?acc=GSE32465  http://hgdownload.soe.ucsc.edu/goldenPath/hg19/encodeDCC/wgEncodeSydhTfbs  http://hgdownload.soe.ucsc.edu/goldenPath/hg19/encodeDCC/wgEncodeAwgTfbsUniform// |
| **2** |  |  |  |
| **3** | ENCODE ChIP-seq data track |  | https://genome.ucsc.edu/cgi-bin/hgTrackUi?g=wgEncodeTfBindingSuper |

**Supplementary File 2 table S1.3.** ChIP-seq libraries for histone tail modifications in HepG2 and HeLa cell lines.

| **Cell line** | | **Histone modification(s)** | **Histone modification ChIP-seq library** |
| --- | --- | --- | --- |
| **1** | HepG2  HeLa | H3K4me1, H3K4me3, H3K4me2, H3K27ac, RNAPII, H3K27me3, H3K79me2, H3K36me3 and CTCF | http://www.ncbi.nlm.nih.gov/geo/query/acc.cgi?acc=GSE29611 |
| **2** |  |  |  |

**Supplementary File 2 table S1.4.** Long RNA-Seq (from polyA+ total RNA) libraries for HepG2 and HeLa cell lines.

| **Cell line** | | **ENCODE Long (> 200nt) RNA seq polyA+ and polyA- libraries**  https://www.ncbi.nlm.nih.gov/geo/query/acc.cgi?acc=GSE26284 |
| --- | --- | --- |
| **1** | HepG2 |  |
| **2** | HeLa |  |

**Supplementary File 2 table S1.5.** 5C (Carbon Copy Chromosome Conformation Capture) and ChIA-PET (Chromatin Interaction Analysis by Paired-End Tag Sequencing) libraries for HepG2 and HeLa cell lines.

| HepG2 (5C) | http://www.ncbi.nlm.nih.gov/geo/query/acc.cgi?acc=GSM970288 |
| --- | --- |
| HeLa (ChIA-PET) | http://www.ncbi.nlm.nih.gov/geo/query/acc.cgi?acc=GSM970211 |

**Supplementary File 2 table S1.6.** GRO-seq libraries for HepG2 and HeLa cell lines.

| HepG2 GRO-seq | https://www.ncbi.nlm.nih.gov/geo/query/acc.cgi?acc=GSM2428726 |
| --- | --- |
| HeLa GRO-seq | https://www.ncbi.nlm.nih.gov/geo/query/acc.cgi?acc=GSE62046 |
| HepG2 RNAPII phosphorylated CTD | https://www.ncbi.nlm.nih.gov/geo/query/acc.cgi?acc= GSE32465 |
| HeLa RNAPII phosphorylated CTD | https://www.ncbi.nlm.nih.gov/geo/query/acc.cgi?acc= GSE2735 |

**Supplementary File 2 table S1a.** GSC analysis for H3K36me3 enrichments within EOTr-transcribed (200bp upstream from the EOTr TTS) and control regions (200bp downstream from the EOTr TTS) for intronic EOTr candidates in HepG2 cells.

| H3K36me3 within EOTr candidate regions  chromosomes=chr1, chr2, chr3, chr4, chr5, chr6, chr7,chr8, chr9, chr10, chr11,chr12, chr13, chr14 , chr15,chr16, chr17, chr18 , chr19,chr20, chr21, chr22 chrX,chrY  relative.distances.ks.p.value 1.101419e-06 0  relative.distances.ecdf.deviation.area 0.01215024  relative.distances.ecdf.area.correlation 0.11498763  intersection 45839  union 52689  jaccard.measure 0.87  projection.test.p.value 0  projection.test.lower.tail FALSE  relative.distances.ecdf.deviation.area.p.value "<0.01"  scaled.absolute.min.distance.sum.p.value "<0.01"  scaled.absolute.min.distance.sum.lower.tail TRUE  jaccard.measure.p.value "<0.01"  jaccard.measure.lower.tail FALSE | H3K36me3 in control regions  chromosomes=chr1, chr2, chr3, chr4, chr5, chr6, chr7,chr8, chr9, chr10, chr11,chr12, chr13, chr14 , chr15,chr16, chr17, chr18 , chr19,chr20, chr21, chr22 , chrX,chrY  relative.distances.ks.p.value 14.101126e-06 0  relative.distances.ecdf.deviation.area -1.02288904  relative.distances.ecdf.area.correlation -1.21234987  intersection 8250  union 52689  jaccard.measure 0.15  projection.test.p.value 0.09  projection.test.lower.tail TRUE  relative.distances.ecdf.deviation.area.p.value ">0.01"  scaled.absolute.min.distance.sum.p.value ">0.01"  scaled.absolute.min.distance.sum.lower.tail FALSE  jaccard.measure.p.value ">0.01"  jaccard.measure.lower.tail TRUE |
| --- | --- |

**Supplementary File 2 table S1b.** GSC analysis for H3K36me3 enrichments within EOTr-transcribed (200bp upstream from the EOTr TTS) and control regions (200bp downstream from the EOTr TTS) for intronic EOTr candidates in HeLa cells.

| H3K36me3 within EOTr candidate regions  chromosomes=chr1, chr2, chr3,chr4, chr5, chr6, chr7,chr8, chr9, chr10, chr11,chr12, chr13, chr14 , chr15,chr16, chr17, chr18 , chr19,chr20, chr21, chr22 , chrX, chrY    relative.distances.ks.p.value 0.1017651e-05 0  relative.distances.ecdf.deviation.area 0.0134902  relative.distances.ecdf.area.correlation 0.122675  intersection 36574  union 43702  jaccard.measure 0.83  projection.test.p.value 0  projection.test.lower.tail FALSE  relative.distances.ecdf.deviation.area.p.value "<0.01"  scaled.absolute.min.distance.sum.p.value "<0.01"  scaled.absolute.min.distance.sum.lower.tail TRUE  jaccard.measure.p.value "<0.01"  jaccard.measure.lower.tail FALSE | H3K36me3 within control regions  chromosomes=chr1, chr2, chr3,chr4, chr5, chr6, chr7,chr8, chr9, chr10, chr11,chr12, chr13, chr14 , chr15,chr16, chr17, chr18 , chr19,chr20, chr21, chr22 , chrX,chrY  relative.distances.ks.p.value 16.021426e-06 0  relative.distances.ecdf.deviation.area -3.01443026  relative.distances.ecdf.area.correlation -1.342156  intersection 6047  union 43702  jaccard.measure 0.13  projection.test.p.value 0.02  projection.test.lower.tail TRUE  relative.distances.ecdf.deviation.area.p.value ">0.01"  scaled.absolute.min.distance.sum.p.value ">0.01"  scaled.absolute.min.distance.sum.lower.tail FALSE  jaccard.measure.p.value ">0.01"  jaccard.measure.lower.tail TRUE |
| --- | --- |

**Supplementary File 2 table S1c.** GSC analysis for H3K36me3 enrichments within EOTr-transcribed (200bp upstream from the EOTr TTS) and control regions (200bp downstream from the EOTr TTS) for intergenic EOTr candidates in HepG2 cells.

| H3K36me3 within EOTr candidate regions  chromosomes=chr1, chr2, chr3,chr4, chr5, chr6, chr7,chr8, chr9, chr10, chr11,chr12, chr13, chr14 , chr15,chr16, chr17, chr18 , chr19,chr20, chr21, chr22 , chrX,chrY  relative.distances.ks.p.value 1.101321e-05 0  relative.distances.ecdf.deviation.area 0.0112142  relative.distances.ecdf.area.correlation 0.112654  intersection 119840  union 142677  jaccard.measure 0.84  projection.test.p.value 0.0  projection.test.lower.tail FALSE  relative.distances.ecdf.deviation.area.p.value "<0.01"  scaled.absolute.min.distance.sum.p.value "<0.01"  scaled.absolute.min.distance.sum.lower.tail TRUE  jaccard.measure.p.value "<0.01"  jaccard.measure.lower.tail FALSE | H3K36me3 within control regions  chromosomes=chr1, chr2, chr3,chr4, chr5, chr6, chr7,chr8, chr9, chr10, chr11,chr12, chr13, chr14 , chr15,chr16, chr17, chr18 , chr19,chr20, chr21, chr22 , chrX,chrY  relative.distances.ks.p.value 8.101329e-06 0  relative.distances.ecdf.deviation.area -1.036214235  relative.distances.ecdf.area.correlation -1.3234891  intersection 1812  union 142677  jaccard.measure 0.012  projection.test.p.value 0.2  projection.test.lower.tail TRUE  relative.distances.ecdf.deviation.area.p.value ">0.01"  scaled.absolute.min.distance.sum.p.value ">0.01" scaled.absolute.min.distance.sum.lower.tail FALSE  jaccard.measure.p.value ">0.01"  jaccard.measure.lower.tail TRUE |
| --- | --- |

**Supplementary File 2 table S1d.** GSC analysis for H3K36me3 enrichments within EOTr-transcribed (200bp upstream from the EOTr TTS) and control regions (200bp downstream from the EOTr TTS) for intergenic EOTr candidates in HeLa cells.

| H3K36me3 within EOTr candidate regions  chromosomes=chr1, chr2, chr3,chr4, chr5, chr6, chr7,chr8, chr9, chr10, chr11,chr12, chr13, chr14 , chr15,chr16, chr17, chr18 , chr19,chr20, chr21, chr22,chrX, chrY    relative.distances.ks.p.value 2.1121285e-05 0  relative.distances.ecdf.deviation.area 0.00536012  relative.distances.ecdf.area.correlation 0.11232851  intersection 27973  union 36397  jaccard.measure 0.76  projection.test.p.value 0  projection.test.lower.tail FALSE  relative.distances.ecdf.deviation.area.p.value <0.01  scaled.absolute.min.distance.sum.p.value <0.01  scaled.absolute.min.distance.sum.lower.tail TRUE  jaccard.measure.p.value <0.01  jaccard.measure.lower.tail FALSE | H3K36me3 within control regions  chromosomes=chr1, chr2, chr3,chr4, chr5, chr6, chr7,chr8, chr9, chr10, chr11,chr12, chr13, chr14 , chr15,chr16, chr17, chr18 , chr19,chr20, chr21, chr22 , chrX, chrY    relative.distances.ks.p.value 8.124801e-06 0  relative.distances.ecdf.deviation.area -0.01167812  relative.distances.ecdf.area.correlation -1.7890101  intersection 11663  union 36397  jaccard.measure 0.32  projection.test.p.value 0.001  projection.test.lower.tail TRUE  relative.distances.ecdf.deviation.area.p.value >0.01  scaled.absolute.min.distance.sum.p.value >0.01  scaled.absolute.min.distance.sum.lower.tail FALSE  jaccard.measure.p.value >0.01  jaccard.measure.lower.tail TRUE |
| --- | --- |

**Supplementary File 2 table S2a.** GSC analysis for H3K4me3 and H3K4me1 + p300 enrichments for intronic EOTr candidates in HepG2 cells.

| H3K4me1 + p300 within EOTr candidate regions  chromosomes.to.proceed = chr1, chr2, chr3,chr4, chr5, chr6, chr7,chr8, chr9, chr10, chr11,chr12, chr13, chr14 , chr15,chr16, chr17, chr18 , chr19,chr20, chr21, chr22 , chrX,chrY  relative.distances.ks.p.value 1.101329e-05 0  relative.distances.ecdf.deviation.area 0.0151123  relative.distances.ecdf.area.correlation 0.312587  intersection 15673  union 21345  jaccard.measure 0.73  projection.test.p.value 0  projection.test.lower.tail FALSE  relative.distances.ecdf.deviation.area.p.value <0.01  scaled.absolute.min.distance.sum.p.value <0.01  scaled.absolute.min.distance.sum.lower.tail TRUE  jaccard.measure.p.value <0.01  jaccard.measure.lower.tail FALSE | H3K4me3 within EOTr candidate regions  chromosomes.to.proceed = chr1, chr2, chr3,chr4, chr5, chr6, chr7,chr8, chr9, chr10, chr11,chr12, chr13, chr14 , chr15,chr16, chr17, chr18 , chr19,chr20, chr21, chr22 , chrX,chrY  relative.distances.ks.p.value 14.156297e-06 0  relative.distances.ecdf.deviation.area -1.02924237  relative.distances.ecdf.area.correlation -1.4456321  intersection 2371  union 21345  jaccard.measure 0.11  projection.test.p.value 0.04  projection.test.lower.tail TRUE  relative.distances.ecdf.deviation.area.p.value >0.01  scaled.absolute.min.distance.sum.p.value >0.01  scaled.absolute.min.distance.sum.lower.tail FALSE  jaccard.measure.p.value >0.01  jaccard.measure.lower.tail TRUE |
| --- | --- |

**Supplementary File 2 table S2b.** GSC analysis for H3K4me3 and H3K4me1 + p300 enrichments for intronic EOTr candidates in HeLa cells.

| H3K4me1 + p300 within EOTr candidate regions  chromosomes.to.proceed = chr1, chr2, chr3,chr4, chr5, chr6, chr7,chr8, chr9, chr10, chr11,chr12, chr13, chr14 , chr15,chr16, chr17, chr18 , chr19,chr20, chr21, chr22 , chrX,chrY  relative.distances.ks.p.value 2.10167e-05 0  relative.distances.ecdf.deviation.area 0.012478561  relative.distances.ecdf.area.correlation 0.213564  intersection 17324  union 23781  jaccard.measure 0.72  projection.test.p.value 0  projection.test.lower.tail FALSE  relative.distances.ecdf.deviation.area.p.value <0.01  scaled.absolute.min.distance.sum.p.value <0.01  scaled.absolute.min.distance.sum.lower.tail TRUE  jaccard.measure.p.value <0.01  jaccard.measure.lower.tail FALSE | H3K4me3 within EOTr candidate regions  chromosomes.to.proceed = chr1, chr2, chr3,chr4, chr5, chr6, chr7,chr8, chr9, chr10, chr11,chr12, chr13, chr14 , chr15,chr16, chr17, chr18 , chr19,chr20, chr21, chr22 , chrX,chrY  relative.distances.ks.p.value 7.156497e-06 0  relative.distances.ecdf.deviation.area -1.076423118  relative.distances.ecdf.area.correlation -1.7796521  intersection 2853  union 23781  jaccard.measure 0.12  projection.test.p.value 0.02  projection.test.lower.tail TRUE  relative.distances.ecdf.deviation.area.p.value >0.01  scaled.absolute.min.distance.sum.p.value >0.01  scaled.absolute.min.distance.sum.lower.tail FALSE  jaccard.measure.p.value >0.01  jaccard.measure.lower.tail TRUE |
| --- | --- |

**Supplementary File 2 table S2c.** GSC analysis for H3K4me3 and H3K4me1 + p300 enrichments for intergenic EOTr candidates in HepG2 cells.

| H3K4me1 + p300 within EOTr candidate regions  chromosomes= chr1, chr2, chr3,chr4, chr5, chr6, chr7,chr8, chr9, chr10, chr11,chr12, chr13, chr14 , chr15,chr16, chr17, chr18 , chr19,chr20, chr21, chr22 , chrX,chrY  relative.distances.ks.p.value 1.101129e-05 0  relative.distances.ecdf.deviation.area 0.0162223  relative.distances.ecdf.area.correlation 0.212587  intersection 25441  union 27654  jaccard.measure 0.92  projection.test.p.value 0  projection.test.lower.tail FALSE  relative.distances.ecdf.deviation.area.p.value <0.01  scaled.absolute.min.distance.sum.p.value <0.01  scaled.absolute.min.distance.sum.lower.tail TRUE  jaccard.measure.p.value <0.01  jaccard.measure.lower.tail FALSE | H3K4me3 within EOTr candidate regions  chromosomes.to.proceed = chr1, chr2, chr3,chr4, chr5, chr6, chr7,chr8, chr9, chr10, chr11,chr12, chr13, chr14 , chr15,chr16, chr17, chr18 , chr19,chr20, chr21, chr22 , chrX,chrY  relative.distances.ks.p.value 12.156297e-06 0  relative.distances.ecdf.deviation.area -1.01923247  relative.distances.ecdf.area.correlation -1.2436311  intersection 6360  union 27654  jaccard.measure 0.23  projection.test.p.value 0.02  projection.test.lower.tail TRUE  relative.distances.ecdf.deviation.area.p.value >0.01  scaled.absolute.min.distance.sum.p.value >0.01  scaled.absolute.min.distance.sum.lower.tail FALSE  jaccard.measure.p.value >0.01  jaccard.measure.lower.tail TRUE |
| --- | --- |

**Supplementary File 2 table S2d.** GSC analysis for H3K4me3 and H3K4me1 + p300 enrichments for intergenic EOTr candidates in HeLa cells.

| H3K4me1 + p300 within EOTr candidate regions  chromosomes.to.proceed = chr1, chr2, chr3,chr4, chr5, chr6, chr7,chr8, chr9, chr10, chr11,chr12, chr13, chr14 , chr15,chr16, chr17, chr18 , chr19,chr20, chr21, chr22 , chrX,chrY  relative.distances.ks.p.value 1.10167e-05 0  relative.distances.ecdf.deviation.area 0.011278521  relative.distances.ecdf.area.correlation 0.112654  intersection 25197  union 28312  jaccard.measure 0.89  projection.test.p.value 0  projection.test.lower.tail FALSE  relative.distances.ecdf.deviation.area.p.value <0.01  scaled.absolute.min.distance.sum.p.value <0.01  scaled.absolute.min.distance.sum.lower.tail TRUE  jaccard.measure.p.value <0.01  jaccard.measure.lower.tail FALSE | H3K4me3 within EOTr candidate regions  chromosomes.to.proceed = chr1, chr2, chr3,chr4, chr5, chr6, chr7,chr8, chr9, chr10, chr11,chr12, chr13, chr14 , chr15,chr16, chr17, chr18 , chr19,chr20, chr21, chr22 , chrX,chrY  relative.distances.ks.p.value 9.136497e-06 0  relative.distances.ecdf.deviation.area -1.026223418  relative.distances.ecdf.area.correlation -1.6793420  intersection 9059  union 28312  jaccard.measure 0.32  projection.test.p.value 0.01  projection.test.lower.tail TRUE  relative.distances.ecdf.deviation.area.p.value >0.01  scaled.absolute.min.distance.sum.p.value >0.01  scaled.absolute.min.distance.sum.lower.tail FALSE  jaccard.measure.p.value >0.01  jaccard.measure.lower.tail TRUE |
| --- | --- |

**Supplementary File 2 table S3.** BCV (Biological Coefficient of Variation) for long RNA-Seq replicates (pooled) within and across cell lines.

| **Cell line** | **BCV within sample** | **Across cell line** | **BCV** |
| --- | --- | --- | --- |
| HepG2 | 0.43 | HeLa | 0.47 |
| HeLa | 0.46 | HepG2 | 0.46 |

**Supplementary File 2 table S4.** Coding Potential Assessment Tool (CPAT) results for EOTrs and RefSeq CDS exons.

EOTr CPAT | stats

Total lines: 16107

Sum of lines: 1773.01404739716

Ari. Mean: 0.0789972396808571

Geo. Mean: 0.0157839580319542

Median: 0.0157583998480091

Mode: 1 (N=22)

Anti-Mode: 2.22144653496105e-13 (N=1)

Minimum: 2.22144653496105e-13

Maximum: 1

Variance: 0.0332286952048533

StdDev: 0.182287397273792

Coding potential of RefSeq annotated mRNAs

cat RefSeq

Total lines: 47251

Sum of lines: 36677.7025936008

Ari. Mean: 0.776231245764128

Geo. Mean: 0.405030720938695

Median: 0.999573383225558

Mode: 1 (N=3820)

Anti-Mode: 2.26544376749313e-13 (N=1)

Minimum: 2.26544376749313e-13

Maximum: 1

Variance: 0.141651908600363

StdDev: 0.376366720899129

**Supplementary File 2 table S5a.** GSC analysis for H3K4me3 enrichments within 1Kb up- and downstream regions surrounding the major CAGE cluster peak for intronic EOTr candidates in HepG2 cells.

| H3K4me3 within 1Kb upstream regions from major CAGE peak  chromosomes=chr1, chr2, chr3,chr4, chr5, chr6, chr7,chr8, chr9, chr10, chr11,chr12, chr13, chr14 , chr15,chr16, chr17, chr18 , chr19,chr20, chr21, chr22 ,chrX, chrY    relative.distances.ks.p.value 2.1021345e-05 0  relative.distances.ecdf.deviation.area 0.01127893  relative.distances.ecdf.area.correlation 0.212987  intersection 18291  union 23451  jaccard.measure 0.78  projection.test.p.value 0  projection.test.lower.tail FALSE  relative.distances.ecdf.deviation.area.p.value <0.01  scaled.absolute.min.distance.sum.p.value <0.01  scaled.absolute.min.distance.sum.lower.tail TRUE  jaccard.measure.p.value <0.01  jaccard.measure.lower.tail FALSE | H3K4me3 within 1Kb downstream regions from major CAGE peak  chromosomes=chr1, chr2, chr3,chr4, chr5, chr6, chr7,chr8, chr9, chr10, chr11,chr12, chr13, chr14 , chr15,chr16, chr17, chr18 , chr19,chr20, chr21, chr22 , chrX, chrY    relative.distances.ks.p.value 12.101343e-06 0  relative.distances.ecdf.deviation.area -1.01815026  relative.distances.ecdf.area.correlation -1.3245671  intersection 5393  union 23451  jaccard.measure 0.23  projection.test.p.value 0.10  projection.test.lower.tail TRUE  relative.distances.ecdf.deviation.area.p.value >0.01  scaled.absolute.min.distance.sum.p.value >0.01  scaled.absolute.min.distance.sum.lower.tail FALSE  jaccard.measure.p.value >0.01  jaccard.measure.lower.tail TRUE |
| --- | --- |

**Supplementary File 2 table S5b. GSC analysis for H3K4me3 enrichments within 1Kb up- and downstream regions surrounding the major CAGE cluster peak for intronic EOTr candidates in HeLa cells.**

| H3K4me3 within 1Kb upstream regions from major CAGE peak  chromosomes=chr1, chr2, chr3,chr4, chr5, chr6, chr7,chr8, chr9, chr10, chr11,chr12, chr13, chr14 , chr15,chr16, chr17, chr18 , chr19,chr20, chr21, chr22, chrX,chrY  relative.distances.ks.p.value 3.101329e-06 0  relative.distances.ecdf.deviation.area 0.01673011  relative.distances.ecdf.area.correlation 0.234752  intersection 13232  union 18864  jaccard.measure 0.70  projection.test.p.value 0  projection.test.lower.tail FALSE  relative.distances.ecdf.deviation.area.p.value "<0.01"  scaled.absolute.min.distance.sum.p.value "<0.01"  scaled.absolute.min.distance.sum.lower.tail TRUE  jaccard.measure.p.value "<0.01"  jaccard.measure.lower.tail FALSE | H3K4me3 within 1Kb downstream regions from major CAGE peak  chromosomes=chr1, chr2, chr3,chr4, chr5, chr6, chr7,chr8, chr9, chr10, chr11,chr12, chr13, chr14 , chr15,chr16, chr17, chr18 , chr19,chr20, chr21, chr22 , chrX,chrY  relative.distances.ks.p.value 20.201329e-05 0  relative.distances.ecdf.deviation.area -0.0323145  relative.distances.ecdf.area.correlation -0.6874329  intersection 1140  union 18864  jaccard.measure 0.06  projection.test.p.value 0.06  projection.test.lower.tail TRUE  relative.distances.ecdf.deviation.area.p.value ">0.01"  scaled.absolute.min.distance.sum.p.value ">0.01"  scaled.absolute.min.distance.sum.lower.tail FALSE  jaccard.measure.p.value ">0.01"  jaccard.measure.lower.tail TRUE |
| --- | --- |

**Supplementary File 2 table S5c.** GSC analysis for H3K4me3 enrichments within 1Kb up- and downstream regions surrounding the major CAGE cluster peak for intergenic EOTr candidates in HepG2 cells.

| H3K4me3 within 1Kb upstream regions from major CAGE peak  chromosomes=chr1, chr2, chr3,chr4, chr5, chr6, chr7,chr8, chr9, chr10, chr11,chr12, chr13, chr14 , chr15,chr16, chr17, chr18 , chr19,chr20, chr21, chr22 ,chrX, chrY  relative.distances.ks.p.value 1.101329e-06 0  relative.distances.ecdf.deviation.area 0.01367890  relative.distances.ecdf.area.correlation 0.126430  intersection 14582  union 21993  jaccard.measure 0.67  projection.test.p.value 0  projection.test.lower.tail FALSE  relative.distances.ecdf.deviation.area.p.value "<0.01"  scaled.absolute.min.distance.sum.p.value "<0.01"  scaled.absolute.min.distance.sum.lower.tail TRUE  jaccard.measure.p.value "<0.01"  jaccard.measure.lower.tail FALSE | H3K4me3 within 1Kb downstream regions from major CAGE peak  chromosomes=chr1, chr2, chr3,chr4, chr5, chr6, chr7,chr8, chr9, chr10, chr11,chr12, chr13, chr14 , chr15,chr16, chr17, chr18 , chr19,chr20, chr21, chr22 , chrX,chrY  relative.distances.ks.p.value 10.201329e-05 0  relative.distances.ecdf.deviation.area -0.0222579  relative.distances.ecdf.area.correlation -0.7327893  intersection 2199  union 21993  jaccard.measure 0.09  projection.test.p.value 0.06  projection.test.lower.tail TRUE  relative.distances.ecdf.deviation.area.p.value ">0.01"  scaled.absolute.min.distance.sum.p.value ">0.01"  scaled.absolute.min.distance.sum.lower.tail FALSE  jaccard.measure.p.value ">0.01"  jaccard.measure.lower.tail TRUE |
| --- | --- |

**Supplementary File 2 table S5d. GSC analysis for H3K4me3 enrichments within 1Kb up- and downstream regions surrounding the major CAGE cluster peak for intergenic EOTr candidates in HeLa cells.**

| H3K4me3 within 1Kb upstream regions from major CAGE peak  chromosomes=chr1, chr2, chr3,chr4, chr5, chr6, chr7,chr8, chr9, chr10, chr11,chr12, chr13, chr14 , chr15,chr16, chr17, chr18 , chr19,chr20, chr21, chr22, chrX,chrY    relative.distances.ks.p.value 2.1021345e-05 0  relative.distances.ecdf.deviation.area 0.01127893  relative.distances.ecdf.area.correlation 0.212987  intersection 19291  union 27451  jaccard.measure 0.70  projection.test.p.value 0  projection.test.lower.tail FALSE  relative.distances.ecdf.deviation.area.p.value <0.01  scaled.absolute.min.distance.sum.p.value <0.01  scaled.absolute.min.distance.sum.lower.tail TRUE  jaccard.measure.p.value <0.01  jaccard.measure.lower.tail FALSE | H3K4me3 within 1Kb downstream regions from major CAGE peak  chromosomes=chr1, chr2, chr3,chr4, chr5, chr6, chr7,chr8, chr9, chr10, chr11,chr12, chr13, chr14 , chr15,chr16, chr17, chr18 , chr19,chr20, chr21, chr22, chrX,chrY    relative.distances.ks.p.value 12.101343e-06 0  relative.distances.ecdf.deviation.area -1.01815026  relative.distances.ecdf.area.correlation -1.3245671  intersection 4393  union 27451  jaccard.measure 0.16  projection.test.p.value 0.10  projection.test.lower.tail TRUE  relative.distances.ecdf.deviation.area.p.value >0.01  scaled.absolute.min.distance.sum.p.value >0.01  scaled.absolute.min.distance.sum.lower.tail FALSE  jaccard.measure.p.value >0.01  jaccard.measure.lower.tail TRUE |
| --- | --- |

**Supplementary File 2 table S6. STAP (sequence to affinity prediction) analysis for c-Myc, c-Jun and BRCA1 transcription factor/DNA binding for intergenic enhancer domains with EOTrs and non-EOTrs in HepG2 and HeLa cell lines: maxBindingWts=PWM scores, inFactorIntMat=favourable (>1)/unfavourable (<1) binding and expRatios=Pearson’s correlation. The analysis revealed unfavourable binding for EOTr compared to non-EOTR intergenic regions and suggested the occlusion of TF-binding *via* TI. PWMs were similar for both data sets and confirmed that EOTr regions were not associated with TF-binding sites of particularly low affinity.**

| Cell line | Transcription factor | | maxBindingWts  non-EOTrs EOTr | | inFactorIntMat  non-EOTrs EOTr | | expRatios  non-EOTrs EOTr | |
| --- | --- | --- | --- | --- | --- | --- | --- | --- |
| HepG2 | 1. | c-Myc | 85.19 | 87.69 | 1.24 | 0.001 | 0.71 | 0.04 |
|  | 2. | c-Jun | 78.93 | 77.52 | 1.79 | 0.13 | 0.86 | 0.001 |
|  | 3. | BRCA1 | 84.98 | 81.69 | 1.38 | 0.02 | 0.67 | 0.034 |
|  | . |  |  |  |  |  |  |  |
| HeLa | 1. | c-Myc | 88.12 | 87.44 | 1.27 | 0.03 | 0.62 | 0.01 |
|  | 2. | c-Jun | 77.12 | 75.20 | 1.36 | 0.09 | 0.78 | 0.05 |
|  | 3. | BRCA1 | 88.79 | 87.97 | 1.57 | 0.22 | 0.68 | 0.3 |

**Supplementary File 2 table S7.** STAP (sequence to affinity prediction) analysis across cell lines for c-Myc, c-Jun and BRCA1 transcription factor/DNA binding in HepG2 and HeLa cell lines. Displayed are results for intergenic enhancer domains with cell line specific EOTrs (EOTr). Non-EOTr regions represent TF-binding within identical enhancer domains monitored in the absence of EOTr expression: maxBindingWts=PWM scores, inFactorIntMat=favourable (>1)/unfavourable (<1) binding and expRatios=Pearson’s correlation. The analysis revealed unfavourable binding for EOTr compared to non-EOTr intergenic regions and suggested the occlusion of TF-binding *via* TI. PWM were similar for both data sets and confirmed that EOTR regions were not associated with TF-binding sites of particularly low affinity.

| Cell line | Transcription factor | | maxBindingWts  non-EOTr EOTr | | inFactorIntMat  non-EOTr EOTr | | expRatios  non-EOTr EOTr | |
| --- | --- | --- | --- | --- | --- | --- | --- | --- |
| **HepG2 TFBS HeLa** | 1. | c-Myc | 81.11 | 84.29 | 1.84 | 0.01 | 0.62 | 0.02 |
|  | 2. | c-Jun | 86.23 | 84.12 | 1.39 | 0.03 | 0.66 | 0.3 |
|  | 3. | BRCA1 | 81.28 | 83.69 | 1.53 | 0.12 | 0.72 | 0.001 |
|  |  |  |  |  |  |  |  |  |
| **HeLa TFBS HepG2** | 1. | c-Myc | 74.12 | 76.44 | 1.27 | 0.001 | 0.71 | 0.01 |
|  | 2. | c-Jun | 66.12 | 69.20 | 1.47 | 0.11 | 0.75 | 0.012 |
|  | 3. | BRCA1 | 93.19 | 89.69 | 1.19 | 0.09 | 0.86 | 0.01 |

**Supplementary File 2 table S8a.** GSC analysis for H3K27ac enrichments for intronic EOTr PPRs and control regions in HepG2 cells (1Kb downstream regions).

| H3K27ac for EOTr PPRs  chromosomes=chr1, chr2, chr3,chr4, chr5, chr6, chr7,chr8, chr9, chr10, chr11,chr12, chr13, chr14 , chr15,chr16, chr17, chr18 , chr19,chr20, chr21, chr22 , chrX, chrY  relative.distances.ks.p.value 2.101329e-05 0  relative.distances.ecdf.deviation.area 0.01622012  relative.distances.ecdf.area.correlation 0.112987  intersection 17397  union 21479  jaccard.measure 0.81  projection.test.p.value 0  projection.test.lower.tail FALSE  relative.distances.ecdf.deviation.area.p.value <0.01  scaled.absolute.min.distance.sum.p.value <0.01  scaled.absolute.min.distance.sum.lower.tail TRUE  jaccard.measure.p.value <0.01  jaccard.measure.lower.tail FALSE | H3K27ac control regions  chromosomes=chr1, chr2, chr3,chr4, chr5, chr6, chr7,chr8, chr9, chr10, chr11,chr12, chr13, chr14 , chr15,chr16, chr17, chr18 , chr19,chr20, chr21, chr22 chrX, chrY  relative.distances.ks.p.value 8.101126e-06 0  relative.distances.ecdf.deviation.area -1.01215026  relative.distances.ecdf.area.correlation -1.2344852  intersection 1178  union 21479  jaccard.measure 0.008  projection.test.p.value 0.02  projection.test.lower.tail TRUE  relative.distances.ecdf.deviation.area.p.value >0.01  scaled.absolute.min.distance.sum.p.value >0.01  scaled.absolute.min.distance.sum.lower.tail FALSE  jaccard.measure.p.value >0.01  jaccard.measure.lower.tail TRUE |
| --- | --- |

**Supplementary File 2 table S8b.** GSC analysis for H3K27ac enrichments for intronic EOTr PPRs and control regions in HeLa cells (1Kb downstream regions).

| H3K27ac for EOTr PPRs  chromosomes=chr1, chr2, chr3,chr4, chr5, chr6, chr7,chr8, chr9, chr10, chr11,chr12, chr13, chr14 , chr15,chr16, chr17, chr18 , chr19,chr20, chr21, chr22 , chrX,chrY    relative.distances.ks.p.value 2.101231e-05 0  relative.distances.ecdf.deviation.area 0.01225014  relative.distances.ecdf.area.correlation 0.1112127  intersection 19012  union 24375  jaccard.measure 0.78  projection.test.p.value 0  projection.test.lower.tail FALSE  relative.distances.ecdf.deviation.area.p.value <0.01  scaled.absolute.min.distance.sum.p.value <0.01  scaled.absolute.min.distance.sum.lower.tail TRUE  jaccard.measure.p.value <0.01  jaccard.measure.lower.tail FALSE | H3K27ac control regions  chromosomes=chr1, chr2, chr3,chr4, chr5, chr6, chr7,chr8, chr9, chr10, chr11,chr12, chr13, chr14 , chr15,chr16, chr17, chr18 , chr19,chr20, chr21, chr22 , chrX,chrY      relative.distances.ks.p.value 41.1014129e-06 0  relative.distances.ecdf.deviation.area -1.01312007  relative.distances.ecdf.area.correlation -1.332124561  intersection 2574  union 24375  jaccard.measure 0.15  projection.test.p.value 0.1  projection.test.lower.tail TRUE  relative.distances.ecdf.deviation.area.p.value >0.01  scaled.absolute.min.distance.sum.p.value >0.01  scaled.absolute.min.distance.sum.lower.tail FALSE  jaccard.measure.p.value >0.01  jaccard.measure.lower.tail TRUE |
| --- | --- |

**Supplementary File 2 table S8c.** GSC analysis for H3K27ac enrichments for intergenic EOTr PPRs and control regions in HepG2 cells (1Kb downstream regions).

| H3K27ac for EOTr PPRs  chromosomes=chr1, chr2, chr3,chr4, chr5, chr6, chr7,chr8, chr9, chr10, chr11,chr12, chr13, chr14 , chr15,chr16, chr17, chr18 , chr19,chr20, chr21, chr22 chrX, chrY    relative.distances.ks.p.value 2.101329e-05 0  relative.distances.ecdf.deviation.area 0.01622012  relative.distances.ecdf.area.correlation 0.112987  intersection 6637  union 5376  jaccard.measure 0.81  projection.test.p.value 0  projection.test.lower.tail FALSE  relative.distances.ecdf.deviation.area.p.value <0.01  scaled.absolute.min.distance.sum.p.value <0.01  scaled.absolute.min.distance.sum.lower.tail TRUE  jaccard.measure.p.value <0.01  jaccard.measure.lower.tail FALSE | H3K27ac control regions  chromosomes=chr1, chr2, chr3,chr4, chr5, chr6, chr7,chr8, chr9, chr10, chr11,chr12, chr13, chr14 , chr15,chr16, chr17, chr18 , chr19,chr20, chr21, chr22 , chrX, chrY    relative.distances.ks.p.value 8.101126e-06 0  relative.distances.ecdf.deviation.area -1.01215026  relative.distances.ecdf.area.correlation -1.2344852  intersection 672  union 5376  jaccard.measure 0.008  projection.test.p.value 0.02  projection.test.lower.tail TRUE  relative.distances.ecdf.deviation.area.p.value >0.01  scaled.absolute.min.distance.sum.p.value >0.01  scaled.absolute.min.distance.sum.lower.tail FALSE  jaccard.measure.p.value >0.01  jaccard.measure.lower.tail TRUE |
| --- | --- |

**Supplementary File 2 table S8d.** GSC analysis for H3K27ac for intergenic EOTr PPRs and control regions in HeLa cells (1Kb downstream regions).

| H3K27ac for EOTr PPRs  chromosomes=chr1, chr2, chr3,chr4, chr5, chr6, chr7,chr8, chr9, chr10, chr11,chr12, chr13, chr14 , chr15,chr16, chr17, chr18 , chr19,chr20, chr21, chr22 chrX, chrY    relative.distances.ks.p.value 0.101329e-05 0  relative.distances.ecdf.deviation.area 0.015220214  relative.distances.ecdf.area.correlation 0.0112981  intersection 5309  union 6721  jaccard.measure 0.79  projection.test.p.value 0  projection.test.lower.tail FALSE  relative.distances.ecdf.deviation.area.p.value <0.01  scaled.absolute.min.distance.sum.p.value <0.01  scaled.absolute.min.distance.sum.lower.tail TRUE  jaccard.measure.p.value <0.01  jaccard.measure.lower.tail FALSE | H3K27ac control regions  chromosomes=chr1, chr2, chr3,chr4, chr5, chr6, chr7,chr8, chr9, chr10, chr11,chr12, chr13, chr14 , chr15,chr16, chr17, chr18 , chr19,chr20, chr21, chr22 , chrX, chrY    relative.distances.ks.p.value 9.101126e-06 0  relative.distances.ecdf.deviation.area -1.01120532  relative.distances.ecdf.area.correlation -1.21778214  intersection 74  union 6721  jaccard.measure 0.011  projection.test.p.value 0.02  projection.test.lower.tail TRUE  relative.distances.ecdf.deviation.area.p.value >0.01  scaled.absolute.min.distance.sum.p.value >0.01  scaled.absolute.min.distance.sum.lower.tail FALSE  jaccard.measure.p.value >0.01  jaccard.measure.lower.tail TRUE |
| --- | --- |

**Supplementary File 2 table S9a.** χ2-tests for the analysis of H3K27ac enrichments (peaks) within H3K4me1 domains spanning intronic EOTrs and peaks with H3K27ac beyond EOTr 3’ ends (TTS) within the same enhancer domain in HepG2 cells.

Chi-square with Yates correction = 272.5752 with 1 degree of freedom. The two-tailed p-value is less than 0.0001. The result is significant at p < 0.01.

| Marginal  Row  Totals | Category 1 |  | Category 2 | (Grand  Total) |
| --- | --- | --- | --- | --- |
| Number of H3K27ac peaks starting within enhancers genome wide | 19884 | Number of H3K27ac peaks starting within H3K4me1 within EOTr loci | 672 | 20556 |
| Number of H3K27ac peaks starting within enhancers genome wide | 19884 | Number of H3K27ac peaks starting within H3K4me1 without EOTrs | 1453 | 21337 |
| Totals | 39768 |  | 2125 | 41893 |

**Supplementary File 2 table S9b.** χ2-tests for the analysis of H3K27ac enrichments (peaks) within H3K4me1 domains spanning intronic EOTrs and peaks with H3K27ac beyond EOTr 3’ ends (TTS) within the same enhancer domain in HeLa cells.

Chi-square with Yates correction =549.4597 with 1 degree of freedom. The two-tailed p-value is less than 0.0001. The result is significant at p < 0.01.

| Marginal  Row  Totals | Category 1 |  | Category 2 | (Grand  Total) |
| --- | --- | --- | --- | --- |
| Number of H3K27ac peaks starting within enhancers genome wide | 21768 | Number of H3K27ac peaks starting within H3K4me1 within EOTr loci | 826 | 22594 |
| Number of H3K27ac peaks starting within enhancers genome wide | 21768 | Number of H3K27ac peaks starting within H3K4me1 without EOTrs | 2147 | 23915 |
| Totals | 43536 |  | 2973 | 46509 |

**Supplementary File 2 table S9c.** χ2-tests for the analysis of H3K27ac enrichments (peaks) within H3K4me1 domains spanning intergenic EOTrs and peaks with H3K27ac beyond EOTr 3’ ends (TTS) within the same enhancer domain in HepG2 cells.

Chi-square with Yates correction = 1210 with 1 degree of freedom. The two-tailed p-value is less than 0.0001. The result is significant at p < 0.01.

| Marginal  Row  Totals | Category 1 |  | Category 2 | (Grand  Total) |
| --- | --- | --- | --- | --- |
| Number of H3K27ac peaks starting within enhancers genome wide | 21384 | Number of H3K27ac peaks starting within H3K4me1 within EOTr loci | 1326 | 21210 |
| Number of H3K27ac peaks starting within enhancers genome wide | 21384 | Number of H3K27ac peaks starting within H3K4me1 without EOTrs | 4035 | 23919 |
| Totals | 42768 |  | 5361 | 48129 |

**Supplementary File 2 table S9d.** χ2-tests for the analysis of H3K27ac enrichments (peaks) within H3K4me1 domains spanning intergenic EOTrs and peaks with H3K27ac beyond EOTr 3’ ends (TTS) within the same enhancer domain in HeLa cells.

Chi-square with Yates correction = 329 with 1 degree of freedom. The two-tailed p-value is less than 0.0001. The result is significant at p < 0.01.

| Marginal  Row  Totals | Category 1 |  | Category 2 | (Grand  Total) |
| --- | --- | --- | --- | --- |
| Genome wide H3K27ac peaks within enhancers | 21384 | Number of H3K27ac peaks starting within H3K4me1 within EOTr loci | 1768 | 23152 |
| Genome wide H3K27ac peaks within enhancers | 21384 | Number of H3K27ac peaks starting within H3K4me1 without EOTrs | 3105 | 24489 |
| Totals | 42768 |  | 4873 | 47641 |

**5C interactome results:**

**Supplementary File 2 table S10a.** GSC analysis for H3K4me3 compared to CTCF enrichments for regions interacting with intronic EOTr- regulated enhancer domains in HepG2 cells.

| H3K4me3 within EOTr interacting regions  chromosomes=chr1, chr2, chr3,chr4, chr5, chr6, chr7,chr8, chr9, chr10, chr11,chr12, chr13, chr14 , chr15,chr16, chr17, chr18 , chr19,chr20, chr21, chr22 , chrX, chrY      relative.distances.ks.p.value 1.101329e-05 0  relative.distances.ecdf.deviation.area 0.0151123  relative.distances.ecdf.area.correlation 0.312587  intersection 422  union 521  jaccard.measure 0.81  projection.test.p.value 0  projection.test.lower.tail FALSE  relative.distances.ecdf.deviation.area.p.value <0.01  scaled.absolute.min.distance.sum.p.value <0.01  scaled.absolute.min.distance.sum.lower.tail TRUE  jaccard.measure.p.value <0.01  jaccard.measure.lower.tail FALSE | CTCF within EOTr interacting regions  chromosomes=chr1, chr2, chr3,chr4, chr5, chr6, chr7,chr8, chr9, chr10, chr11,chr12, chr13, chr14 , chr15,chr16, chr17, chr18 , chr19,chr20, chr21, chr22 , chrX, chrY      relative.distances.ks.p.value 14.156297e-06 0  relative.distances.ecdf.deviation.area -1.02924237  relative.distances.ecdf.area.correlation -1.4456321  intersection 78  union 521  jaccard.measure 0.15  projection.test.p.value 0.04  projection.test.lower.tail TRUE  relative.distances.ecdf.deviation.area.p.value >0.01  scaled.absolute.min.distance.sum.p.value >0.01  scaled.absolute.min.distance.sum.lower.tail FALSE  jaccard.measure.p.value >0.01  jaccard.measure.lower.tail TRUE |
| --- | --- |

**ChIA-PET interactome results:**

**Supplementary File 2 table S10b.** GSC analysis for H3K4me3 compared to CTCF enrichments for regions interacting with intronic EOTr-regulated enhancer domains in HeLa cells.

| H3K4me3 within EOTr interacting regions  chromosomes=chr1, chr2, chr3,chr4, chr5, chr6, chr7,chr8, chr9, chr10, chr11,chr12, chr13, chr14 , chr15,chr16, chr17, chr18 , chr19,chr20, chr21, chr22 , chrX,chrY      relative.distances.ks.p.value 2.10167e-05 0  relative.distances.ecdf.deviation.area 0.012478561  relative.distances.ecdf.area.correlation 0.213564  intersection 372  union 472  jaccard.measure 0.79  projection.test.p.value 0  projection.test.lower.tail FALSE  relative.distances.ecdf.deviation.area.p.value <0.01  scaled.absolute.min.distance.sum.p.value <0.01  scaled.absolute.min.distance.sum.lower.tail TRUE  jaccard.measure.p.value <0.01  jaccard.measure.lower.tail FALSE | CTCF within EOTr interacting regions  chromosomes=chr1, chr2, chr3,chr4, chr5, chr6, chr7,chr8, chr9, chr10, chr11,chr12, chr13, chr14 , chr15,chr16, chr17, chr18 , chr19,chr20, chr21, chr22 , chrX,chrY      relative.distances.ks.p.value 7.156497e-06 0  relative.distances.ecdf.deviation.area -1.076423118  relative.distances.ecdf.area.correlation -1.7796521  intersection 56  union 472  jaccard.measure 0.12  projection.test.p.value 0.02  projection.test.lower.tail TRUE  relative.distances.ecdf.deviation.area.p.value >0.01  scaled.absolute.min.distance.sum.p.value >0.01  scaled.absolute.min.distance.sum.lower.tail FALSE  jaccard.measure.p.value >0.01  jaccard.measure.lower.tail TRUE |
| --- | --- |

**5C interactome results:**

**Supplementary File 2 table S11a.** GSC analysis for H3K4me3 compared to CTCF enrichments for regions interacting with intergenic EOTr-regulated enhancer domains in HepG2 cells.

| H3K4me3 within EOTr interacting regions  chromosomes=chr1, chr2, chr3,chr4, chr5, chr6, chr7,chr8, chr9, chr10, chr11,chr12, chr13, chr14 , chr15,chr16, chr17, chr18 , chr19,chr20, chr21, chr22 , chrX,chrY      relative.distances.ks.p.value 1.101329e-05 0  relative.distances.ecdf.deviation.area 0.0151123  relative.distances.ecdf.area.correlation 0.312587  intersection 382  union 479  jaccard.measure 0.79  projection.test.p.value 0  projection.test.lower.tail FALSE  relative.distances.ecdf.deviation.area.p.value <0.01  scaled.absolute.min.distance.sum.p.value <0.01  scaled.absolute.min.distance.sum.lower.tail TRUE  jaccard.measure.p.value <0.01  jaccard.measure.lower.tail FALSE | CTCF within EOTr interacting regions  chromosomes=chr1, chr2, chr3,chr4, chr5, chr6, chr7,chr8, chr9, chr10, chr11,chr12, chr13, chr14 , chr15,chr16, chr17, chr18 , chr19,chr20, chr21, chr22 , chrX,chrY      relative.distances.ks.p.value 14.156297e-06 0  relative.distances.ecdf.deviation.area -1.02924237  relative.distances.ecdf.area.correlation -1.4456321  intersection 32  union 479  jaccard.measure 0.06  projection.test.p.value 0.04  projection.test.lower.tail TRUE  relative.distances.ecdf.deviation.area.p.value >0.01  scaled.absolute.min.distance.sum.p.value >0.01  scaled.absolute.min.distance.sum.lower.tail FALSE  jaccard.measure.p.value >0.01  jaccard.measure.lower.tail TRUE |
| --- | --- |

**ChIA-PET interactome results:**

**Supplementary File 2 table S11b.** GSC analysis for H3K4me3 compared to CTCF enrichments for regions interacting with intergenic EOTr-regulated enhancer domains in HeLa cells.

| H3K4me3 within EOTr interacting regions  chromosomes=chr1, chr2, chr3,chr4, chr5, chr6, chr7,chr8, chr9, chr10, chr11,chr12, chr13, chr14 , chr15,chr16, chr17, chr18 , chr19,chr20, chr21, chr22 , chrX, chrY      relative.distances.ks.p.value 2.10167e-05 0  relative.distances.ecdf.deviation.area 0.012478561  relative.distances.ecdf.area.correlation 0.213564  intersection 416  union 512  jaccard.measure 0.81  projection.test.p.value 0  projection.test.lower.tail FALSE  relative.distances.ecdf.deviation.area.p.value <0.01  scaled.absolute.min.distance.sum.p.value <0.01  scaled.absolute.min.distance.sum.lower.tail TRUE  jaccard.measure.p.value <0.01  jaccard.measure.lower.tail FALSE | CTCF within EOTr interacting regions  chromosomes=chr1, chr2, chr3,chr4, chr5, chr6, chr7,chr8, chr9, chr10, chr11,chr12, chr13, chr14 , chr15,chr16, chr17, chr18 , chr19,chr20, chr21, chr22 , chrX, chrY      relative.distances.ks.p.value 7.156497e-06 0  relative.distances.ecdf.deviation.area -1.076423118  relative.distances.ecdf.area.correlation -1.7796521  intersection 103  union 512  jaccard.measure 0.20  projection.test.p.value 0.02  projection.test.lower.tail TRUE  relative.distances.ecdf.deviation.area.p.value >0.01  scaled.absolute.min.distance.sum.p.value >0.01  scaled.absolute.min.distance.sum.lower.tail FALSE  jaccard.measure.p.value >0.01  jaccard.measure.lower.tail TRUE |
| --- | --- |

**5C interactome results:**

**Supplementary File 2 table S12a.** GSC analysis for poised and active promoters interacting with intronic EOTr-regulated enhancer domains in HepG2 cells.

| Poised promoters (Supplementary file Methods) within EOTr interacting regions  chromosomes=chr1, chr2, chr3,chr4, chr5, chr6, chr7,chr8, chr9, chr10, chr11,chr12, chr13, chr14 , chr15,chr16, chr17, chr18 , chr19,chr20, chr21, chr22 , chrX,chrY    relative.distances.ks.p.value 1.101329e-05 0  relative.distances.ecdf.deviation.area 0.01377541  relative.distances.ecdf.area.correlation 0.218762  intersection 458  union 521  jaccard.measure 0.88  projection.test.p.value 0  projection.test.lower.tail FALSE  relative.distances.ecdf.deviation.area.p.value <0.01  scaled.absolute.min.distance.sum.p.value <0.01  scaled.absolute.min.distance.sum.lower.tail TRUE  jaccard.measure.p.value <0.01  jaccard.measure.lower.tail FALSE | Active promoters (Supplementary file Methods) within EOTr interacting regions  chromosomes=chr1, chr2, chr3,chr4, chr5, chr6, chr7,chr8, chr9, chr10, chr11,chr12, chr13, chr14 , chr15,chr16, chr17, chr18 , chr19,chr20, chr21, chr22 , chrX,chrY      relative.distances.ks.p.value 18.156297e-06 0  relative.distances.ecdf.deviation.area -1.01814371  relative.distances.ecdf.area.correlation -1.2876537  intersection 57  union 521  jaccard.measure 0.11  projection.test.p.value 0.04  projection.test.lower.tail TRUE  relative.distances.ecdf.deviation.area.p.value >0.01  scaled.absolute.min.distance.sum.p.value >0.01  scaled.absolute.min.distance.sum.lower.tail FALSE  jaccard.measure.p.value >0.01  jaccard.measure.lower.tail TRUE |
| --- | --- |

**5C interactome results:**

**Supplementary File 2 table S12b.** GSC analysis for poised and active promoters interacting with intergenic EOTr-regulated enhancer domains in HepG2 cells.

| Poised promoters (Supplementary file Methods) within EOTr interacting regions  chromosomes=chr1, chr2, chr3,chr4, chr5, chr6, chr7,chr8, chr9, chr10, chr11,chr12, chr13, chr14 , chr15,chr16, chr17, chr18 , chr19,chr20, chr21, chr22 , chrX,chrY    relative.distances.ks.p.value 1.101329e-05 0  relative.distances.ecdf.deviation.area 0.01377541  relative.distances.ecdf.area.correlation 0.218762  intersection 521  union 672  jaccard.measure 0.77  projection.test.p.value 0  projection.test.lower.tail FALSE  relative.distances.ecdf.deviation.area.p.value <0.01  scaled.absolute.min.distance.sum.p.value <0.01  scaled.absolute.min.distance.sum.lower.tail TRUE  jaccard.measure.p.value <0.01  jaccard.measure.lower.tail FALSE | Active promoters (Supplementary file Methods) within EOTr interacting regions  chromosomes=chr1, chr2, chr3,chr4, chr5, chr6, chr7,chr8, chr9, chr10, chr11,chr12, chr13, chr14 , chr15,chr16, chr17, chr18 , chr19,chr20, chr21, chr22 , chrX,chrY      relative.distances.ks.p.value 18.156297e-06 0  relative.distances.ecdf.deviation.area -1.01814371  relative.distances.ecdf.area.correlation -1.2876537  intersection 143  union 672  jaccard.measure 0.21  projection.test.p.value 0.04  projection.test.lower.tail TRUE  relative.distances.ecdf.deviation.area.p.value >0.01  scaled.absolute.min.distance.sum.p.value >0.01  scaled.absolute.min.distance.sum.lower.tail FALSE  jaccard.measure.p.value >0.01  jaccard.measure.lower.tail TRUE |
| --- | --- |

**ChIA-PET interactome results:**

**Supplementary File 2 table S12c.** GSC analysis for poised and active promoters interacting with intronic EOTr-regulated enhancer domains in HeLa cells.

| Poised promoters (Supplementary file Methods) within EOTr interacting regions  chromosomes=chr1, chr2, chr3,chr4, chr5, chr6, chr7,chr8, chr9, chr10, chr11,chr12, chr13, chr14 , chr15,chr16, chr17, chr18 , chr19,chr20, chr21, chr22, chrX, chrY  relative.distances.ks.p.value 1.00167e-05 0  relative.distances.ecdf.deviation.area 0.014288661  relative.distances.ecdf.area.correlation 0.114212  intersection 373  union 472  jaccard.measure 0.79  projection.test.p.value 0  projection.test.lower.tail FALSE  relative.distances.ecdf.deviation.area.p.value <0.01  scaled.absolute.min.distance.sum.p.value <0.01  scaled.absolute.min.distance.sum.lower.tail TRUE  jaccard.measure.p.value <0.01  jaccard.measure.lower.tail FALSE | Active promoters (Supplementary file Methods) within EOTr interacting regions  chromosomes=chr1, chr2, chr3,chr4, chr5, chr6, chr7,chr8, chr9, chr10, chr11,chr12, chr13, chr14 , chr15,chr16, chr17, chr18 , chr19,chr20, chr21, chr22 , chrX, chrY      relative.distances.ks.p.value 7.156497e-06 0  relative.distances.ecdf.deviation.area -1.036212997  relative.distances.ecdf.area.correlation -1.1473201  intersection 56  union 472  jaccard.measure 0.12  projection.test.p.value 0.02  projection.test.lower.tail TRUE  relative.distances.ecdf.deviation.area.p.value >0.01  scaled.absolute.min.distance.sum.p.value >0.01  scaled.absolute.min.distance.sum.lower.tail FALSE  jaccard.measure.p.value >0.01  jaccard.measure.lower.tail TRUE |
| --- | --- |

**ChIA-PET interactome results:**

**Supplementary File 2 table S12d.** GSC analysis for poised and active promoters interacting with intergenic EOTr-regulated enhancer domains in HeLa cells.

| Poised promoters (Supplementary file Methods) within EOTr interacting regions  chromosomes=chr1, chr2, chr3,chr4, chr5, chr6, chr7,chr8, chr9, chr10, chr11,chr12, chr13, chr14 , chr15,chr16, chr17, chr18 , chr19,chr20, chr21, chr22 , chrX, chrY  relative.distances.ks.p.value 1.00167e-05 0  relative.distances.ecdf.deviation.area 0.014288661  relative.distances.ecdf.area.correlation 0.114212  intersection 673  union 835  jaccard.measure 0.80  projection.test.p.value 0  projection.test.lower.tail FALSE  relative.distances.ecdf.deviation.area.p.value <0.01  scaled.absolute.min.distance.sum.p.value <0.01  scaled.absolute.min.distance.sum.lower.tail TRUE  jaccard.measure.p.value <0.01  jaccard.measure.lower.tail FALSE | Active promoters (Supplementary file Methods) within EOTr interacting regions  chromosomes=chr1, chr2, chr3,chr4, chr5, chr6, chr7,chr8, chr9, chr10, chr11,chr12, chr13, chr14 , chr15,chr16, chr17, chr18 , chr19,chr20, chr21, chr22 , chrX, chrY      relative.distances.ks.p.value 7.156497e-06 0  relative.distances.ecdf.deviation.area -1.036212997  relative.distances.ecdf.area.correlation -1.1473201  intersection 256  union 835  jaccard.measure 0.30  projection.test.p.value 0.02  projection.test.lower.tail TRUE  relative.distances.ecdf.deviation.area.p.value >0.01  scaled.absolute.min.distance.sum.p.value >0.01  scaled.absolute.min.distance.sum.lower.tail FALSE  jaccard.measure.p.value >0.01  jaccard.measure.lower.tail TRUE |
| --- | --- |

**Supplementary File 2 table S13a.** GSC analysis for active and poised promoter states of RefSeq genes (hg19) harboring EOTrs within introns for HepG2 cells.

| Active promoter states (Supplementary file Methods) within 1Kb upstream from RefSeq TSS  chromosomes.to.proceed = c("chr1", "chr2", "chr3","chr4", "chr5", "chr6", "chr7","chr8", "chr9", "chr10", "chr11","chr12", "chr13", "chr14" , "chr15","chr16", "chr17", "chr18" , "chr19","chr20", "chr21", "chr22" , "chrX","chrY")  relative.distances.ks.p.value 16.021426e-06 0  relative.distances.ecdf.deviation.area -3.01443026  relative.distances.ecdf.area.correlation -1.342156  intersection 6021  union 29202  jaccard.measure 0.20  projection.test.p.value 0.02  projection.test.lower.tail TRUE  relative.distances.ecdf.deviation.area.p.value ">0.01"  scaled.absolute.min.distance.sum.p.value ">0.01"  scaled.absolute.min.distance.sum.lower.tail FALSE  jaccard.measure.p.value ">0.01"  jaccard.measure.lower.tail TRUE | Poised promoter states (Supplementary file Methods) within 1Kb upstream from RefSeq TSS  chromosomes.to.proceed = c("chr1", "chr2", "chr3","chr4", "chr5", "chr6", "chr7","chr8", "chr9", "chr10", "chr11","chr12", "chr13", "chr14" , "chr15","chr16", "chr17", "chr18" , "chr19","chr20", "chr21", "chr22" , "chrX","chrY")  relative.distances.ks.p.value 0.1017651e-05 0  relative.distances.ecdf.deviation.area 0.0134902  relative.distances.ecdf.area.correlation 0.122675  intersection 22874  union 29202  jaccard.measure 0.78  projection.test.p.value 0  projection.test.lower.tail FALSE  relative.distances.ecdf.deviation.area.p.value "<0.01"  scaled.absolute.min.distance.sum.p.value "<0.01"  scaled.absolute.min.distance.sum.lower.tail TRUE  jaccard.measure.p.value "<0.01"  jaccard.measure.lower.tail FALSE |
| --- | --- |

**Supplementary File 2 table S13b.** GSC analysis for active and poised promoter states of RefSeq genes (hg19) harboring EOTrs within introns for HeLa cells.

| Active promoter states (Supplementary file Methods) within 1Kb upstream from RefSeq TSS  chromosomes.to.proceed = c("chr1", "chr2", "chr3","chr4", "chr5", "chr6", "chr7","chr8", "chr9", "chr10", "chr11","chr12", "chr13", "chr14" , "chr15","chr16", "chr17", "chr18" , "chr19","chr20", "chr21", "chr22" , "chrX","chrY")  relative.distances.ks.p.value 10.201329e-05 0  relative.distances.ecdf.deviation.area -0.0222579  relative.distances.ecdf.area.correlation -0.7327893  intersection 2199  union 26543  jaccard.measure 0.08  projection.test.p.value 0.06  projection.test.lower.tail TRUE  relative.distances.ecdf.deviation.area.p.value ">0.01"  scaled.absolute.min.distance.sum.p.value ">0.01"  scaled.absolute.min.distance.sum.lower.tail FALSE  jaccard.measure.p.value ">0.01"  jaccard.measure.lower.tail TRUE | Poised promoter states (Supplementary file Methods) within 1Kb upstream from RefSeq TSS  chromosomes.to.proceed = c("chr1", "chr2", "chr3","chr4", "chr5", "chr6", "chr7","chr8", "chr9", "chr10", "chr11","chr12", "chr13", "chr14" , "chr15","chr16", "chr17", "chr18" , "chr19","chr20", "chr21", "chr22" , "chrX","chrY")    relative.distances.ks.p.value 1.101329e-06 0  relative.distances.ecdf.deviation.area 0.01367890  relative.distances.ecdf.area.correlation 0.126430  intersection 21582  union 26543  jaccard.measure 0.81  projection.test.p.value 0  projection.test.lower.tail FALSE  relative.distances.ecdf.deviation.area.p.value "<0.01"  scaled.absolute.min.distance.sum.p.value "<0.01"  scaled.absolute.min.distance.sum.lower.tail TRUE  jaccard.measure.p.value "<0.01"  jaccard.measure.lower.tail FALSE |
| --- | --- |

**Supplementary File 2 table S14a.** χ2-tests for the analysis of chromatin interaction loops within H3K4me1 domains spanning intronic EOTrs and RefSeq gene promoters in HepG2 cells.

Chi-square with Yates correction = 1052 with 1 degree of freedom. The two-tailed p-value is less than 0.0001. The result is significant at p < 0.01.

| Marginal  Row  Totals | Category 1 |  | Category 2 | (Grand  Total) |
| --- | --- | --- | --- | --- |
| Genome wide enhancers | 28884 | Number of chromatin loops for intronic enhancers associated with EOTrs interacting with host gene promoters | 231 | 29115 |
| Genome wide enhancers | 28884 | Number of chromatin loops for intronic enhancers without EOTrs interacting with host gene promoters | 1677 | 30551 |
| Totals | 57768 |  | 1898 | 59666 |

**Supplementary File 2 table S14b.** χ2-tests for the analysis of chromatin interaction loops within H3K4me1 domains spanning intronic EOTrs and RefSeq gene promoters in HeLa cells.

Chi-square with Yates correction = 713 with 1 degree of freedom. The two-tailed p-value is less than 0.0001. The result is significant at p < 0.01.

| Marginal  Row  Totals | Category 1 |  | Category 2 | (Grand  Total) |
| --- | --- | --- | --- | --- |
| Genome wide enhancers | 31724 | Number of chromatin loops for intronic enhancers associated with EOTrs interacting with host gene promoters | 668 | 32392 |
| Genome wide enhancers | 31724 | Number of chromatin loops for intronic enhancers without EOTrs interacting with host gene promoters | 2105 | 33829 |
| Totals | 63448 |  | 2773 | 66221 |
