# Supplementary material for: Enhancer occlusion transcripts regulate the activity of human enhancer domains via transcriptional interference: a computational perspective
Source: Nucleic Acids Res. 2020 Mar 5;48(7):3435–54. doi: 10.1093/nar/gkaa026 (PMC7144904; doi:10.1093/nar/gkaa026)
Supplement: gkaa026_Supplemental_Files [file gkaa026_supplemental_files.zip › Supplement_File_4_NAR_01_03_2020.doc]

| Page | |
| --- | --- |
| 2 - 3 | 4.1. EOTr RNA-Seq |
| 3 | Supplementary File 4 table S1a. |
| 4 | Supplementary File 4 table S1b. |
| 5 | Supplementary File 4 table S1c. |
| 6 | Supplementary File 4 table S1d. |
| 7 | References |

**4.1. EOTr RNA-Seq**

**EOTrs are capped transcripts - CAGE cluster analysis of candidate transcripts**

Methods for strand-specific RNA deep sequencing might suffer from the under-representation of transcript termini and potentially fail to accurately delineate the TSS. Therefore, the annotation of candidate EOTr 5’ termini was reconfirmed *via* the analysis of FANTOM (Functional Annotation of the Mammalian Genome) CAGE cluster libraries. CAGE clusters were selected according to pre-calculated hidden Markov models (HMMs), which allowed the identification of transcripts associated with *bona fide* promoter structures (1,2). The intersection CAGE clusters with proximal promoter regions of EOTr candidates (1Kb upstream to the GENCODE annotated TSS) was statistically validated *via* the GSC and the calculation of Jaccard indices (3,4) (Supplementary File 4 tables S1a-d). The number of CAGE clusters starting within PPRs was compared to those within 1Kb downstream regions from GENCODE annotated RNA 5’ termini. Jaccard indices and corresponding p-values (Table 2 and Supplementary File 4 table S1a-d) revealed the specific intersection of EOTr PPRs and CAGE cluster contigs; the combined 1Kb up- and downstream regions, served as union for calculation of the Jaccard indexes (Supplementary file Methods). The major peak within the PPRs of EOTrs was considered to represent the actual TSS.

**EOTrs are capped transcripts**

The intersection of EOTr candidates with a metric representing combined enrichments for H3K4me3 and H3K36me3 indicated that EOTrs utilize their own promoter for expression (Figure 1) (5-8). The localization of CAGE clusters within 1Kb upstream regions as defined by ENCODE cDNA contigs, enabled the identification of *bona fide* TSSs and the analysis of proximal promoter regions (PPR). All CAGE clusters were selected according to pre-calculated HMMs (Hidden Markov Models) for enrichment of TSSs connected to *bona fide* promoter structures. The association of EOTrs with CAGE clusters was finally tested *via* the genome structure correction, which confirmed that EOTrs are capped RNAs (Table 1) (Supplementary File 4 section 4.1 and Supplementary File 4 tables S1a-d).

**Supplementary File 4 table S1a.** GSC analysis for CAGE clusters starting within 1Kb regions up- and downstream from 5’ termini for intronic EOTr candidates in HepG2 cells.

| CAGE cluster tags within 1Kb upstream from cDNA contigs  chromosomes=chr1, chr2, chr3,chr4, chr5, chr6, chr7,chr8, chr9, chr10, chr11,chr12, chr13, chr14 , chr15,chr16, chr17, chr18 , chr19,chr20, chr21, chr22 , chrX,chrY    relative.distances.ks.p.value 3.401398e-06 0  relative.distances.ecdf.deviation.area 0.03115026  relative.distances.ecdf.area.correlation 0.1244852  intersection 122693  union 142667  jaccard.measure 0.86  projection.test.p.value 0  projection.test.lower.tail FALSE  relative.distances.ecdf.deviation.area.p.value <0.01  scaled.absolute.min.distance.sum.p.value <0.01  scaled.absolute.min.distance.sum.lower.tail TRUE  jaccard.measure.p.value <0.01  jaccard.measure.lower.tail FALSE | CAGE cluster tags within 1Kb downstream from cDNA contigs  chromosomes=chr1, chr2, chr3,chr4, chr5, chr6, chr7,chr8, chr9, chr10, chr11,chr12, chr13, chr14 , chr15,chr16, chr17, chr18 , chr19,chr20, chr21, chr22 , chrX,chrY    relative.distances.ks.p.value 8.101126e-06 0  relative.distances.ecdf.deviation.area -1.01815026  relative.distances.ecdf.area.correlation -1.2344852  intersection 17120  union 142667  jaccard.measure 0.12  projection.test.p.value 0.02  projection.test.lower.tail TRUE  relative.distances.ecdf.deviation.area.p.value >0.01  scaled.absolute.min.distance.sum.p.value >0.01  scaled.absolute.min.distance.sum.lower.tail FALSE  jaccard.measure.p.value >0.01  jaccard.measure.lower.tail TRUE |
| --- | --- |

**Supplementary File 4 table S1b.** GSC analysis for CAGE clusters starting within 1Kb upstream and downstream regions from 5’ termini for intronic EOTr candidates in HeLa cells.

| CAGE cluster tags 1Kb upstream regions from 5’ termini of cDNA contigs  chromosomes=chr1, chr2, chr3,chr4, chr5, chr6, chr7,chr8, chr9, chr10, chr11,chr12, chr13, chr14 , chr15,chr16, chr17, chr18 , chr19,chr20, chr21, chr22 , chrX,chrY    relative.distances.ks.p.value 3.201167e-06 0  relative.distances.ecdf.deviation.area 0.02815026  relative.distances.ecdf.area.correlation 0.1344852  intersection 114454  union 125774  jaccard.measure 0.91  projection.test.p.value 0  projection.test.lower.tail FALSE  relative.distances.ecdf.deviation.area.p.value <0.01  scaled.absolute.min.distance.sum.p.value <0.01  scaled.absolute.min.distance.sum.lower.tail TRUE  jaccard.measure.p.value <0.01  jaccard.measure.lower.tail FALSE | CAGE cluster tags within 1Kb downstream regions from 5’ termini of cDNA contigs  chromosomes=chr1, chr2, chr3,chr4, chr5, chr6, chr7,chr8, chr9, chr10, chr11,chr12, chr13, chr14 , chr15,chr16, chr17, chr18 , chr19,chr20, chr21, chr22 , chrX,chrY  relative.distances.ks.p.value 10.101126e-06 0  relative.distances.ecdf.deviation.area -1.02813027  relative.distances.ecdf.area.correlation -1.2233741  intersection 10061  union 125774  jaccard.measure 0.08  projection.test.p.value 0.03  projection.test.lower.tail TRUE  relative.distances.ecdf.deviation.area.p.value >0.01  scaled.absolute.min.distance.sum.p.value >0.01  scaled.absolute.min.distance.sum.lower.tail FALSE  jaccard.measure.p.value >0.01  jaccard.measure.lower.tail TRUE |
| --- | --- |

**Supplementary File 4 table S1c.** GSC analysis for CAGE clusters starting within 1Kb regions up- and downstream from 5’ termini for intergenic EOTr candidates in HepG2 cells.

| CAGE cluster tags 1Kb upstream regions from 5’ termini of cDNA contigs  chromosomes=chr1, chr2, chr3,chr4, chr5, chr6, chr7,chr8, chr9, chr10, chr11,chr12, chr13, chr14 , chr15,chr16, chr17, chr18 , chr19,chr20, chr21, chr22 , chrX,chrY      relative.distances.ks.p.value 3.401398e-06 0  relative.distances.ecdf.deviation.area 0.03115026  relative.distances.ecdf.area.correlation 0.1244852  intersection 48780  union 56721  jaccard.measure 0.86  projection.test.p.value 0  projection.test.lower.tail FALSE  relative.distances.ecdf.deviation.area.p.value <0.01  scaled.absolute.min.distance.sum.p.value <0.01  scaled.absolute.min.distance.sum.lower.tail TRUE  jaccard.measure.p.value <0.01  jaccard.measure.lower.tail FALSE | CAGE cluster tags within 1Kb downstream regions from 5’ termini of cDNA contigs  chromosomes=chr1, chr2, chr3,chr4, chr5, chr6, chr7,chr8, chr9, chr10, chr11,chr12, chr13, chr14 , chr15,chr16, chr17, chr18 , chr19,chr20, chr21, chr22 , chrX,chrY  relative.distances.ks.p.value 8.101126e-06 0  relative.distances.ecdf.deviation.area -1.01815026  relative.distances.ecdf.area.correlation -1.2344852  intersection 6806  union 56721  jaccard.measure 0.12  projection.test.p.value 0.02  projection.test.lower.tail TRUE  relative.distances.ecdf.deviation.area.p.value >0.01  scaled.absolute.min.distance.sum.p.value >0.01  scaled.absolute.min.distance.sum.lower.tail FALSE  jaccard.measure.p.value >0.01  jaccard.measure.lower.tail TRUE |
| --- | --- |

**Supplementary File 4 table S1d.** GSC analysis for CAGE clusters starting within 1Kb regions up- and downstream from 5’ termini for intergenic EOTr candidates in HeLa cells.

| CAGE cluster tags within 1Kb upstream regions from 5’ termini of cDNA contigs  chromosomes=chr1, chr2, chr3,chr4, chr5, chr6, chr7,chr8, chr9, chr10, chr11,chr12, chr13, chr14 , chr15,chr16, chr17, chr18 , chr19,chr20, chr21, chr22 , chrX, chrY    relative.distances.ks.p.value 3.201167e-06 0  relative.distances.ecdf.deviation.area 0.02815026  relative.distances.ecdf.area.correlation 0.1344852  intersection 55726  union 61238  jaccard.measure 0.91  projection.test.p.value 0  projection.test.lower.tail FALSE  relative.distances.ecdf.deviation.area.p.value <0.01  scaled.absolute.min.distance.sum.p.value <0.01  scaled.absolute.min.distance.sum.lower.tail TRUE  jaccard.measure.p.value <0.01  jaccard.measure.lower.tail FALSE | CAGE cluster tags within 1Kb downstream regions from 5’ termini of cDNA contigs  chromosomes=chr1, chr2, chr3,chr4, chr5, chr6, chr7,chr8, chr9, chr10, chr11,chr12, chr13, chr14 , chr15,chr16, chr17, chr18 , chr19,chr20, chr21, chr22 , chrX, chrY    relative.distances.ks.p.value 10.101126e-06 0  relative.distances.ecdf.deviation.area -1.02813027  relative.distances.ecdf.area.correlation -1.2233741  intersection 48990  union 61238  jaccard.measure 0.8  projection.test.p.value 0.03  projection.test.lower.tail TRUE  relative.distances.ecdf.deviation.area.p.value >0.01  scaled.absolute.min.distance.sum.p.value >0.01  scaled.absolute.min.distance.sum.lower.tail FALSE  jaccard.measure.p.value >0.01  jaccard.measure.lower.tail TRUE |
| --- | --- |

**References**

1.	Down, T.A. and Hubbard, T.J. (2002) Computational detection and location of transcription start sites in mammalian genomic DNA. *Genome research*, **12**, 458-461.

2.	Kodzius, R., Kojima, M., Nishiyori, H., Nakamura, M., Fukuda, S., Tagami, M., Sasaki, D., Imamura, K., Kai, C., Harbers, M. *et al.* (2006) CAGE: cap analysis of gene expression. *Nat Methods*, **3**, 211-222.

3.	Kodzius, R., Kojima, M., Nishiyori, H., Nakamura, M., Fukuda, S., Tagami, M., Sasaki, D., Imamura, K., Kai, C., Harbers, M. *et al.* (2006) CAGE: cap analysis of gene expression. *Nature methods*, **3**, 211-222.

4.	Favorov, A., Mularoni, L., Cope, L.M., Medvedeva, Y., Mironov, A.A., Makeev, V.J. and Wheelan, S.J. (2012) Exploring massive, genome scale datasets with the GenometriCorr package. *PLoS computational biology*, **8**, e1002529.

5.	Barski, A., Cuddapah, S., Cui, K., Roh, T.Y., Schones, D.E., Wang, Z., Wei, G., Chepelev, I. and Zhao, K. (2007) High-resolution profiling of histone methylations in the human genome. *Cell*, **129**, 823-837.

6.	Heintzman, N.D., Stuart, R.K., Hon, G., Fu, Y., Ching, C.W., Hawkins, R.D., Barrera, L.O., Van Calcar, S., Qu, C., Ching, K.A. *et al.* (2007) Distinct and predictive chromatin signatures of transcriptional promoters and enhancers in the human genome. *Nat Genet*, **39**, 311-318.

7.	Hon, G.C., Hawkins, R.D. and Ren, B. (2009) Predictive chromatin signatures in the mammalian genome. *Human molecular genetics*, **18**, R195-201.

8.	Guttman, M., Amit, I., Garber, M., French, C., Lin, M.F., Feldser, D., Huarte, M., Zuk, O., Carey, B.W., Cassady, J.P. *et al.* (2009) Chromatin signature reveals over a thousand highly conserved large non-coding RNAs in mammals. *Nature*, **458**, 223-227.
